# Exploration of Crucial Mediators for Carotid Atherosclerosis Pathogenesis Through Integration of Microbiome, Metabolome, and Transcriptome

**DOI:** 10.3389/fphys.2021.645212

**Published:** 2021-05-24

**Authors:** Lei Ji, Siliang Chen, Guangchao Gu, Jiawei Zhou, Wei Wang, Jinrui Ren, Jianqiang Wu, Dan Yang, Yuehong Zheng

**Affiliations:** ^1^Department of Vascular Surgery, Peking Union Medical College Hospital, Chinese Academy of Medical Sciences, Peking Union Medical College, Beijing, China; ^2^Medical Research Center, Peking Union Medical College Hospital, Chinese Academy of Medical Sciences, Peking Union Medical College, Beijing, China; ^3^Department of Computational Biology and Bioinformatics, Chinese Academy of Medical Sciences, Peking Union Medical College, Institute of Medicinal Plant Development, Beijing, China

**Keywords:** carotid atherosclerosis, microbiome, metabolome, transcriptome, correlation analysis

## Abstract

**Background:**

Carotid atherosclerosis (CAS) is an important cause of stroke. Although interactions between the gut microbiome and metabolome have been widely investigated with respect to the pathogenesis of cardiovascular diseases, information regarding CAS remains limited.

**Materials and Methods:**

We utilized 16S ribosomal DNA sequencing and untargeted metabolomics to investigate the alterations in the gut microbiota and plasma metabolites of 32 CAS patients and 32 healthy controls. The compositions of the gut microbiota differed significantly between the two groups, and a total of 11 differentially enriched genera were identified. In the metabolomic analysis, 11 and 12 significantly changed metabolites were screened in positive (POS) and negative (NEG) modes, respectively. α-N-Phenylacetyl-L-glutamine was an upregulated metabolite in CAS patients detected in both POS and NEG modes and had the highest | log_2_(fold change)| in POS mode. In addition, transcriptomic analysis was performed using the GSE43292 dataset.

**Results:**

A total of 132 differentially expressed genes (DEGs) were screened. Among the upregulated DEGs in CAS patients, FABP4 exhibited the highest | log_2_(fold change)|. Furthermore, FABP4 was positively associated with *Acidaminococcus* and had the highest Spearman’s correlation coefficient and the most significant *p*-value among the microbiota–DEG pairs.

**Conclusion:**

In this study, we investigated the potential “microbiota–metabolite–gene” regulatory axis that may act on CAS, and our results may help to establish a theoretical basis for further specialized study of this disease.

## Introduction

Atherosclerosis (AS) is a diffuse, slowly progressing disease that affects large- and medium-sized arteries. Advanced atherosclerotic plaques can invade the arterial lumen, impeding blood flow, and resulting in tissue ischemia (Faxon et al., 2004; Libby et al., 2019). Carotid atherosclerosis (CAS) is a preventable cause of 20–30% of stroke, approximately 21% of people aged 30–79 years have carotid plaque, and 1.5% have carotid stenosis (Petty et al., 2000; Song et al., 2020). The pathophysiological features of AS are primarily linked to lipid accumulation, chronic inflammation, calcification, and thrombosis (Libby et al., 2019). Many studies have vastly improved our understanding of the pathogenesis of AS, but, despite these advances, we still lack definitive evidence to translate basic results to the bedside (Weber and Noels, 2011). Although AS is a systemic disease sharing common major risk factors, differences exist in the strength and impact per arterial site (Aboyans et al., 2018). Medical interventions that result in the prevention of CAS are especially centered on statins, but which are not targeted enough when CAS is regarded as a unique form of AS (Artom et al., 2014).

Findings from the past decade have suggested that the structure and composition of the gut microbiota are associated with AS in humans and animal models (Jonsson and Backhed, 2017). The contributions of the gut microbiota to AS can be divided into three main categories. First, local or distant infections might aggravate atherogenesis. Second, patients with AS have altered lipid metabolism, and bacterial taxa in the gut were observed to correlate with plasma cholesterol levels (Koren et al., 2011). Third, diet and specific components that are metabolized by gut microbiota can have various effects on AS. Metabolites filtered or produced by gut microbiota, such as trimethylamine-N-oxide, short-chain fatty acids (SCFAs), and secondary bile acids, have been observed to affect the development of AS (Wang et al., 2011; Wahlstrom et al., 2016; Chen et al., 2018). Most studies of the relationship between CAS and microbiota could be classified into the first category mentioned earlier. A wide variety of microbial DNA has accordingly been found in carotid atherosclerotic plaques in different populations (Ziganshina et al., 2016; Lindskog Jonsson et al., 2017). Bacteria observed in the atherosclerotic plaques are also detected at other body sites, predominantly the gut, which might thus serve as reservoirs of these potentially pathogenic microorganisms (Jonsson and Backhed, 2017). However, limited information is available focusing on the gut microbiota composition in CAS patients. With respect to metabolomics, several studies have found a number of metabolites associated with CAS on the different stages (Vojinovic et al., 2018; Lee T. H. et al., 2019), which were used as non-causal biomarkers, but further study is necessary to elucidate the pathogenesis of CAS. Also, considerable uncertainty remains concerning the relationship between CAS and metabolites.

Taken together, both human and animal studies have indicated that alterations of the gut microbiota and plasma metabolites might be involved in the progression of AS, but the details of these alterations in patients with CAS have not been fully characterized. To address this question, we performed multi-omics combined 16S ribosomal DNA (rDNA) gene sequencing using fecal samples and untargeted liquid chromatography–mass spectrometry using plasma samples from 32 CAS patients and 32 healthy controls with gene expression profiling from the Gene Expression Omnibus (GEO) database to characterize the gut microbial community and plasma metabolic profiles. Also, we performed an integrated analysis of the microbiome, metabolome, and transcriptome. These results may ultimately provide a more in-depth understanding of the “microbiota–metabolite–gene” axis in the pathogenesis of CAS.

## Materials and Methods

### Medical Ethics

The Ethics Committee of the Peking Union Medical College Hospital (PUMCH) has approved this study (institutional approval number: JS-2629). Each participant provided signed informed consent before participating in the present study.

### Patients Recruitment

CAS patients were recruited from the Department of Vascular Surgery, Peking Union Medical College Hospital. The inclusion criteria for recruitment were as follows: (1) Diagnosis with carotid atherosclerosis by ultrasound or CT angiography; (2) age ≥ 45 years; the exclusion criteria were applied to both CAS patients and healthy controls: (1) Antibiotic usage within 6 months; (2) probiotic usage within 6 months; (3) history of gastrointestinal diseases (such as inflammatory bowel disease); (4) history of abdominal surgery (such as gastrectomy); (5) major dietary change 1 week before sample collection.

We first recruited 71 CAS patients and 39 healthy controls. Next, 39 CAS patients were excluded due to antibiotic usage (*n* = 14), probiotic usage (*n* = 6), digestive disease (*n* = 8), and abdominal surgery (*n* = 11). Meanwhile, seven healthy controls were excluded due to antibiotic usage (*n* = 4), probiotic usage (*n* = 2), and abdominal surgery (*n* = 1). Finally, each group had 32 subjects for further analysis.

### Sample Collection

Peripheral blood and stool samples were collected in the morning after an overnight fast (≥ 8 h). Plasma samples were obtained by centrifugation at 3,000 rpm for 10 min at room temperature. All plasma and stool samples were rapidly frozen and stored at −80°C until analysis.

### Genomic DNA Extraction and 16S Ribosomal DNA Sequencing

Genomic DNA extraction was performed using QIAamp^®^ Fast DNA Stool Mini Kit (Qiagen, Hilden, Germany) and examined using Thermo NanoDrop 2000 (Thermo Fisher Scientific, New York, NY, United States). The V3-V4 region of the bacterial 16S rDNA was amplified using KAPA HiFi Hotstart ReadyMix PCR Kit (KAPA Biosystems, Wilmington, MA, United States) with the primers 314F (CCTACGGGRSGCAGCAG) and 806R (GGACTACVVGGGTATCTAATC) and sequenced using an Illumina PE250 platform (Illumina, California, United States).

### Ultra-High-Performance Liquid Tandem Chromatography/Quadrupole Time-of-Flight Mass Spectrometry Metabolomic Profiling of Patient Plasma Samples

Plasma samples of patients were prepared for ultra-high-performance liquid tandem chromatography/quadrupole time-of-flight mass spectrometry (UHPLC-QTOFMS) analysis by application of validated protocols (Dunn et al., 2011). The UHPLC separation was carried out using a 1290 Infinity series UHPLC System (Agilent Technologies Inc., Santa Clara, California, United States), equipped with a UPLC BEH Amide column. The TripleTOF 6600 mass spectrometry (AB Sciex, Foster City, CA, United States) was used for its ability to acquire tandem mass spectrometry spectra on an information-dependent basis during a liquid chromatography–mass spectrometry experiment. Both positive ion mode (POS) and negative ion mode (NEG) were used to obtain maximal coverage for plasma metabolites.

### Transcriptomic Profiling of Atherosclerotic Samples From the Gene Expression Omnibus Database

To have a comprehensive understanding of CAS pathogenesis from a multi-omics perspective, we also acquired transcriptomic profiling data of CAS samples from the GEO database (GSE43292) (Edgar et al., 2002). The transcriptomic dataset was not measured from the same cohort of patients from whom the 16S and metabolomic datasets were generated. The probes in the series matrix file were annotated by gene symbols using the platform data table (GPL6244), and a gene expression matrix was obtained for further transcriptomic analysis.

### Statistical Analysis

Operational taxonomic units (OTUs) were obtained by ultra-fast sequence analysis (USEARCH) v11.0 with a sequence similarity of 0.97 (Edgar, 2013). α- and β-diversities were calculated using Quantitative Insights Into Microbial Ecology (QIIME, version 1.7.0) based on OTU counts (Caporaso et al., 2010). The “vegan” package in R version 3.6.2 was used to perform a permutational multivariate analysis of variance (PERMANOVA) to compare β-diversity between the two groups. Next, we performed a differential abundance analysis using the linear discriminant analysis (LDA) method on the LDA effect size (LEfSe) platform and the Wilcoxon rank-sum test (Segata et al., 2011). To determine the functional alterations in the gut microbiota of CAS patients, Kyoto Encyclopedia of Genes and Genomes (KEGG) pathway analysis was conducted through phylogenetic investigation of communities by reconstruction of unobserved states (PICRUSt) to predict the functional composition profiles of microbiota based on OTUs (Langille et al., 2013).

UHPLC-QTOFMS data were analyzed by SMICA (version 15.0.2, Sartorius Stedim Data Analytics AB, Umea, Sweden) to conduct multivariate statistical analysis. Differential metabolites were obtained by comparing CAS patients and healthy controls using a *t-*test. KEGG pathway analysis was also conducted for these different metabolites.

For transcriptomic profiling data, differentially expressed gene (DEG) analysis was performed based on gene expression matrix using the “limma” R package. A | log_2_(fold-change)| of > 1 and an adjusted *p*-value < 0.01 were selected as the threshold for DEG screening. In addition to KEGG pathway analysis, gene ontology-biological process (GO-BP) analysis was conducted using the Database for Annotation, Visualization and Integrated Discovery version 6.8^1^; the enrichment analysis was also conducted using Reactome version 75^2^ to further demonstrate the biological functions of DEGs.

To integrate the multi-omics data, Spearman’s correlation indices between differential omics data were calculated and visualized by heatmap (Shannon et al., 2003). Finally, receiver operating characteristic (ROC) analysis was performed using Statistical Product and Service Solutions version 25.0 (SPSS Inc., 2017, Chicago, IL, United States). Random forest (RF) analysis was conducted using the Biomarker analysis section of MetaboAnalyst version 3.0 (www.metaboanalyst.ca). The area under the curve was calculated to demonstrate the potential diagnostic value of differentially enriched genera, metabolites, and genes.

To further improve the accuracy of the analyses of microbiome and metabolome, the adjustment for covariates in differential genera and metabolites was performed. First, in the microbiome analysis, the associations between genera and clinical characteristics of CAS patients and healthy controls were evaluated using a generalized linear model, and *p* < 0.05 was considered to be statistically significant (Qian et al., 2018). Second, in the metabolome analysis, PERMANOVA was used to test the statistically significant differences between metabolic profiles and clinical characteristics. The *p*-value was corrected for multiple tests using a cutoff of 0.05.

## Results

### Flowchart of Our Study

**FIGURE 1:**
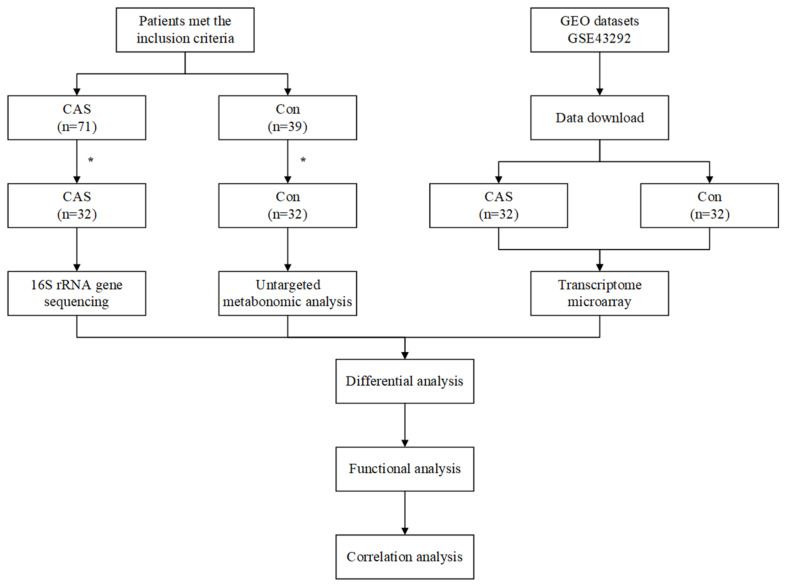
Workflow of our study. *A total of 39 patients were exclude in CAS Group, due to antibiotic usage (*n* = 14), probiotic usage (*n* = 6), digestive disease (*n* = 8), and abdominal surgery (*n* = 11). A total of seven healthy controls were excluded due to antibiotic usage (*n* = 4), probiotic usage (*n* = 2), and abdominal surgery (*n* = 1).

The workflow of our study is shown in Figure 1. A total of 32 CAS patients and 32 healthy controls were included in our study. Fecal and plasma samples were taken for microbiome and metabolome analysis, respectively. Differentially enriched microbiota and metabolites were identified. KEGG pathways were predicted to show the functional composition profiles of differentially enriched microbiota and metabolites. Then, DEG and related functional annotation analyses were conducted based on a messenger RNA (mRNA) microarray dataset (GSE43292) to explore the differences between CAS patients and healthy controls from a transcriptomic level. Furthermore, correlation analyses were performed between differentially enriched microbiota, metabolites, and DEGs to integrate omics. Finally, the potential clinical significance of differentially enriched microbiota, metabolites, and DEGs was determined by ROC and RF analyses.

### Clinical Characteristics of Carotid Atherosclerosis Patients and Controls

For microbiome and metabolome analysis, 64 fecal and plasma samples were used for 16S rDNA sequencing and untargeted metabolomic analysis (UHPLC-QTOFMS). The baseline of our study cohort is shown in Table 1. Although the body mass index is marginally higher in the CAS group (24.7 ± 2.7 for CAS patients and 23.2 ± 2.2 for healthy controls, *p* = 0.047), there were no significant differences in age and sex between CAS patients and healthy controls.

**TABLE 1:** Characteristics of study cohort.

	Control (*n* = 32)	CAS (*n* = 32)	*p*-value
Age (years)	66.2 ± 4.8	64.5 ± 6.7	0.263
Male (%)	28 (87.5)	28 (87.5)	>0.999
BMI (kg/m^2^)	23.2 ± 2.2	24.7 ± 2.7	0.047*
Hypertension (%)	6 (18.8)	12 (37.5)	0.095
Diabetes (%)	3 (9.4)	6 (18.8)	0.281
Coronary heart disease (%)	0 (0)	9 (28.1)	< 0.001*
White blood cell (× 10^9^/L)	6.5 ± 1.3	6.2 ± 1.5	0.420
Monocyte (× 10^9^/L)	0.37 ± 0.15	0.38 ± 0.10	0.136
Hcy (μmol/L)	16.5 ± 7.0	16.6 ± 6.3	0.958
TC (mmol/L)	4.5 ± 1.3	3.2 ± 0.7	< 0.001*
TG (mmol/L)	1.3 ± 0.8	1.2 ± 0.6	0.754
HDL-C (mmol/L)	1.3 ± 0.3	1.0 ± 0.2	< 0.001*
LDL-C (mmol/L)	2.9 ± 0.6	1.8 ± 0.6	< 0.001*

Normally distributed variables between two groups were analyzed by Student’s t-test. Mann–Whitney U test was applied for data of this type that were not normally distributed. χ^2^-Square test or Fisher’s exact test compared categorical variables. *p < 0.05.

### Microbial Profiling of Carotid Atherosclerosis Patients and Controls

#### Gut Microbiota Richness, Composition, and Diversity

**FIGURE 2:**
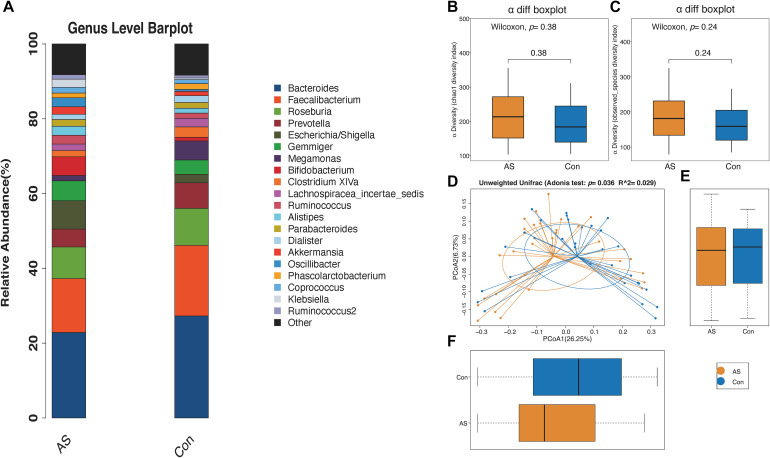
Microbial diversity of CAS patients and healthy controls. **(A)** Relative abundance of gut microbiota for two groups from genus level. **(B,C)** α-Diversity (chao1 and observed_species) for two groups showed no significant difference. **(D–F)** β-Diversity is significantly increased for CAS patients.

We used 2,300,644 high-quality reads from 64 patients for downstream analysis. The rarefaction curves of richness (observed_species and chao1) were plotted. Curves for the CAS and control groups were near saturation as the reads increased, suggesting that the sequencing depth was adequate (Supplementary Figures 1A,B). The Venn diagram showed overlapping and different enriched OTUs in each group (Supplementary Figure 1C). Next, OTUs were annotated using the Ribosomal Database Project database^3^, and the relative abundance of the gut microbiota is shown (Figure 2A and Supplementary Figures 1D–G).

The microbial α-diversity is shown in Supplementary Table 1. The Wilcoxon rank-sum test compared the α-diversity between the two groups, and no significant difference was found, which was also consistent with the rarefaction curve (Figures 2B,C). However, the gut microbiota communities between the CAS group and healthy control group were significantly different, as shown by β-diversity (Figures 2D–F).

### Differential Gut Microbiota Enriched in Carotid Atherosclerosis Patients and Healthy Controls

**FIGURE 3:**
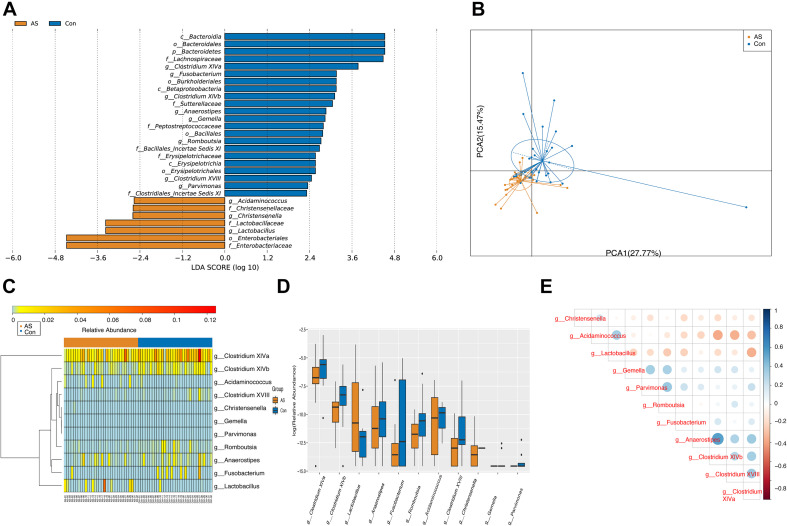
Differentially enriched gut microbiota for CAS patients and healthy controls. **(A)** A total of 29 different features screened by LEfSe at phylum (*n* = 1), class (*n* = 3), order (*n* = 5), family (*n* = 9), and genus (*n* = 11) level with a threshold of LDA > 2. **(B)** PCA plot demonstrated that CAS group is significantly different from control group based on differential genera. **(C,D)** Differentially enriched gut microbiotas were visualized in heatmap and box plot. **(E)** Associations between differential microbiotas were by correlation heatmap. Bluer color indicates a more positive correlation, whereas redder color indicates a more negative correlation.

Using LEfSe analysis, we screened 29 different features at the phylum (*n* = 1), class (*n* = 3), order (*n* = 5), family (*n* = 9), and genus (*n* = 11) levels with a threshold of LDA > 2 (Figure 3A). The Wilcoxon rank-sum test was also used to explore changes in microbiota, and 30 differentially enriched taxa were identified (Figures 3B–D, Supplementary Figure 2, and Supplementary Table 2). The differential microbiota at the genus level from the Wilcoxon test were the same as those that we had screened using LEfSe analysis (Table 2). *Acidaminococcus*, *Christensenella*, and *Lactobacillus* were enriched in CAS patients; *Anaerostipes*, *Fusobacterium*, *Gemella*, *Parvimonas*, *Romboutsia*, and *Clostridium* XVIII/XlVa/XlVb were enriched in healthy controls. The correlation between different genera was shown by Spearman’s correlation test (Figure 3E), and these microbiota genera were further utilized in correlation analysis with differential metabolites and DEGs.

**TABLE 2:** Differentially enriched gut microbiota from genera level.

Gut microbiota	Mean (AS)	Mean (Con)	*p*-value	Median (AS)	Median (Con)
g__Acidaminococcus	0.000880977	0.00010395	0.004853071	−10.30582176	−9.853309555
g__Anaerostipes	0.001823025	0.00227001	0.033041905	−11.23182118	−10.38382427
g__Christensenella	4.28794E-05	3.89813E-06	0.027190901	−13.55374927	−12.96878677
g__Clostridium XVIII	0.000165021	0.000632796	0.008816879	−12.96878677	−12.23182118
g__Clostridium XlVa	0.014102131	0.024426975	0.011581483	−6.754083402	−5.596422402
g__Clostridium XlVb	0.001576143	0.00431263	0.000456455	−9.352776212	−8.268347055
g__Fusobacterium	0.000284563	0.002597453	0.043528042	−13.55374927	−12.39278523
g__Gemella	3.89813E-06	2.079E-05	0.014781178	−14.55374927	−14.55374927
g__Lactobacillus	0.004282744	0.000174116	0.001399704	−10.74639435	−11.96878677
g__Parvimonas	1.29938E-06	1.68919E-05	0.012301771	−14.55374927	−14.55374927
g__Romboutsia	0.000267672	0.001351351	0.000467579	−11.74639435	−10.55657256

*Acidaminococcus, Christensenella, and Lactobacillus were enriched in CAS patients, whereas other genera were enriched in healthy controls.*

### Different Functional Composition Profiles of Gut Microbiota Between Carotid Atherosclerosis Patients and Healthy Controls

**FIGURE 4:**
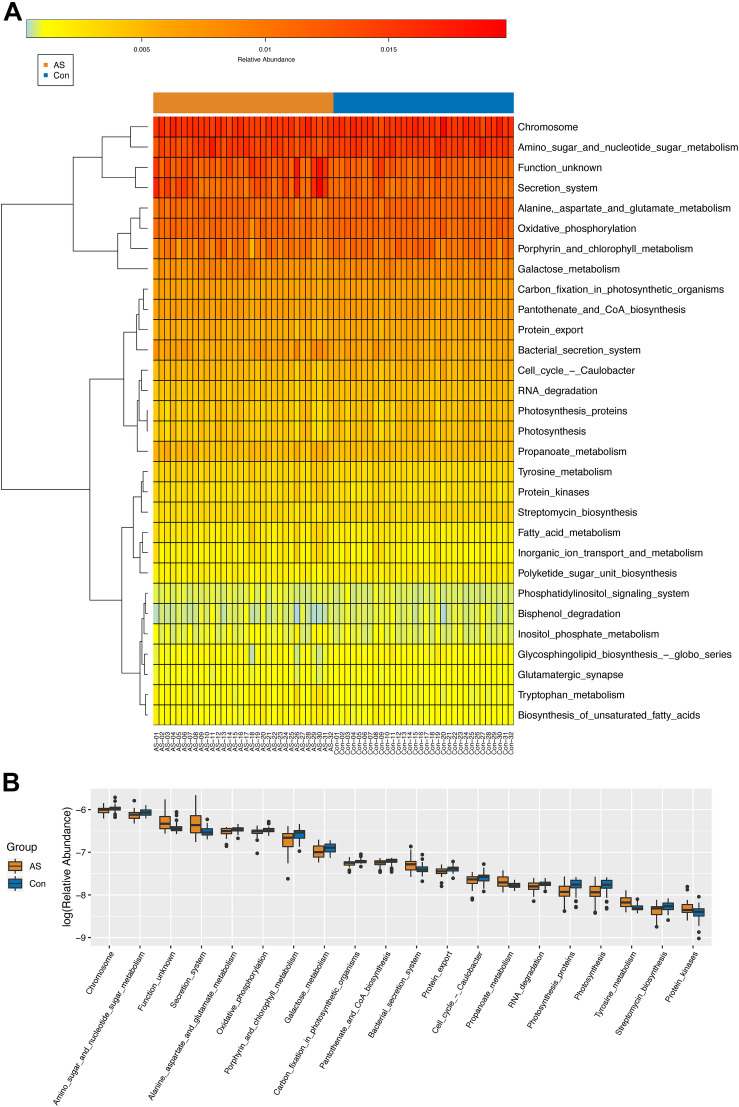
KEGG pathways (level 3) predicted by PICRUSt. **(A,B)** Differential KEGG pathways with top relative abundance were visualized as heatmap and box plot. Warmer color indicates higher relative abundance, whereas colder color indicates lower relative abundance.

Through PICRUSt, functional composition profiles of gut microbiota were predicted based on relative abundance and compared between CAS patients and healthy controls. In total, 65 of 265 differentially enriched KEGG pathways (level 3) were identified (Supplementary Table 3) with a threshold of *p* < 0.05, of which 39 were enriched in the CAS group, whereas 26 were enriched in healthy controls. Different pathways with the highest relative abundance were visualized by heatmap and boxplot (Figure 4).

### Metabolic Profiling of Carotid Atherosclerosis Patients and Controls

In total, 1,425 and 1,580 peaks were detected for the POS and NEG modes of UHPLC-QTOFMS, respectively, after filtering internal standards and pseudo-positive peaks.

### Differential Metabolites Screening

**FIGURE 5:**
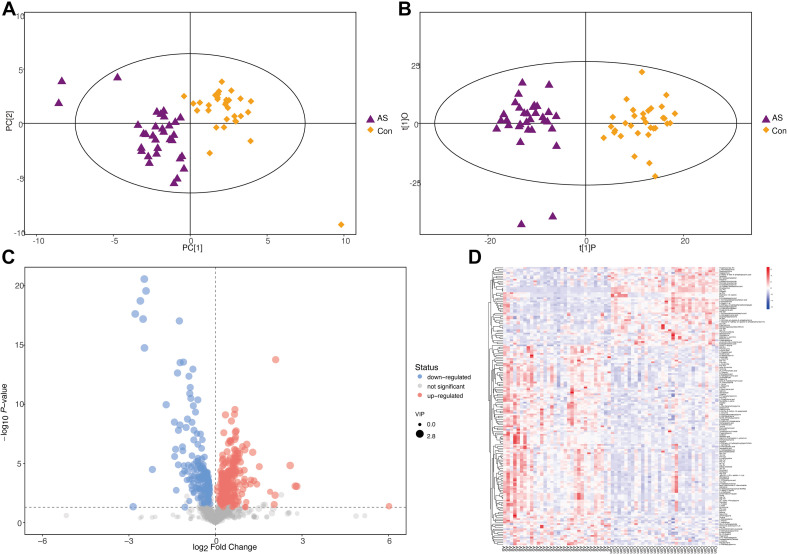
Differential metabolites screening for CAS patients and healthy controls (POS mode). **(A,B)** PCA and orthogonal projections to latent structures-discriminant analysis showed that CAS patients and healthy controls could be clearly separated. **(C,D)** Patterns of differential metabolites in POS mode were demonstrated by volcano plot and heatmap.

Two multivariate statistical analysis methods, principal component analysis (PCA) and orthogonal projections to latent structures-discriminant analysis, were utilized to classify plasma samples. Both methods showed that plasma samples for CAS patients and controls were clearly separated (Figures 5A,B and Supplementary Figures 3A,B). In addition, the permutation test indicated that the orthogonal projections to latent structures-discriminant analysis model is valid and that no overfitting exists (Supplementary Figures 3C,D).

With the thresholds of VIP > 1 and *p* < 0.05, 165 and 96 significantly changed metabolites were screened in POS and NEG modes, respectively (Supplementary Figure 3E and Supplementary Table 4). The patterns of differential metabolism were visualized by heatmaps and volcano plots (Figures 5C,D and Supplementary Figures 3F,G). Next, we added | log_2_(fold-change)| > 1 as another threshold and combined POS and NEG modes to select differential metabolites (Table 3) for correlation analysis with different omics data. With the addition of this threshold, 11 and 12 metabolites were screened in POS and NEG modes, respectively. PAGln, upregulated in CAS patients, was the only metabolite detected in both modes and had the highest | log_2_(fold-change)| in POS mode.

**TABLE 3:** Differential metabolites for correlation analysis.

Metabolites	VIP	*p*-value	*q*-value	Log_2_ (fold-change)
**POS mode**				–
Ethanolamine	2.816546298	6.3964E-18	8.96521E-16	−2.509502083
Gly-Pro	2.796795801	1.88336E-19	4.55375E-17	−2.603752686
Propoxur	2.785113538	2.69771E-20	9.78414E-18	−2.406871344
Homocitrate	1.978541617	0.000652259	0.001823948	1.047764919
α-N-Phenylacetyl-L-glutamine	1.762051456	0.000569636	0.001635096	1.436216381
Diethylcarbamazine	1.642884521	0.000557875	0.001607395	1.208993076
Dimethylbenzimidazole	2.347024371	7.03818E-07	9.59896E-06	1.016050071
Eicosapentaenoic acid	1.807845477	4.49425E-05	0.00025414	−1.07267148
Decanoyl-L-carnitine	1.911522868	1.30652E-05	9.80438E-05	−1.289227648
3-Methoxy-4-Hydroxyphenylglycol Sulfate	1.673158751	0.000206213	0.000749297	1.007090541
O-Desmethylnaproxen	2.140136711	9.1044E-09	2.26664E-07	−1.017466992
**NEG mode**				
Salicylic acid	1.734352898	0.01921337	0.0277024	4.145007415
3-Aminopropanesulphonic Acid	1.039813313	0.040379565	0.047806112	1.796022962
6-Hydroxynicotinic acid	1.206818152	0.029194166	0.037505435	2.079415637
Formylanthranilic acid	1.949132714	0.000220127	0.000651909	1.426589827
Xanthopterin	2.153639262	5.0361E-11	1.65249E-09	−1.016011938
N1-Methyl-4-pyridone-3-carboxamide	2.443377406	8.40619E-14	3.7485E-12	−1.180267083
3-Hydroxydodecanoic acid	1.872339097	5.84638E-05	0.000209662	−1.103961553
Salicyluric acid	1.428929548	0.027925205	0.036246094	1.744879658
Phenylacetylglycine	1.609315395	1.20649E-07	1.48763E-06	1.576664728
D-Biotin	2.385066256	4.85697E-11	1.60101E-09	−2.067524087
α-N-Phenylacetyl-L-glutamine	1.701961669	0.000390232	0.001051251	1.214340063
5,10-methylene-THF	1.922624936	8.93527E-07	8.64096E-06	−1.040847640

*A threshold of | log_2_(fold-change)| > 1 was added based on basic threshold of VIP > 1 and p-value < 0.05 to further screen metabolites for correlation analysis.*

### Metabolic Pathway Analysis for Differential Plasma Metabolites

**FIGURE 6:**
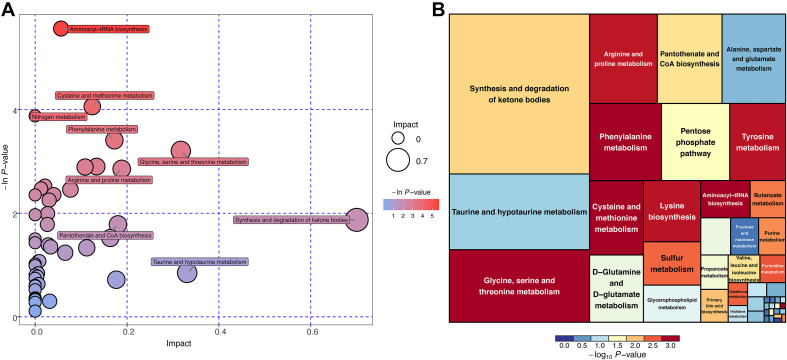
KEGG pathway enrichment analysis for differential metabolites (POS mode). **(A)** Horizontal axis and size of bubble showed topological impact of pathways. Vertical axis and color of bubble showed *p*-value of pathways. **(B)** Size of square showed topological impact of pathways, whereas color of square showed *p*-value of pathways.

Differential metabolites were subjected to the KEGG database to analyze the pathways in which these metabolites were involved. The bubble plot and tree plot demonstrated the *p*-value and topological impact of each enriched pathway (Figure 6, Supplementary Figure 4, and Supplementary Table 5).

### Adjustment for Covariates in Differential Genera and Metabolites

Most of the identified features still remain after performing adjustments for the covariates, including age and sex. First, in the differential genera after adjustments using the generalized linear model. *Anaerostipes*, *Clostridium*_XVIII, *Gemella*, and *Lactobacillus* were found to be significantly associated with sex. *Clostridium*_XlVa and *Parvimonas* were found to be significantly associated with age and sex. In the differential metabolites after adjustment using PERMANOVA, no metabolites were found to be associated with covariates (*p* > 0.05). The details of the adjustments for differential genera and metabolites could be, respectively, seen in Supplementary Tables 6, 7.

### Transcriptomic Profiling of Carotid Atherosclerosis Patients and Controls

#### Differentially Expressed Gene Screening

**FIGURE 7:**
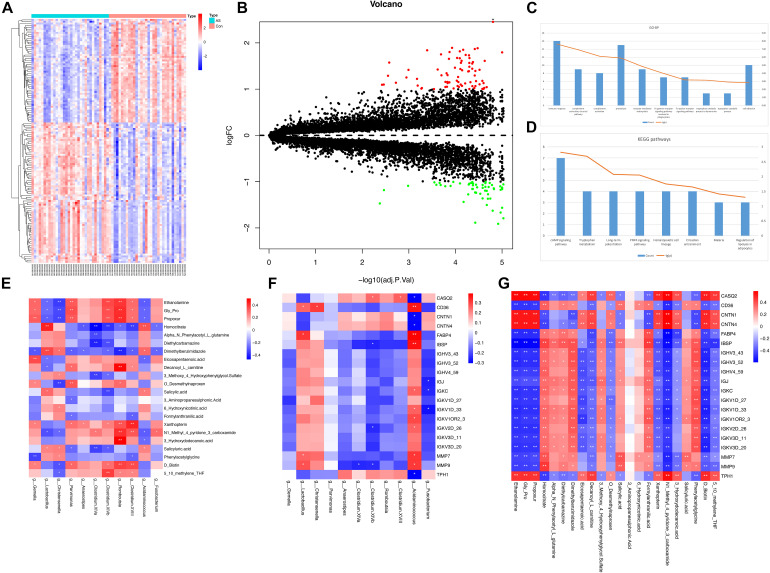
DEG analysis and correlation between different types of omics data. **(A,B)** Expression patterns of DEGs were shown by heatmap and volcano plot. **(C,D)** GO-BP terms and KEGG pathways with top-10 count number were visualized. Functional enrichment analysis for DEGs showed that these DEGs were mainly associated with inflammatory and immune response. **(E–G)** Pairwise correlation between microbiome, metabolome, and transcriptome data. Reder color indicates a stronger correlation, whereas bluer color indicates a weaker correlation.

There were 32 CAS patients in the GEO datasets we selected (GSE43292). The gene expression profiles of carotid atheroma and paired macroscopically intact tissue adjacent to the atheroma plaque of each patient were shown by mRNA microarray. To reduce the effect of confounding factors, we performed a paired DEG analysis, and a total of 132 DEGs were screened, of which 76 were upregulated and 56 were downregulated with the thresholds of | log_2_(fold-change)| > 1 and adjusted *p* < 0.01 (Figures 7A,B). DEGs with the top-20 | log_2_(fold-change)| were selected for correlation analysis (Table 4).

**TABLE 4:** DEGs with top-20 | log_2_(fold-change)|.

Gene symbol	Gene name	Log_2_(fold-change)	Adjusted *p*-value
FABP4	Fatty acid binding protein 4	2.454461	1.56E-05
CNTN1	Contactin 1	−1.911032	1.21E-05
IGJ	Joining chain of multimeric IgA and IgM	1.893149	0.000118
TPH1	Tryptophan hydroxylase 1	−1.886626	3.76E-05
IGKV1D-33	Immunoglobulin κ variable 1D-33	1.884738	3.34E-05
IGHV3-52	Immunoglobulin heavy variable 3-52	1.8676	3.85E-05
IGKV3D-11	Immunoglobulin κ variable 3D-11	1.851044	6.82E-05
MMP7	Matrix metallopeptidase 7	1.840231	0.000405
MMP9	Matrix metallopeptidase 9	1.817804	8.07E-05
CD36	CD36 molecule	1.802205	0.000134
IBSP	Integrin binding sialoprotein	1.794982	9.96E-06
CNTN4	Contactin 4	−1.792332	9.36E-06
IGKV1D-27	Immunoglobulin κ variable 1D-27	1.751979	0.000204
IGHV4-59	Immunoglobulin heavy variable 4-59	1.739775	6.03E-05
IGHV3-43	Immunoglobulin heavy variable 3-43	1.73119	5.12E-05
IGKV1OR2-3	Immunoglobulin κ variable 1/OR2-3	1.674665	9.21E-05
IGKC	Immunoglobulin κ constant	1.672012	5.01E-05
CASQ2	Calsequestrin 2	−1.667664	1.07E-05
IGKV3D-20	Immunoglobulin κ variable 3D-20	1.667174	8.07E-05
IGKV2D-26	Immunoglobulin κ variable 2D-26	1.654989	0.000138

#### Functional Annotation Analysis for Differentially Expressed Genes

To obtain the biological functions of the DEGs, GO-BP and KEGG pathway analyses were performed. Count number > 2 and *p*-value < 0.05 were selected as the thresholds for significantly enriched GO-BP terms and KEGG pathways. We have also performed enrichment analysis on the DEGs using the Reactome database. These DEGs were mainly associated with inflammatory and immune responses, as both KEGG and Reactome pathways enrichment analyses have shown (Figures 7C,D and Supplementary Tables 8, 9).

### Integration of Multi-Omics Data

Spearman’s correlation test was conducted between differentially enriched genera, differential metabolites, and DEGs to investigate the associations among multi-omics results (Supplementary Table 10). The results were visualized as correlation heatmaps (Figures 7E–G). The correlation analysis was also adjusted by using FDR (Supplementary Figure 5 and Supplementary Table 10).

**FIGURE 8:**
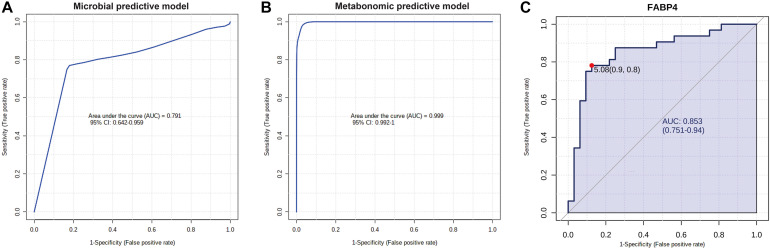
Potential diagnostic value of differential gut microbiota, metabolites, and DEG with importance. **(A)** Microbial predictive model. Gut microbiota significantly enriched in CAS group were included, and RF algorithm was used to construct this model. **(B)** Metabolomic predictive model. Phenylacetylglutamine, phenylacetylglycine, ethanolamine, and eicosapentaenoic acid were included, and RF algorithm was used to construct this model. **(C)** FABP4 is the DEG with the highest log_2_(fold-change), and potential diagnostic value of this gene was shown by ROC analysis.

Finally, to show the potential diagnostic value of multi-omics data to discriminate CAS patients and healthy controls, we performed ROC and RF analyses for differentially enriched genera, differential metabolites, and DEGs (Figure 8 and Supplementary Table 11).

## Discussion

In this multi-omics study, gut microbiota and metabolite data were obtained from samples of CAS patients and healthy controls from PUMCH, and mRNA microarray data were obtained from GSE43292, which includes 32 atheromas and 32 paired control samples. The microbiome study showed significantly different β-diversity between CAS patients and healthy controls, although the α-diversity between the two groups was not significantly different, suggesting a significant difference in microbial composition, although there were similarities in microbial richness. At the genera level, 11 differentially enriched microbiota were identified (Table 2). In these differentially enriched microbiota, *Acidaminococcus*, *Christensenella*, and *Lactobacillus* genera were enriched in CAS patients. The metabolome analysis screened 165 and 96 differentially expressed metabolites in the POS and NEG modes, respectively. Next, 22 differential metabolites were further selected for correlation analysis by adding | log2(fold-change)| > 1 as an additional threshold (Table 3). In transcriptomic analysis, 76 upregulated and 56 downregulated genes were screened, and DEGs with top-20 | log_2_(fold-change)| were included for correlation analysis (Table 4). Spearman’s correlation indices showed the association among different omics results.

In the genus-level analysis of differential gut microbiota, *Acidaminococcus*, *Christensenella*, and *Lactobacillus* were more abundant in the CAS group, whereas *Anaerostipes*, *Clostridium* XVIII/XlVa/XlVb, *Fusobacterium*, *Gemella*, *Parvimonas*, and *Romboutsia* were enriched in the healthy controls. *Acidaminococcus* is known to be a normal commensal of the human gut and has been occasionally related to infective processes but always associated with polymicrobial infections (D’Auria et al., 2011). *Acidaminococcus* was reported to be enriched in the stool of patients with several inflammatory diseases, such as rheumatoid arthritis, ankylosing spondylitis, and ulcerative colitis (Altomare et al., 2019; Lee J. Y. et al., 2019; Zhou et al., 2020). Moreover, according to a recent study by Zheng et al. (2020), the abundance of *Acidaminococcus* is positively correlated with a pro-inflammatory diet, indicating that *Acidaminococcus* may be a pro-inflammatory microbiota and represent inflammatory status in the development of AS. *Christensenella* is a gram-negative, strictly anaerobic short rod associated with weight loss (Morotomi et al., 2012). Several studies have indicated that *Christensenella* was enriched in type 1 diabetes patients with the decreased abundance of the SCFA-producing microbiota, Roseburia. In addition, whole-genome sequencing indicated that some genes of *Christensenella* were related to lipopolysaccharide biosynthesis, and the lipopolysaccharide from *Christensenella* can trigger a weak inflammatory response through the nuclear factor kappa-B (NF-κB) signaling pathway (Yang et al., 2018). Although *Lactobacillus* is often described as an anti-inflammatory probiotic in many studies of AS, the role of *Lactobacillus* in the pathogenesis of AS remains controversial (Ding et al., 2017). Several *Lactobacillus* species significantly reduce the inflammatory response *via* T regulatory cells and alleviate arteriosclerotic level, but some other species of *Lactobacillus* could promote the inflammatory response, which may aggravate AS (Smits et al., 2005; Bhathena et al., 2009; Karimi et al., 2009; Pan et al., 2011; Won et al., 2011; Shah et al., 2012; Dimitrijevic et al., 2014). The increased abundance of *Lactobacillus* in the CAS group may fall into different species; therefore, additional research is needed to address this question.

For the genera enriched in healthy controls, *Anaerostipes*, *Gemella*, and *Parvimonas* were reported to be scarce and primarily enriched in the healthy gut (Bodkhe et al., 2019; Hong et al., 2019; Magruder et al., 2020). *Clostridium* XVIII/XlVa/XlVb, *Fusobacterium*, and *Romboutsia* are all major SCFAs, particularly butyrate producers in the process of human metabolism (Duncan et al., 2002; Bui et al., 2014; Neijat et al., 2019). In humans, SCFAs are produced from dietary fibers and resistant starches that cannot be decomposed by digestive enzymes through fermentation by the microbiota in the cecum and colon (Cummings et al., 1987). SCFAs may suppress inflammation by reducing migration and proliferation of immune cells, thereby reducing many types of cytokines and inducing apoptosis (Ohira et al., 2017). Furthermore, data from animal experiments found that compared with the sterile mice, the atherosclerotic plaque of mice carrying *Roseburia* was significantly reduced after they were fed with a high fiber diet. The mechanism was that SCFAs could inhibit the activation of histone deacetylase, NF-κB, and tumor necrosis factor-α signaling pathways, reduce the expression of vascular cell adhesion molecule-1, and protect endothelial function. On the other hand, SCFAs could promote the conversion of cholesterol to bile acid, thereby alleviating AS (Kasahara et al., 2018).

Based on the metabolomic analysis, the upregulated PAGln was detected in both POS and NEG modes and had the highest | log_2_(fold-change)| in POS mode. PAGln is a phenylalanine-derived metabolite formed from the conjugation of glutamine and phenylacetate (Aronov et al., 2011). Phenylalanine is one of the essential amino acids for human metabolism. After phenylalanine is ingested by the human body, most of this amino acid is absorbed by the small intestine. Excessive phenylalanine reaches the colon and can be metabolized into phenylpyruvic acid further into phenylacetic acid by gut microbiota. Next, glutamine and this microbial-derived phenylacetic acid are conjugated in the human liver and kidney, and PAGln is produced (Li et al., 2008; Witkowski et al., 2020). Increased level of plasma PAGln was shown to be associated with increased major adverse cardiac events (myocardial infarction, stroke, or death) by untargeted metabolomics using a large cohort (*n* = 1,162) and a validation cohort (*n* = 4,000) (Nemet et al., 2020). Bogiatzi et al. (2018) also discovered that PAGln was elevated in AS patients. Our results were consistent with the findings of these previous studies. In addition, the KEGG pathway analysis in our study found that differential metabolites were significantly enriched in phenylalanine metabolic pathway for both POS and NEG modes, suggesting that phenylalanine metabolism and subsequently generated PAGln play vital roles in CAS pathogenesis. A recent mechanistic study has shown that PAGln increases thrombosis potential by activating platelet functions through multiple approaches such as interacting with α2A, α2B, and β2 adrenergic receptors (Nemet et al., 2020). Another upregulated metabolite in the NEG mode, phenylacetylglycine (PAGly), has a similar function as PAGln and can enhance platelet function *via* adrenergic receptors. However, compared with PAGln, PAGly was a major product in mice found in the study of Nemet et al. (2020). Our study and previous findings indicated that PAGln and phenylalanine metabolism are crucial mediators in CAS pathogenesis and might serve as promising pharmacotherapeutic targets to slow CAS progression.

Ethanolamine was the differential metabolite with the highest VIP value in POS mode and downregulated in CAS patients. The level of ethanolamine in HDL is positively correlated with cholesterol efflux capacity and negatively associated with plaque scores in chronic kidney disease (CKD) patients (Maeba et al., 2018). The finding of downregulated ethanolamine in CAS patients in our study was consistent with this previous study and suggested that downregulation of ethanolamine might promote AS progression. In contrast to this previous study, the patients in our study were not CKD patients and might be more representative. Another downregulated metabolite, eicosapentaenoic acid (EPA), is an omega-3 fatty acid found in fish oil. EPA and its derivatives were found to have protective roles against cardiovascular disease in clinical trials (Leaf et al., 1994; Sacks et al., 1995; Bhatt et al., 2020). EPA can be enzymatically converted to resolvin E1 (RvE1) *in vivo* and affect atherosclerotic inflammation and mediate the immune response through the EPA/RvE1/ChemR23 pathway, thereby improving the outcomes of atherosclerosis-related cardiovascular disease (Carracedo et al., 2019). Our results indicated a deficiency of these beneficial metabolites in CAS patients, and supplementation with fish oil might benefit these patients.

In transcriptomic analysis, we observed that DEGs were mainly associated with inflammatory and immune response through GO-BP and KEGG pathway enrichment analysis. Our findings at the transcriptome level agree with the consensus that atherosclerosis is characterized by low-grade, chronic inflammation of the arteries and infiltration of immune cells such as macrophages, mast cells, and T lymphocytes (Hansson, 2005; Galkina and Ley, 2009; Bäck et al., 2019). Furthermore, *FABP4*, fatty acid-binding protein 4, is the upregulated DEG in CAS patients with the highest | log_2_(fold-change)|. FABP4 is mainly expressed in adipocytes and macrophages. This protein can serve as an adipokine for the development of atherosclerosis and insulin resistance (Hotamisligil and Bernlohr, 2015). In macrophages, FABP4 can induce an inflammatory response through such pathways as NF-κB and JKN/AP-1 (Furuhashi, 2019).

In the correlation analysis between gut microbiota and plasma metabolites, PAGln was negatively associated with *Clostridium* XIVa, which belongs to the Lachnospiraceae family. In early renal function decline patients, Barrios et al. (2015) found that PAGln was negatively correlated with several genera in the Lachnospiraceae family, and in patients with coronary artery disease, Ottosson et al. (2020) identified one unknown genus in the Lachnospiraceae family that was also negatively correlated with PAGln. The results of our study were consistent with the findings of previous studies and further identified a new genus in this family that was negatively correlated with PAGln in CAS patients. Although few studies on this topic have been conducted, the association between Lachnospiraceae and PAGln might be one of the microbiota–metabolite axes mediating AS pathogenesis. EPA, the metabolite with anti-inflammatory roles in AS patients, was negatively associated with *Acidaminococcus*, a potentially pro-inflammatory microbiota genus. Because a Mediterranean diet, which mainly includes foods rich in unsaturated fatty acid (such as EPA), can have an anti-inflammatory effect (Zheng et al., 2020), we could infer that EPA might reduce atherosclerotic inflammation by targeting *Acidaminococcus*.

In the correlation analysis between transcriptomic profiles and the other two omics datasets, we also obtained some findings that might deepen the current understanding of AS pathogenesis. We found that *Acidaminococcus* was positively associated with *FABP4* and had the highest Spearman’s correlation coefficient and the most significant *p*-value among all microbiota–DEG pairs (ρ = 0.39, *p* = 0.0014). Although the pro-inflammatory roles of *Acidaminococcus* and *FABP4* have been widely studied (Hotamisligil and Bernlohr, 2015; Altomare et al., 2019; Butera et al., 2020), our study was the first to identify the association between them and might provide a new perspective to explore CAS pathogenesis. Furthermore, in the correlation analysis for DEGs and metabolites, *FABP4* was also positively associated with pro-atherosclerotic metabolites (PAGln and PAGly) and negatively associated with anti-atherosclerotic metabolite (ethanolamine), which implied that this adipokine was not only associated with crucial gut microbiota but also might interact with crucial metabolites in CAS pathogenesis.

In this study, we performed microbial and metabolomic analyses using fecal and plasma samples from CAS patients in PUMCH. Transcriptomic analysis was conducted based on one GEO dataset (GSE43292) containing 32 CAS carotid atheromas and paired controls. Differential gut microbiota, metabolites, DEGs, and related pathways were identified. Finally, the associations among various omics data were investigated by correlation analysis. However, our study has some limitations. First, the body mass index was marginally higher (24.7 ± 2.7 for CAS patients and 23.2 ± 2.2 for healthy controls, *p* = 0.047) in CAS patients; the risk factor role of obesity might account for this difference (Rocha and Libby, 2009). Second, patients were only recruited from PUMCH, and the sample size was small. Future multicentric studies with large samples are needed to generalize these findings. Third, transcriptomic data obtained from the GEO database were not obtained from the same patients as the microbiome and metabolome data. This difference would result in batch effects, which need to be verified by the same cohorts. Furthermore, *in vitro* and *in vivo* experiments are warranted to elucidate further the mechanisms governing how gut microbiota, plasma metabolites, and DEGs interact with one another.

## Conclusion

Despite extensive researches investigating AS, in the past decade, relatively little is known regarding the mechanisms underlying the pathogenesis of CAS. Accumulating evidence has shown that the gut microbiota serve as a pivotal risk factor in cardiovascular diseases by influencing host metabolism and immune homeostasis (Battson et al., 2018). However, no direct evidence has established a direct and causal relationship between altered gut microbiota and CAS. Through an integrated analysis of multi-omics, we explored the possible “microbiota–metabolite–gene” regulatory axis that may act on CAS, thereby helping to establish a theoretical basis for the further specialized study of CAS.

## Data Availability Statement

The datasets presented in this study can be found in online repositories. The names of the repository/repositories and accession number(s) can be found below: National Center for Biotechnology Information (NCBI) Gene Expression Omnibus (GEO), https://www.ncbi.nlm.nih.gov/geo/, GSE28829, GSE104140, and GSE43292. The study contains 16S rDNA sequencing data and we have uploaded the original fastq data in the Sequence Read Archive (SRA) database (BioProject: PRJNA674452, https://www.ncbi.nlm.nih.gov/sra/PRJNA674452).

## Ethics Statement

The studies involving human participants were reviewed and approved by Ethics Committee of the Peking Union Medical College Hospital. The patients/participants provided their written informed consent to participate in this study.

## Author Contributions

LJ and SC conceived and designed the experiments, performed the experiments, analyzed the data, contributed reagents, materials, and analysis tools, prepared figures and/or tables, authored or reviewed drafts of the manuscript, and approved the final draft. GG conceived and designed the experiments, performed the experiments, analyzed the data, authored or reviewed drafts of the manuscript, and approved the final draft. JZ, JR, and JW analyzed the data, prepared figures and/or tables, and approved the final draft. WW and DY analyzed the data, authored or reviewed drafts of the manuscript, and approved the final draft. YZ conceived and designed the experiments, authored or reviewed drafts of the manuscript, uploaded the original fastq data of 16S rDNA sequencing in the Sequence Read Archive (SRA) database and approved the final draft. All authors contributed to the article and approved the submitted version.

## Conflict of Interest

The authors declare that the research was conducted in the absence of any commercial or financial relationships that could be construed as a potential conflict of interest.

## Footnotes

1 https://david.ncifcrf.gov/

2 https://reactome.org/

3 http://rdp.cme.msu.edu/

## Supplementary Material

Supplementary Figure 1 Quality control for 16S rDNA sequencing and microbial diversity of CAS and healthy control samples from different level. **(A,B)** Rarefaction curves of richness (observed_species and chao 1) suggested the sequencing depth is adequate. **(C)** The Venn diagram showed the overlapping OTUs in microbiota among CAS patients and healthy controls. **(D–G)** The relative abundance of gut microbiota for two groups from phylum, class, order, and family levels, respectively.

Supplementary Figure 2 Differentially enriched microbiota for CAS patients and healthy controls from all levels. **(A)** The differentially enriched gut microbiotas from all levels were visualized in heatmap. **(B)** PCA plot demonstrated that CAS group is significantly different from control group based on differential microbiotas from all levels. **(C)** The differentially enriched gut microbiotas were visualized in box plot.

Supplementary Figure 3 Differential metabolites screening for CAS patients and healthy controls (NEG mode) and permutation test for OPLS-DA model. **(A,B)** The PCA and OPLS-DA showed that CAS patients and healthy controls can be clearly separated. **(C,D)** The permutation test showed that the OPLS-DA model is valid and no overfitting exist for both POS and NEG mode. **(E)** The Venn diagram showed 17 overlapping different metabolites between POS and NEG mode. **(F,G)** Patterns of differential metabolites in NEG mode were demonstrated by the volcano plot and heatmap.

Supplementary Figure 4 KEGG pathway enrichment analysis for differential metabolites (NEG mode). **(A)** The horizontal axis and sized of the bubble showed the topological impact of pathways. The vertical axis and color of bubble showed the *p*-value of pathways. **(B)** The size of the square showed the topological impact of pathways while the color of the square showed the *p*-value of the pathways.

Supplementary Figure 5 FDR adjustment for correlation between different types of omics data. **(A–C)** The FDR adjustment for correlation between microbiome, metabolome, and transcriptome data. Redder color indicates stronger correlation while bluer color indicates weaker correlation.

Supplementary Table 1 The α-diversity for CAS and healthy controls.

Supplementary Table 2 Differentially enriched microbiota from all levels.

Supplementary Table 3 Differential KEGG pathways predicted by PICRUSt.

Supplementary Table 4 All differential metabolites between CAS patients and healthy controls.

Supplementary Table 5 KEGG pathways for differential metabolites.

Supplementary Table 6 Covariates adjustment for differentially enriched genera between CAS patients and healthy controls using GLM analysis.

Supplementary Table 7 Covariates adjustment for differential metabolites between CAS patients and healthy controls using PERMANOVA analysis.

Supplementary Table 8 Functional enrichment analysis for DEGs.

Supplementary Table 9 Enrichment analysis on the DEGs using the Reactome database.

Supplementary Table 10 Details for correlation analysis.

Supplementary Table 11 AUCs for differentially enriched gut microbiota, metabolites, and DEGs.

## Abbreviations

AS: atherosclerosis
CAS: carotid atherosclerosis
CKD: chronic kidney disease
DEG: differentially expressed gene
EPA: eicosapentaenoic acid
GEO: Gene Expression Omnibus
GO-BP: gene ontology-biological process
KEGG: Kyoto Encyclopedia of Genes and Genomes
LDA: linear discriminant analysis
LEfSe: linear discriminant analysis effect size
NEG: negative mode
NF- κ B: nuclear factor kappa-B
OTU: operational taxonomic unit
PAGly: phenylacetylglycine
PCA: principal component analysis
PERMANOVA: permutational multivariate analysis of variance
PICRUSt: phylogenetic investigation of communities by reconstruction of unobserved states
POS: positive mode
QIIME: quantitative insights into microbial ecology
RF: random forest
ROC: receiver operating characteristic
SCFA: short-chain fatty acid
UHPLC-QTOFMS: ultra-high-performance liquid tandem chromatography/quadrupole time-of-flight mass spectrometry.

## References

[B1] Aboyans V. Ricco J. B. Bartelink M. E. L. Bjorck M. Brodmann M. Cohnert T. (2018). 2017 ESC Guidelines on the Diagnosis and Treatment of Peripheral Arterial Diseases, in collaboration with the European Society for Vascular Surgery (ESVS): Document covering atherosclerotic disease of extracranial carotid and vertebral, mesenteric, renal, upper and lower extremity arteriesEndorsed by: the European Stroke Organization (ESO)The Task Force for the Diagnosis and Treatment of Peripheral Arterial Diseases of the European Society of Cardiology (ESC) and of the European Society for Vascular Surgery (ESVS). *Eur Heart J* 39 763–816. 10.1093/eurheartj/ehx095 28886620

[B2] Altomare A. Putignani L. Del Chierico F. Cocca S. Angeletti S. Ciccozzi M. (2019). Gut mucosal-associated microbiota better discloses inflammatory bowel disease differential patterns than faecal microbiota. *Dig Liver Dis* 51 648–656. 10.1016/j.dld.2018.11.021 30573380

[B3] Aronov P. A. Luo F. J. Plummer N. S. Quan Z. Holmes S. Hostetter T. H. (2011). Colonic contribution to uremic solutes. *J Am Soc Nephrol* 22 1769–1776. 10.1681/asn.2010121220 21784895 PMC3171947

[B4] Artom N. Montecucco F. Dallegri F. Pende A. (2014). Carotid atherosclerotic plaque stenosis: the stabilizing role of statins. *Eur J Clin Invest* 44 1122–1134. 10.1111/eci.12340 25231921

[B5] Bäck M. Yurdagul A. Jr. Tabas I. Öörni K. Kovanen P. T. (2019). Inflammation and its resolution in atherosclerosis: mediators and therapeutic opportunities. *Nat Rev Cardiol* 16 389–406. 10.1038/s41569-019-0169-2 30846875 PMC6727648

[B6] Barrios C. Beaumont M. Pallister T. Villar J. Goodrich J. K. Clark A. (2015). Gut-Microbiota-Metabolite Axis in Early Renal Function Decline. *PLoS One* 10:e0134311. 10.1371/journal.pone.0134311 26241311 PMC4524635

[B7] Battson M. L. Lee D. M. Weir T. L. Gentile C. L. (2018). The gut microbiota as a novel regulator of cardiovascular function and disease. *J Nutr Biochem* 56 1–15. 10.1016/j.jnutbio.2017.12.010 29427903

[B8] Bhathena J. Martoni C. Kulamarva A. Urbanska A. M. Malhotra M. Prakash S. (2009). Orally delivered microencapsulated live probiotic formulation lowers serum lipids in hypercholesterolemic hamsters. *J Med Food* 12 310–319. 10.1089/jmf.2008.0166 19459731

[B9] Bhatt D. L. Miller M. Brinton E. A. Jacobson T. A. Steg P. G. Ketchum S. B. (2020). REDUCE-IT USA: Results From the 3146 Patients Randomized in the United States. *Circulation* 141 367–375. 10.1161/circulationaha.119.044440 31707829 PMC7004453

[B10] Bodkhe R. Shetty S. A. Dhotre D. P. Verma A. K. Bhatia K. Mishra A. (2019). Comparison of Small Gut and Whole Gut Microbiota of First-Degree Relatives With Adult Celiac Disease Patients and Controls. *Front Microbiol* 10:164. 10.3389/fmicb.2019.00164 30800106 PMC6376745

[B11] Bogiatzi C. Gloor G. Allen-Vercoe E. Reid G. Wong R. G. Urquhart B. L. (2018). Metabolic products of the intestinal microbiome and extremes of atherosclerosis. *Atherosclerosis* 273 91–97. 10.1016/j.atherosclerosis.2018.04.015 29702430

[B12] Bui T. P. N. de Vos W. M. Plugge C. M. (2014). Anaerostipes rhamnosivorans sp. nov., a human intestinal, butyrate-forming bacterium. *Int J Syst Evol Microbiol* 64(Pt 3), 787–793. 10.1099/ijs.0.055061-0 24215821

[B13] Butera A. Di Paola M. Vitali F. De Nitto D. Covotta F. Borrini F. (2020). IL-13 mRNA Tissue Content Identifies Two Subsets of Adult Ulcerative Colitis Patients With Different Clinical and Mucosa-Associated Microbiota Profiles. *J Crohns Colitis* 14 369–380. 10.1093/ecco-jcc/jjz154 31501882

[B14] Caporaso J. G. Kuczynski J. Stombaugh J. Bittinger K. Bushman F. D. Costello E. K. (2010). QIIME allows analysis of high-throughput community sequencing data. *Nat Methods* 7 335–336. 10.1038/nmeth.f.303 20383131 PMC3156573

[B15] Carracedo M. Artiach G. Arnardottir H. Bäck M. (2019). The resolution of inflammation through omega-3 fatty acids in atherosclerosis, intimal hyperplasia, and vascular calcification. *Semin Immunopathol* 41 757–766. 10.1007/s00281-019-00767-y 31696250 PMC6881483

[B16] Chen Y. Xu C. Huang R. Song J. Li D. Xia M. (2018). Butyrate from pectin fermentation inhibits intestinal cholesterol absorption and attenuates atherosclerosis in apolipoprotein E-deficient mice. *J Nutr Biochem* 56 175–182. 10.1016/j.jnutbio.2018.02.011 29574331

[B17] Cummings J. H. Pomare E. W. Branch W. J. Naylor C. P. Macfarlane G. T. (1987). Short chain fatty acids in human large intestine, portal, hepatic and venous blood. *Gut* 28 1221–1227. 10.1136/gut.28.10.1221 3678950 PMC1433442

[B18] D’Auria G. Galan J. C. Rodriguez-Alcayna M. Moya A. Baquero F. Latorre A. (2011). Complete genome sequence of Acidaminococcus intestini RYC-MR95, a Gram-negative bacterium from the phylum Firmicutes. *J Bacteriol* 193 7008–7009. 10.1128/JB.06301-11 22123762 PMC3232847

[B19] Dimitrijevic R. Ivanovic N. Mathiesen G. Petrusic V. Zivkovic I. Djordjevic B. (2014). Effects of Lactobacillus rhamnosus LA68 on the immune system of C57BL/6 mice upon oral administration. *J Dairy Res* 81 202–207. 10.1017/S0022029914000028 24559976

[B20] Ding Y. H. Qian L. Y. Pang J. Lin J. Y. Xu Q. Wang L. H. (2017). The regulation of immune cells by Lactobacilli: a potential therapeutic target for anti-atherosclerosis therapy. *Oncotarget* 8 59915–59928. 10.18632/oncotarget.18346 28938693 PMC5601789

[B21] Duncan S. H. Hold G. L. Harmsen H. J. M. Stewart C. S. Flint H. J. (2002). Growth requirements and fermentation products of Fusobacterium prausnitzii, and a proposal to reclassify it as Faecalibacterium prausnitzii gen. nov., comb. nov. *Int J Syst Evol Microbiol* 52(Pt 6), 2141–2146. 10.1099/00207713-52-6-2141 12508881

[B22] Dunn W. B. Broadhurst D. Begley P. Zelena E. Francis-McIntyre S. Anderson N. (2011). Procedures for large-scale metabolic profiling of serum and plasma using gas chromatography and liquid chromatography coupled to mass spectrometry. *Nat Protoc* 6 1060–1083. 10.1038/nprot.2011.335 21720319

[B23] Edgar R. Domrachev M. Lash A. E. (2002). Gene Expression Omnibus: NCBI gene expression and hybridization array data repository. *Nucleic Acids Res* 30 207–210. 10.1093/nar/30.1.207 11752295 PMC99122

[B24] Edgar R. C. (2013). UPARSE: highly accurate OTU sequences from microbial amplicon reads. *Nat Methods* 10 996–998. 10.1038/nmeth.2604 23955772

[B25] Faxon D. P. Fuster V. Libby P. Beckman J. A. Hiatt W. R. Thompson R. W. (2004). Atherosclerotic Vascular Disease Conference: Writing Group III: pathophysiology. *Circulation* 109 2617–2625. 10.1161/01.CIR.0000128520.37674.EF 15173044

[B26] Furuhashi M. (2019). Fatty Acid-Binding Protein 4 in Cardiovascular and Metabolic Diseases. *J Atheroscler Thromb* 26 216–232. 10.5551/jat.48710 30726793 PMC6402888

[B27] Galkina E. Ley K. (2009). Immune and inflammatory mechanisms of atherosclerosis (^∗^). *Annu Rev Immunol* 27 165–197. 10.1146/annurev.immunol.021908.132620 19302038 PMC2734407

[B28] Hansson G. K. (2005). Inflammation, atherosclerosis, and coronary artery disease. *N Engl J Med* 352 1685–1695. 10.1056/NEJMra043430 15843671

[B29] Hong B. Y. Sobue T. Choquette L. Dupuy A. K. Thompson A. Burleson J. A. (2019). Chemotherapy-induced oral mucositis is associated with detrimental bacterial dysbiosis. *Microbiome* 7 66. 10.1186/s40168-019-0679-5 31018870 PMC6482518

[B30] Hotamisligil G. S. Bernlohr D. A. (2015). Metabolic functions of FABPs–mechanisms and therapeutic implications. *Nat Rev Endocrinol* 11 592–605. 10.1038/nrendo.2015.122 26260145 PMC4578711

[B31] Jonsson A. L. Backhed F. (2017). Role of gut microbiota in atherosclerosis. *Nat Rev Cardiol* 14 79–87. 10.1038/nrcardio.2016.183 27905479

[B32] Karimi K. Inman M. D. Bienenstock J. Forsythe P. (2009). Lactobacillus reuteri-induced regulatory T cells protect against an allergic airway response in mice. *Am J Respir Crit Care Med* 179 186–193. 10.1164/rccm.200806-951OC 19029003

[B33] Kasahara K. Krautkramer K. A. Org E. Romano K. A. Kerby R. L. Vivas E. I. (2018). Interactions between Roseburia intestinalis and diet modulate atherogenesis in a murine model. *Nat Microbiol* 3 1461–1471. 10.1038/s41564-018-0272-x 30397344 PMC6280189

[B34] Koren O. Spor A. Felin J. Fak F. Stombaugh J. Tremaroli V. (2011). Human oral, gut, and plaque microbiota in patients with atherosclerosis. *Proc Natl Acad Sci U S A* 108(Suppl. 1), 4592–4598. 10.1073/pnas.1011383107 20937873 PMC3063583

[B35] Langille M. G. Zaneveld J. Caporaso J. G. McDonald D. Knights D. Reyes J. A. (2013). Predictive functional profiling of microbial communities using 16S rRNA marker gene sequences. *Nat Biotechnol* 31 814–821. 10.1038/nbt.2676 23975157 PMC3819121

[B36] Leaf A. Jorgensen M. B. Jacobs A. K. Cote G. Schoenfeld D. A. Scheer J. (1994). Do fish oils prevent restenosis after coronary angioplasty? *Circulation* 90 2248–2257. 10.1161/01.cir.90.5.2248 7955181

[B37] Lee J. Y. Mannaa M. Kim Y. Kim J. Kim G. T. Seo Y. S. (2019). Comparative Analysis of Fecal Microbiota Composition Between Rheumatoid Arthritis and Osteoarthritis Patients. *Genes (Basel)* 10 10.3390/genes10100748 31557878 PMC6827100

[B38] Lee T. H. Cheng M. L. Shiao M. S. Lin C. N. (2019). Metabolomics study in severe extracranial carotid artery stenosis. *BMC Neurol* 19:138. 10.1186/s12883-019-1371-x 31234801 PMC6589885

[B39] Li M. Wang B. Zhang M. Rantalainen M. Wang S. Zhou H. (2008). Symbiotic gut microbes modulate human metabolic phenotypes. *Proc Natl Acad Sci U S A* 105 2117–2122. 10.1073/pnas.0712038105 18252821 PMC2538887

[B40] Libby P. Buring J. E. Badimon L. Hansson G. K. Deanfield J. Bittencourt M. S. (2019). Atherosclerosis. *Nat Rev Dis Primers* 5 56. 10.1038/s41572-019-0106-z 31420554

[B41] Lindskog Jonsson A. Hallenius F. F. Akrami R. Johansson E. Wester P. Arnerlov C. (2017). Bacterial profile in human atherosclerotic plaques. *Atherosclerosis* 263 177–183. 10.1016/j.atherosclerosis.2017.06.016 28646792

[B42] Maeba R. Kojima K. I. Nagura M. Komori A. Nishimukai M. Okazaki T. (2018). Association of cholesterol efflux capacity with plasmalogen levels of high-density lipoprotein: A cross-sectional study in chronic kidney disease patients. *Atherosclerosis* 270 102–109. 10.1016/j.atherosclerosis.2018.01.037 29407877

[B43] Magruder M. Edusei E. Zhang L. Albakry S. Satlin M. J. Westblade L. F. (2020). Gut commensal microbiota and decreased risk for *Enterobacteriaceae* bacteriuria and urinary tract infection. *Gut Microbes* 12 1805281. 10.1080/19490976.2020.1805281 32865119 PMC7524266

[B44] Morotomi M. Nagai F. Watanabe Y. (2012). Description of Christensenella minuta gen. nov., sp. nov., isolated from human faeces, which forms a distinct branch in the order Clostridiales, and proposal of Christensenellaceae fam. nov. *Int J Syst Evol Microbiol* 62(Pt 1), 144–149. 10.1099/ijs.0.026989-0 21357455

[B45] Neijat M. Habtewold J. Shirley R. B. Welsher A. Barton J. Thiery P. (2019). Bacillus subtilis Strain DSM 29784 Modulates the Cecal Microbiome, Concentration of Short-Chain Fatty Acids, and Apparent Retention of Dietary Components in Shaver White Chickens during Grower, Developer, and Laying Phases. *Appl Environ Microbiol* 85 10.1128/AEM.00402-19 31076425 PMC6606875

[B46] Nemet I. Saha P. P. Gupta N. Zhu W. Romano K. A. Skye S. M. (2020). A Cardiovascular Disease-Linked Gut Microbial Metabolite Acts via Adrenergic Receptors. *Cell* 180 862.e–877.e. 10.1016/j.cell.2020.02.016 32142679 PMC7402401

[B47] Ohira H. Tsutsui W. Fujioka Y. (2017). Are Short Chain Fatty Acids in Gut Microbiota Defensive Players for Inflammation and Atherosclerosis? *J Atheroscler Thromb* 24 660–672. 10.5551/jat.RV17006 28552897 PMC5517538

[B48] Ottosson F. Brunkwall L. Smith E. Orho-Melander M. Nilsson P. M. Fernandez C. (2020). The gut microbiota-related metabolite phenylacetylglutamine associates with increased risk of incident coronary artery disease. *J Hypertens* 10.1097/hjh.0000000000002569 32665522

[B49] Pan D. D. Zeng X. Q. Yan Y. T. (2011). Characterisation of Lactobacillus fermentum SM-7 isolated from koumiss, a potential probiotic bacterium with cholesterol-lowering effects. *J Sci Food Agric* 91 512–518. 10.1002/jsfa.4214 21218486

[B50] Petty G. W. Brown R. D. Jr. Whisnant J. P. Sicks J. D. O’Fallon W. M. Wiebers D. O. (2000). Ischemic stroke subtypes : a population-based study of functional outcome, survival, and recurrence. *Stroke* 31 1062–1068. 10.1161/01.str.31.5.1062 10797166

[B51] Qian Y. Yang X. Xu S. Wu C. Song Y. Qin N. (2018). Alteration of the fecal microbiota in chinese patients with parkinson’s disease. *Brain Behavior and Immunity* 70 194–202. 10.1016/j.bbi.2018.02.016 29501802

[B52] Rocha V. Z. Libby P. (2009). Obesity, inflammation, and atherosclerosis. *Nat Rev Cardiol* 6 399–409. 10.1038/nrcardio.2009.55 19399028

[B53] Sacks F. M. Stone P. H. Gibson C. M. Silverman D. I. Rosner B. Pasternak R. C. (1995). Controlled trial of fish oil for regression of human coronary atherosclerosis. HARP Research Group. *J Am Coll Cardiol* 25 1492–1498. 10.1016/0735-1097(95)00095-l 7759696

[B54] Segata N. Izard J. Waldron L. Gevers D. Miropolsky L. Garrett W. S. (2011). Metagenomic biomarker discovery and explanation. *Genome Biol* 12 R60. 10.1186/gb-2011-12-6-r60 21702898 PMC3218848

[B55] Shah M. M. Saio M. Yamashita H. Tanaka H. Takami T. Ezaki T. (2012). Lactobacillus acidophilus strain L-92 induces CD4(+)CD25(+)Foxp3(+) regulatory T cells and suppresses allergic contact dermatitis. *Biol Pharm Bull* 35 612–616. 10.1248/bpb.35.612 22466569

[B56] Shannon P. Markiel A. Ozier O. Baliga N. S. Wang J. T. Ramage D. (2003). Cytoscape: a software environment for integrated models of biomolecular interaction networks. *Genome Res* 13 2498–2504. 10.1101/gr.1239303 14597658 PMC403769

[B57] Smits H. H. Engering A. van der Kleij D. de Jong E. C. Schipper K. van Capel T. M. (2005). Selective probiotic bacteria induce IL-10-producing regulatory T cells in vitro by modulating dendritic cell function through dendritic cell-specific intercellular adhesion molecule 3-grabbing nonintegrin. *J Allergy Clin Immunol* 115 1260–1267. 10.1016/j.jaci.2005.03.036 15940144

[B58] Song P. Fang Z. Wang H. Cai Y. Rahimi K. Zhu Y. (2020). Global and regional prevalence, burden, and risk factors for carotid atherosclerosis: a systematic review, meta-analysis, and modelling study. *Lancet Glob Health* 8 e721–e729. 10.1016/S2214-109X(20)30117-0 32353319

[B59] Vojinovic D. van der Lee S. J. van Duijn C. M. Vernooij M. W. Kavousi M. Amin N. (2018). Metabolic profiling of intra- and extracranial carotid artery atherosclerosis. *Atherosclerosis* 272 60–65. 10.1016/j.atherosclerosis.2018.03.015 29550646

[B60] Wahlstrom A. Sayin S. I. Marschall H. U. Backhed F. (2016). Intestinal Crosstalk between Bile Acids and Microbiota and Its Impact on Host Metabolism. *Cell Metab* 24 41–50. 10.1016/j.cmet.2016.05.005 27320064

[B61] Wang Z. Klipfell E. Bennett B. J. Koeth R. Levison B. S. Dugar B. (2011). Gut flora metabolism of phosphatidylcholine promotes cardiovascular disease. *Nature* 472 57–63. 10.1038/nature09922 21475195 PMC3086762

[B62] Weber C. Noels H. (2011). Atherosclerosis: current pathogenesis and therapeutic options. *Nat Med* 17 1410–1422. 10.1038/nm.2538 22064431

[B63] Witkowski M. Weeks T. L. Hazen S. L. (2020). Gut Microbiota and Cardiovascular Disease. *Circ Res* 127 553–570. 10.1161/circresaha.120.316242 32762536 PMC7416843

[B64] Won T. J. Kim B. Song D. S. Lim Y. T. Oh E. S. Lee D. I. (2011). Modulation of Th1/Th2 balance by Lactobacillus strains isolated from Kimchi via stimulation of macrophage cell line J774A.1 in vitro. *J Food Sci* 76 H55–H61. 10.1111/j.1750-3841.2010.02031.x 21535768

[B65] Yang Y. Gu H. Sun Q. Wang J. (2018). Effects of Christensenella minuta lipopolysaccharide on RAW 264.7 macrophages activation. *Microb Pathog* 125 411–417. 10.1016/j.micpath.2018.10.005 30290268

[B66] Zheng J. Hoffman K. L. Chen J. S. Shivappa N. Sood A. Browman G. J. (2020). Dietary inflammatory potential in relation to the gut microbiome: results from a cross-sectional study. *Br J Nutr* 124 931–942. 10.1017/s0007114520001853 32475373 PMC7554089

[B67] Zhou C. Zhao H. Xiao X. Y. Chen B. D. Guo R. J. Wang Q. (2020). Metagenomic profiling of the pro-inflammatory gut microbiota in ankylosing spondylitis. *J Autoimmun* 107 102360. 10.1016/j.jaut.2019.102360 31806420

[B68] Ziganshina E. E. Sharifullina D. M. Lozhkin A. P. Khayrullin R. N. Ignatyev I. M. Ziganshin A. M. (2016). Bacterial Communities Associated with Atherosclerotic Plaques from Russian Individuals with Atherosclerosis. *PLoS One* 11:e0164836. 10.1371/journal.pone.0164836 27736997 PMC5063344

