# Supplementary figures and images for: Exploration of Crucial Mediators for Carotid Atherosclerosis Pathogenesis Through Integration of Microbiome, Metabolome, and Transcriptome
Source: Front Physiol. 2021 May 24;12:645212. doi: 10.3389/fphys.2021.645212 (PMC8181762; doi:10.3389/fphys.2021.645212)

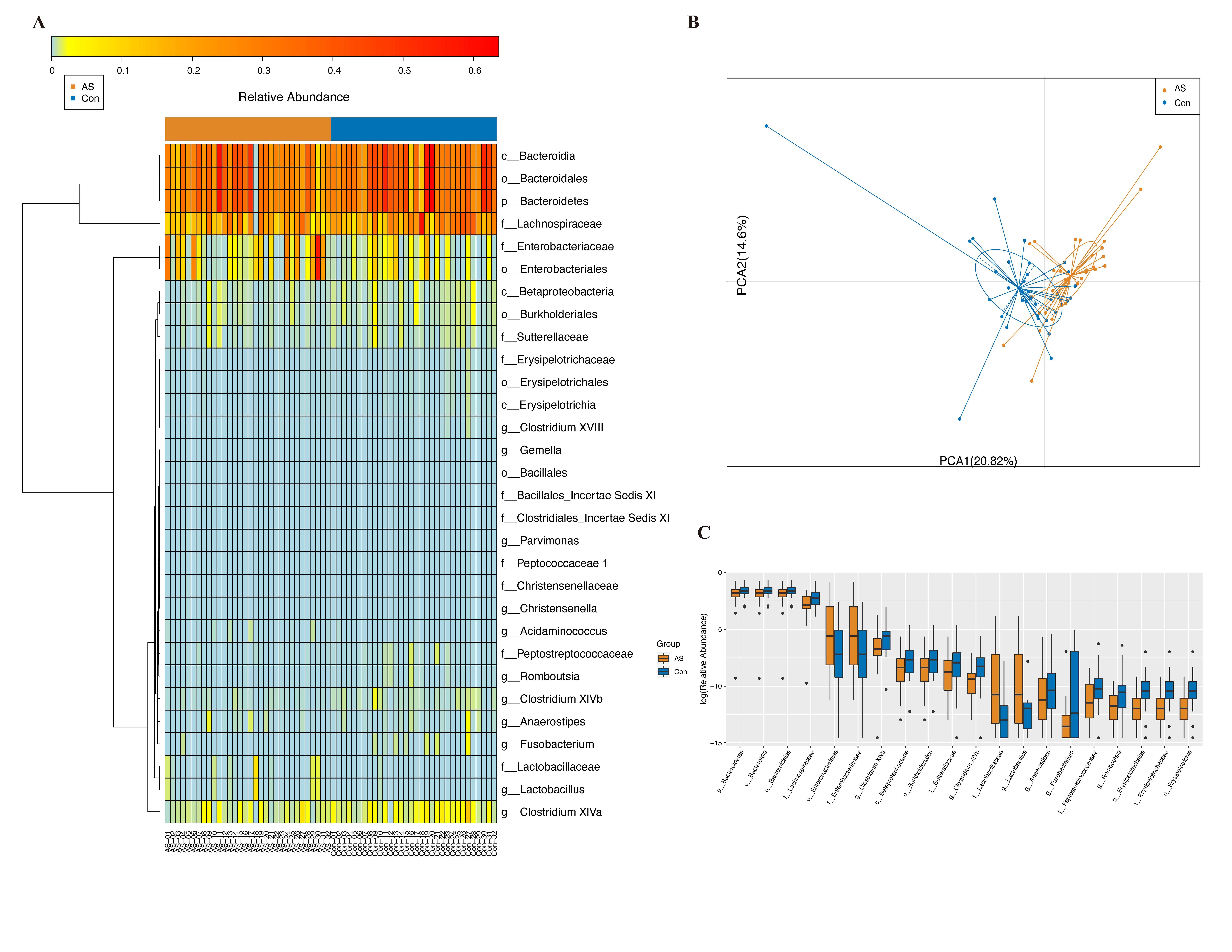

Supplement: Supplementary Figure 2 — Differentially enriched microbiota for CAS patients and healthy controls from all levels. (A) The differentially enriched gut microbiotas from all levels were visualized in heatmap. (B) PCA plot demonstrated that CAS group is significantly different from control group based on differential microbiotas from all levels. (C) The differentially enriched gut microbiotas were visualized in box plot. [file Image_2.TIF]
