# Supplementary material for: Exploration of Crucial Mediators for Carotid Atherosclerosis Pathogenesis Through Integration of Microbiome, Metabolome, and Transcriptome
Source: Front Physiol. 2021 May 24;12:645212. doi: 10.3389/fphys.2021.645212 (PMC8181762; doi:10.3389/fphys.2021.645212)
Supplement: Supplementary Table 1 — The α-diversity for CAS and healthy controls. [file Table_1.DOCX]

**Table S1. The α-diversity for CAS and healthy controls.**

| **Samples** | **chao1** | **observed_species** | **PD_whole_tree** | **shannon** | **simpson** | **goods_coverage** |
| --- | --- | --- | --- | --- | --- | --- |
| **AS-01** | 283 | 248 | 19.21582 | 4.275959235 | 0.868725153 | 0.997916667 |
| **AS-02** | 286.9411765 | 264 | 19.05004 | 5.466699309 | 0.956385045 | 0.998333333 |
| **AS-03** | 355.6222222 | 325 | 22.20652 | 5.552686862 | 0.944223135 | 0.997791667 |
| **AS-04** | 233.5 | 205 | 14.7078 | 4.500968807 | 0.896748635 | 0.998375 |
| **AS-05** | 271 | 232 | 16.35304 | 5.132908006 | 0.93881617 | 0.998333333 |
| **AS-06** | 152.3333333 | 137 | 8.89032 | 3.465655699 | 0.839009601 | 0.999 |
| **AS-07** | 208.0588235 | 177 | 13.10903 | 4.37576497 | 0.897931906 | 0.998625 |
| **AS-08** | 289.28125 | 251 | 17.99802 | 4.778889489 | 0.928684399 | 0.997916667 |
| **AS-09** | 121.0666667 | 112 | 9.13069 | 4.191991058 | 0.898004705 | 0.999291667 |
| **AS-10** | 179.5263158 | 165 | 11.28044 | 4.697157132 | 0.924170583 | 0.999 |
| **AS-11** | 103.4285714 | 84 | 7.23658 | 3.60100757 | 0.863675028 | 0.999291667 |
| **AS-12** | 149.0909091 | 124 | 10.1061 | 3.554007609 | 0.826212035 | 0.999 |
| **AS-13** | 112 | 95 | 7.64497 | 3.440867649 | 0.847947733 | 0.99925 |
| **AS-14** | 105 | 92 | 7.84537 | 3.783161502 | 0.872799375 | 0.999416667 |
| **AS-15** | 129.75 | 121 | 9.05731 | 4.442762583 | 0.908960608 | 0.999375 |
| **AS-16** | 219.0909091 | 165 | 14.72076 | 3.739173227 | 0.83971066 | 0.998541667 |
| **AS-17** | 147.625 | 116 | 9.56002 | 4.424288331 | 0.924945236 | 0.999041667 |
| **AS-18** | 121.2727273 | 79 | 7.97918 | 1.955316638 | 0.577139295 | 0.998708333 |
| **AS-19** | 196.1176471 | 177 | 11.57166 | 4.887173715 | 0.940956722 | 0.998916667 |
| **AS-20** | 223.0555556 | 190 | 14.89459 | 5.012894471 | 0.930409528 | 0.998541667 |
| **AS-21** | 341.375 | 252 | 17.64518 | 5.379488216 | 0.955345063 | 0.99725 |
| **AS-22** | 199.5 | 180 | 12.90758 | 4.287452838 | 0.871115566 | 0.998875 |
| **AS-23** | 197.2352941 | 181 | 11.90098 | 5.360525706 | 0.958145858 | 0.999 |
| **AS-24** | 218.3703704 | 188 | 11.71767 | 4.260956576 | 0.88306524 | 0.998291667 |
| **AS-25** | 316.2727273 | 256 | 17.06418 | 5.324033173 | 0.948973747 | 0.997833333 |
| **AS-26** | 236.1666667 | 202 | 14.13698 | 4.167538516 | 0.880925632 | 0.998291667 |
| **AS-27** | 255.047619 | 216 | 14.19666 | 4.637253501 | 0.886061115 | 0.998291667 |
| **AS-28** | 240.3 | 207 | 13.49735 | 4.008533808 | 0.842170469 | 0.998458333 |
| **AS-29** | 208.7142857 | 182 | 13.33489 | 4.470681275 | 0.911796438 | 0.998583333 |
| **AS-30** | 167.6666667 | 156 | 10.95856 | 3.491621679 | 0.731449528 | 0.999125 |
| **AS-31** | 353.8235294 | 300 | 20.59732 | 5.328035066 | 0.94300359 | 0.997458333 |
| **AS-32** | 274.125 | 231 | 15.37249 | 4.771719721 | 0.916317913 | 0.998083333 |
| **Con-01** | 180.6470588 | 163 | 11.61286 | 4.217613727 | 0.885457944 | 0.998958333 |
| **Con-02** | 301.3571429 | 266 | 16.96645 | 5.395790935 | 0.943560427 | 0.998125 |
| **Con-03** | 243.7142857 | 217 | 14.68428 | 5.195797762 | 0.937212722 | 0.998583333 |
| **Con-04** | 170 | 141 | 10.1531 | 3.632915217 | 0.826318806 | 0.99875 |
| **Con-05** | 239.6470588 | 189 | 13.17023 | 4.517098438 | 0.898503997 | 0.99825 |
| **Con-06** | 176.5263158 | 162 | 11.51872 | 4.229888026 | 0.839962931 | 0.999 |
| **Con-07** | 268.6470588 | 213 | 15.99427 | 4.519410022 | 0.856676181 | 0.998166667 |
| **Con-08** | 215 | 184 | 11.97606 | 4.872342885 | 0.933728722 | 0.998666667 |
| **Con-09** | 122.5 | 112 | 9.09623 | 4.630597685 | 0.929215295 | 0.999375 |
| **Con-10** | 104.5 | 97 | 7.75401 | 3.616855552 | 0.817788611 | 0.999583333 |
| **Con-11** | 116.3636364 | 104 | 8.29835 | 3.976196243 | 0.870034691 | 0.999291667 |
| **Con-12** | 126.2727273 | 99 | 8.22437 | 3.157325401 | 0.822864667 | 0.998958333 |
| **Con-13** | 236.125 | 166 | 12.85102 | 4.215800295 | 0.901889833 | 0.998583333 |
| **Con-14** | 288.1 | 202 | 14.02495 | 4.351977646 | 0.847342 | 0.99825 |
| **Con-15** | 170.8823529 | 156 | 11.32717 | 4.014262864 | 0.866944858 | 0.999041667 |
| **Con-16** | 151.4285714 | 116 | 9.87832 | 3.809833196 | 0.851915146 | 0.998666667 |
| **Con-17** | 210.5555556 | 188 | 14.06441 | 4.57703806 | 0.910249795 | 0.998791667 |
| **Con-18** | 158.625 | 135 | 10.62809 | 4.285711586 | 0.891420861 | 0.998833333 |
| **Con-19** | 121.3636364 | 109 | 8.92057 | 4.087756987 | 0.888473424 | 0.999291667 |
| **Con-20** | 206 | 155 | 11.5074 | 2.772486057 | 0.631759507 | 0.998583333 |
| **Con-21** | 124 | 85 | 7.37775 | 4.052504848 | 0.886459483 | 0.999458333 |
| **Con-22** | 267.7857143 | 244 | 17.81266 | 5.370988169 | 0.946814094 | 0.998458333 |
| **Con-23** | 269.1176471 | 216 | 14.16462 | 5.089267062 | 0.925964733 | 0.998208333 |
| **Con-24** | 311.6774194 | 262 | 19.29719 | 4.863734954 | 0.908692969 | 0.997666667 |
| **Con-25** | 126.5 | 120 | 9.37454 | 4.268242945 | 0.909363875 | 0.999458333 |
| **Con-26** | 187.4285714 | 141 | 10.3752 | 4.364980275 | 0.91085892 | 0.998916667 |
| **Con-27** | 155.0714286 | 137 | 10.06654 | 4.724621788 | 0.937770903 | 0.999041667 |
| **Con-28** | 140 | 130 | 9.13122 | 4.513562935 | 0.909178618 | 0.999333333 |
| **Con-29** | 247.5384615 | 216 | 14.82849 | 5.17041829 | 0.939535483 | 0.998291667 |
| **Con-30** | 297.1153846 | 250 | 18.80912 | 4.460297591 | 0.852552476 | 0.997916667 |
| **Con-31** | 138 | 119 | 8.78412 | 4.040009444 | 0.883041736 | 0.999166667 |
| **Con-32** | 216.5384615 | 202 | 12.57636 | 4.733642473 | 0.916956972 | 0.998833333 |
