# Supplementary material for: Exploration of Crucial Mediators for Carotid Atherosclerosis Pathogenesis Through Integration of Microbiome, Metabolome, and Transcriptome
Source: Front Physiol. 2021 May 24;12:645212. doi: 10.3389/fphys.2021.645212 (PMC8181762; doi:10.3389/fphys.2021.645212)
Supplement: Supplementary Table 2 — Differentially enriched microbiota from all levels. [file Table_2.DOCX]

**Table S2. Differentially enriched microbiota from all levels.**

| **Gut microbiota** | **mean(AS)** | **mean(Con)** | ***p*-value** | **median(AS)** | **median(Con)** |
| --- | --- | --- | --- | --- | --- |
| **c__Bacteroidia** | 0.286696985 | 0.344130717 | 0.044175838 | -1.822630045 | -1.639953967 |
| **c__Betaproteobacteria** | 0.004234667 | 0.007518191 | 0.032759766 | -8.363924715 | -7.671935598 |
| **c__Erysipelotrichia** | 0.000401507 | 0.001045998 | 0.018658504 | -11.96878677 | -10.42978552 |
| **f__Bacillales_Incertae Sedis XI** | 3.9E-06 | 2.08E-05 | 0.014781178 | -14.55374927 | -14.55374927 |
| **f__Christensenellaceae** | 4.29E-05 | 3.9E-06 | 0.027190901 | -13.55374927 | -12.96878677 |
| **f__Clostridiales_Incertae Sedis XI** | 1.3E-06 | 2.21E-05 | 0.005882137 | -14.55374927 | -14.55374927 |
| **f__Enterobacteriaceae** | 0.087091996 | 0.025766632 | 0.040588297 | -5.569820939 | -7.201149085 |
| **f__Erysipelotrichaceae** | 0.000401507 | 0.001045998 | 0.018658504 | -11.96878677 | -10.42978552 |
| **f__Lachnospiraceae** | 0.171820426 | 0.230301455 | 0.040011568 | -2.83953072 | -2.235856785 |
| **f__Lactobacillaceae** | 0.004282744 | 0.000178015 | 0.004238602 | -10.74639435 | -12.96878677 |
| **f__Peptococcaceae 1** | 0 | 2.73E-05 | 0.042147909 | NA | -12.26126802 |
| **f__Peptostreptococcaceae** | 0.000596414 | 0.001971154 | 0.009304567 | -11.46878677 | -10.23182118 |
| **f__Sutterellaceae** | 0.002954782 | 0.005370322 | 0.04671681 | -8.74662441 | -7.939039429 |
| **g__Acidaminococcus** | 0.000880977 | 0.00010395 | 0.004853071 | -10.30582176 | -9.853309555 |
| **g__Anaerostipes** | 0.001823025 | 0.00227001 | 0.033041905 | -11.23182118 | -10.38382427 |
| **g__Christensenella** | 4.29E-05 | 3.9E-06 | 0.027190901 | -13.55374927 | -12.96878677 |
| **g__Clostridium XVIII** | 0.000165021 | 0.000632796 | 0.008816879 | -12.96878677 | -12.23182118 |
| **g__Clostridium XlVa** | 0.014102131 | 0.024426975 | 0.011581483 | -6.754083402 | -5.596422402 |
| **g__Clostridium XlVb** | 0.001576143 | 0.00431263 | 0.000456455 | -9.352776212 | -8.268347055 |
| **g__Fusobacterium** | 0.000284563 | 0.002597453 | 0.043528042 | -13.55374927 | -12.39278523 |
| **g__Gemella** | 3.9E-06 | 2.08E-05 | 0.014781178 | -14.55374927 | -14.55374927 |
| **g__Lactobacillus** | 0.004282744 | 0.000174116 | 0.001399704 | -10.74639435 | -11.96878677 |
| **g__Parvimonas** | 1.3E-06 | 1.69E-05 | 0.012301771 | -14.55374927 | -14.55374927 |
| **g__Romboutsia** | 0.000267672 | 0.001351351 | 0.000467579 | -11.74639435 | -10.55657256 |
| **o__Bacillales** | 3.9E-06 | 2.08E-05 | 0.014781178 | -14.55374927 | -14.55374927 |
| **o__Bacteroidales** | 0.286696985 | 0.344130717 | 0.044175838 | -1.822630045 | -1.639953967 |
| **o__Burkholderiales** | 0.004233368 | 0.007518191 | 0.032759766 | -8.363924715 | -7.671935598 |
| **o__Enterobacteriales** | 0.087091996 | 0.025766632 | 0.040588297 | -5.569820939 | -7.201149085 |
| **o__Erysipelotrichales** | 0.000401507 | 0.001045998 | 0.018658504 | -11.96878677 | -10.42978552 |
| **p__Bacteroidetes** | 0.286726871 | 0.344154106 | 0.044175838 | -1.822630045 | -1.6398599 |
