# Supplementary material for: Exploration of Crucial Mediators for Carotid Atherosclerosis Pathogenesis Through Integration of Microbiome, Metabolome, and Transcriptome
Source: Front Physiol. 2021 May 24;12:645212. doi: 10.3389/fphys.2021.645212 (PMC8181762; doi:10.3389/fphys.2021.645212)
Supplement: Supplementary Table 3 — Differential KEGG pathways predicted by PICRUSt. [file Table_3.DOCX]

**Table S3. Differential KEGG pathways predicted by PICRUSt.**

| **KEGG pathways** | **mean(AS)** | **mean(Con)** | ***p*-value** | **median(AS)** | **median(Con)** |
| --- | --- | --- | --- | --- | --- |
| **1,1,1-Trichloro-2,2-bis(4-chlorophenyl)ethane_(DDT)_degradation** | 1.4E-06 | 2.37E-06 | 0.017471441 | -20.05313282 | -19.15764848 |
| **African_trypanosomiasis** | 6.38E-05 | 1.9E-05 | 0.000358503 | -14.2674408 | -16.76056419 |
| **Alanine,_aspartate_and_glutamate_metabolism** | 0.010890796 | 0.011339453 | 0.044175838 | -6.50189556 | -6.46452164 |
| **Amino_sugar_and_nucleotide_sugar_metabolism** | 0.014297886 | 0.014949561 | 0.005479369 | -6.116569369 | -6.066609298 |
| **Amyotrophic_lateral_sclerosis_(ALS)** | 0.000118782 | 8E-05 | 0.005479369 | -13.4003542 | -14.10032572 |
| **Antigen_processing_and_presentation** | 0.00040184 | 0.000439582 | 0.038698555 | -11.22124845 | -11.14428629 |
| **Apoptosis** | 4.43E-06 | 1.56E-06 | 0.006508232 | -18.47485602 | -20.03908853 |
| **Atrazine_degradation** | 0.000189524 | 0.000140151 | 0.038698555 | -12.60865267 | -13.37894671 |
| **Bacterial_secretion_system** | 0.00647385 | 0.005943207 | 0.010270264 | -7.282562801 | -7.405996901 |
| **Biosynthesis_of_siderophore_group_nonribosomal_peptides** | 0.000380925 | 0.00022063 | 0.028424145 | -12.03415677 | -12.36579181 |
| **Biosynthesis_of_unsaturated_fatty_acids** | 0.001408851 | 0.001168756 | 0.00253858 | -9.628315889 | -9.847691051 |
| **Biosynthesis_of_vancomycin_group_antibiotics** | 0.000598186 | 0.000655079 | 0.02379082 | -10.70976669 | -10.59492198 |
| **Bisphenol_degradation** | 0.000689942 | 0.000789327 | 0.012049936 | -10.5423629 | -10.29237205 |
| **Bladder_cancer** | 2.93E-05 | 8.71E-06 | 0.044708952 | -16.37458818 | -18.43460792 |
| **Caprolactam_degradation** | 0.000327299 | 0.000159877 | 0.008032481 | -11.96256648 | -13.29294505 |
| **Carbon_fixation_in_photosynthetic_organisms** | 0.006530241 | 0.006759471 | 0.007388626 | -7.246004806 | -7.221639931 |
| **Carotenoid_biosynthesis** | 2.81E-05 | 1.99E-05 | 0.032662409 | -15.99121556 | -16.81919644 |
| **Cell_cycle_-_Caulobacter** | 0.004909443 | 0.005183095 | 0.036180557 | -7.637993564 | -7.57657024 |
| **Chagas_disease_(American_trypanosomiasis)** | 6.1E-05 | 1.83E-05 | 0.002098836 | -14.3405937 | -16.83200686 |
| **Chlorocyclohexane_and_chlorobenzene_degradation** | 0.000107066 | 7.49E-05 | 0.006790782 | -13.32324148 | -14.15292766 |
| **Chromosome** | 0.015449401 | 0.015915681 | 0.048694339 | -6.001773955 | -5.967235398 |
| **D-Arginine_and_D-ornithine_metabolism** | 2.39E-05 | 4.24E-05 | 0.000808867 | -16.49135779 | -14.75173661 |
| **Dioxin_degradation** | 0.000591369 | 0.000452492 | 0.005479369 | -10.74878613 | -11.14879584 |
| **Drug_metabolism_-_cytochrome_P450** | 0.000353223 | 0.000190265 | 0.000201935 | -11.69471129 | -12.67668293 |
| **Electron_transfer_carriers** | 0.000285038 | 0.00015261 | 0.007704621 | -12.57939511 | -13.01214616 |
| **Fatty_acid_metabolism** | 0.002504328 | 0.002090987 | 0.002098836 | -8.754508538 | -8.898833829 |
| **Fluorobenzoate_degradation** | 7.09E-05 | 2.6E-05 | 0.014093058 | -14.08020721 | -17.48284415 |
| **Function_unknown** | 0.013007048 | 0.011759713 | 0.005479369 | -6.330726098 | -6.448300185 |
| **Galactose_metabolism** | 0.007973421 | 0.008386164 | 0.037421803 | -6.998332266 | -6.89409028 |
| **Geraniol_degradation** | 0.000519918 | 0.000344705 | 0.019818731 | -11.11092849 | -11.62519697 |
| **Glutamatergic_synapse** | 0.001090293 | 0.001158961 | 0.045641595 | -9.792022145 | -9.728620802 |
| **Glycan_biosynthesis_and_metabolism** | 0.000394928 | 0.00027418 | 0.014093058 | -11.46935928 | -11.88572658 |
| **Glycosphingolipid_biosynthesis_-_globo_series** | 0.001176268 | 0.001338477 | 0.044175838 | -9.798347499 | -9.606722982 |
| **Inorganic_ion_transport_and_metabolism** | 0.002116176 | 0.001756994 | 0.010270264 | -8.937941657 | -9.316099765 |
| **Inositol_phosphate_metabolism** | 0.001004767 | 0.000858571 | 0.011581483 | -10.06892803 | -10.29152616 |
| **Limonene_and_pinene_degradation** | 0.000787701 | 0.00065782 | 0.034974068 | -10.32086255 | -10.60035797 |
| **Linoleic_acid_metabolism** | 0.000575291 | 0.000668898 | 0.004021803 | -10.71375018 | -10.53752891 |
| **Metabolism_of_xenobiotics_by_cytochrome_P450** | 0.000347169 | 0.000184312 | 0.000169126 | -11.69233258 | -12.69551898 |
| **NOD-like_receptor_signaling_pathway** | 0.000415768 | 0.000466018 | 0.004398022 | -11.16086428 | -11.05601083 |
| **Nucleotide_metabolism** | 0.000568857 | 0.000423806 | 0.009091016 | -11.1156451 | -11.32807786 |
| **Oxidative_phosphorylation** | 0.010820467 | 0.011244159 | 0.030480999 | -6.509144532 | -6.487907578 |
| **Pantothenate_and_CoA_biosynthesis** | 0.006537341 | 0.006701547 | 0.016430797 | -7.241441242 | -7.195694841 |
| **Pertussis** | 0.000522006 | 0.000276496 | 0.018397575 | -11.61628744 | -12.62595714 |
| **Phosphatidylinositol_signaling_system** | 0.000814517 | 0.000753334 | 0.005479369 | -10.26095242 | -10.39761921 |
| **Photosynthesis** | 0.004082433 | 0.004487929 | 0.005021313 | -7.938501504 | -7.76378851 |
| **Photosynthesis_proteins** | 0.004117491 | 0.004507469 | 0.005021313 | -7.927951114 | -7.759993755 |
| **Polyketide_sugar_unit_biosynthesis** | 0.001912291 | 0.002147315 | 0.000338897 | -9.019149149 | -8.868489394 |
| **Porphyrin_and_chlorophyll_metabolism** | 0.0095398 | 0.010512297 | 0.007388626 | -6.659132465 | -6.535898125 |
| **Progesterone-mediated_oocyte_maturation** | 0.00040184 | 0.000439582 | 0.038698555 | -11.22124845 | -11.14428629 |
| **Propanoate_metabolism** | 0.004849815 | 0.004563664 | 0.015816945 | -7.708611727 | -7.776582534 |
| **Prostate_cancer** | 0.000421222 | 0.000458018 | 0.048694339 | -11.16663151 | -11.09529447 |
| **Protein_export** | 0.00570726 | 0.005951429 | 0.012534842 | -7.448126494 | -7.385687164 |
| **Protein_kinases** | 0.003182006 | 0.002920792 | 0.022946055 | -8.353109867 | -8.396838549 |
| **RNA_degradation** | 0.004500533 | 0.0046727 | 0.038698555 | -7.801430661 | -7.748717898 |
| **Renal_cell_carcinoma** | 5.97E-05 | 3.86E-05 | 0.044175838 | -14.13417516 | -15.00790857 |
| **Retinol_metabolism** | 0.000289428 | 0.000218182 | 0.001419213 | -12.03445195 | -12.41255596 |
| **Secretion_system** | 0.012642282 | 0.010947517 | 0.002421492 | -6.362018269 | -6.536941809 |
| **Shigellosis** | 2.74E-07 | 8.22E-08 | 0.021590314 | -21.32996497 | -22.73623665 |
| **Streptomycin_biosynthesis** | 0.003063068 | 0.003245521 | 0.025560287 | -8.316663614 | -8.26292836 |
| **Transcription_related_proteins** | 9.39E-05 | 4.14E-05 | 0.008032481 | -13.92961576 | -15.38078013 |
| **Tryptophan_metabolism** | 0.001465635 | 0.00113194 | 0.006236141 | -9.574619951 | -9.875279177 |
| **Tyrosine_metabolism** | 0.003492675 | 0.003185795 | 9.2E-05 | -8.176775303 | -8.317963105 |
| **Ubiquitin_system** | 0.000115148 | 8.08E-05 | 0.027440684 | -13.37847883 | -13.85422469 |
| **Xylene_degradation** | 0.000544063 | 0.000434046 | 0.007084131 | -10.89703514 | -11.1774294 |
| **alpha-Linolenic_acid_metabolism** | 8.83E-05 | 2.92E-05 | 0.038698555 | -15.1233064 | -16.03187796 |
