# Supplementary material for: Exploration of Crucial Mediators for Carotid Atherosclerosis Pathogenesis Through Integration of Microbiome, Metabolome, and Transcriptome
Source: Front Physiol. 2021 May 24;12:645212. doi: 10.3389/fphys.2021.645212 (PMC8181762; doi:10.3389/fphys.2021.645212)
Supplement: Supplementary Table 4 — All differential metabolites between CAS patients and healthy controls. [file Table_4.DOCX]

**Table S4. All differential metabolites between CAS patients and healthy controls.** The threshold for screening differential metabolites was set as VIP>1 and *p*-value<0.05

| **Metabolites** | **VIP** | ***p*-value** | ***q*-value** | **log2(fold-change)** |
| --- | --- | --- | --- | --- |
| ***POS mode*** |  |  |  |  |
| Val-Tyr | 1.968716347 | 7.593E-06 | 6.46606E-05 | 0.866712352 |
| Val-Gly | 1.728767227 | 0.000155574 | 0.00060444 | 0.31419009 |
| Urea | 1.113926894 | 0.004951778 | 0.009550264 | 0.203786498 |
| Tyramine | 1.77634048 | 1.64159E-05 | 0.0001154 | 0.245329759 |
| Trimethylamine N-oxide | 1.176965998 | 0.044507701 | 0.059552368 | 0.618025622 |
| Trigonelline | 1.085045021 | 0.007168336 | 0.013098164 | 0.295008311 |
| trans-cinnamate | 1.69317961 | 6.51626E-05 | 0.000333409 | 0.224123272 |
| trans-2-Hydroxycinnamic acid | 1.725290074 | 6.28595E-05 | 0.000325099 | 0.225594417 |
| Thioetheramide-PC | 1.387456726 | 0.000212727 | 0.000766595 | -0.375292343 |
| Taurine | 1.523264842 | 0.000251445 | 0.000863809 | 0.398372702 |
| Stearoylcarnitine | 1.562813615 | 2.59123E-05 | 0.000166304 | -0.525701618 |
| Squalene | 1.346785751 | 0.004080831 | 0.008073524 | -0.357506115 |
| S-Methyl-5'-thioadenosine | 2.262910285 | 1.7841E-08 | 4.04415E-07 | 0.715372358 |
| Ser-Pro | 1.724570214 | 5.32913E-05 | 0.00028856 | 0.49971007 |
| Securinine | 1.926987557 | 1.98839E-07 | 3.18388E-06 | 0.446152458 |
| SANGUINARINE | 2.124476231 | 9.29768E-07 | 1.20433E-05 | 0.769441459 |
| Ribothymidine | 1.93154087 | 1.57675E-05 | 0.00011213 | 0.678626329 |
| Pyroglutamic acid | 2.002007682 | 4.63083E-10 | 1.59367E-08 | -0.692078945 |
| Pseudouridine | 1.75100217 | 3.10804E-05 | 0.000192267 | 0.694286656 |
| Pro-Tyr | 1.990421832 | 1.02041E-05 | 8.13289E-05 | 0.625886732 |
| Pro-Thr | 1.323967694 | 0.021357613 | 0.032851827 | 0.334507525 |
| Propoxur | 2.785113538 | 2.69771E-20 | 9.78414E-18 | -2.406871344 |
| Propionylglycine | 1.045412164 | 0.015815648 | 0.025672429 | 0.733016154 |
| Promethazine | 1.181126734 | 0.009257348 | 0.016135112 | 0.441451509 |
| Pro-Met | 1.522549552 | 0.00105591 | 0.002651469 | -0.681147264 |
| Pro-Asn | 1.409315932 | 0.003434318 | 0.006958427 | 0.270922967 |
| Pristanic acid | 1.750100406 | 1.85662E-05 | 0.000125863 | -0.837282245 |
| Phosphocreatine | 1.494487377 | 3.4035E-05 | 0.000206284 | -0.50733926 |
| Phe-Thr | 1.743670792 | 0.000218243 | 0.000781022 | 0.345462224 |
| Phe-Phe | 1.220315518 | 0.003138974 | 0.006435798 | 0.201726482 |
| Phenylpropionylglycine | 2.053948288 | 1.56166E-08 | 3.62248E-07 | -0.745181027 |
| Phenylacetic acid | 1.839111629 | 3.99837E-06 | 3.87116E-05 | 0.259077905 |
| Phenethyl Caffeiate | 1.03307772 | 0.008608502 | 0.015222114 | -0.317289348 |
| Phe-Cys | 1.536176265 | 0.002504104 | 0.005325831 | 0.303959955 |
| Phe-Asn | 1.134703737 | 0.01463854 | 0.024044093 | 0.384291069 |
| P-Fluorophenylalanine | 1.018225781 | 1.03901E-07 | 1.83424E-06 | 0.550910872 |
| p-CHLOROPHENYLALANINE | 1.142375723 | 0.002161211 | 0.004726182 | 0.051264249 |
| Pantothenate | 1.204098977 | 0.00345564 | 0.00699606 | 0.303555007 |
| Palmitoyl ethanolamide | 1.888006846 | 3.27146E-08 | 6.59169E-07 | -0.519486911 |
| O-Desmethylnaproxen | 2.140136711 | 9.1044E-09 | 2.26664E-07 | -1.017466992 |
| O-Acetyl-L-serine | 1.542654121 | 0.009513553 | 0.016488462 | 0.300304385 |
| Norfloxacin | 1.651872706 | 0.000817207 | 0.00217325 | 0.392969001 |
| N-Oleoylethanolamine | 1.712495265 | 4.00072E-06 | 3.87273E-05 | -0.471725575 |
| N-Benzyloxycarbonylglycine | 1.155213473 | 0.004214686 | 0.008297861 | 0.19998971 |
| N-Acetylglutamine | 1.738042841 | 0.002663556 | 0.005593616 | 0.505832835 |
| N-Acetylaspartylglutamate (NAAG) | 1.256410403 | 0.021635055 | 0.03319152 | 0.271024212 |
| N6-Acetyl-L-lysine | 1.164094536 | 0.020278905 | 0.031513978 | 0.219811237 |
| N2,N2-Dimethylguanosine | 1.955239812 | 1.36291E-07 | 2.29909E-06 | 0.502586008 |
| Monoethylglycylxylidide (MEGX) | 1.609253177 | 0.00078352 | 0.002104721 | -0.827628155 |
| Mimosine | 1.548825293 | 2.02517E-05 | 0.000135539 | 0.623250029 |
| Met-Tyr | 2.097377405 | 1.37094E-05 | 0.000101541 | 0.599122077 |
| Met-Ser | 2.033321381 | 1.34168E-10 | 5.12218E-09 | -0.768625451 |
| Methoxyacetic acid | 1.749774991 | 3.8475E-05 | 0.000226312 | 0.464294213 |
| Marmesin | 1.730151451 | 0.000934391 | 0.00240131 | 0.271300739 |
| Maltotriose | 1.066367692 | 0.018562754 | 0.029327785 | 0.542277124 |
| L-Valine | 1.633321741 | 5.67226E-06 | 5.15103E-05 | 0.435614113 |
| L-Tyrosine | 1.580685439 | 0.000602506 | 0.001711411 | 0.23311129 |
| L-Pyroglutamic acid | 1.041614598 | 0.021617755 | 0.03317039 | 0.19314101 |
| L-Proline | 1.670822006 | 0.000105574 | 0.0004549 | 0.230457848 |
| L-Pipecolic acid | 1.067691823 | 0.003893388 | 0.007755672 | 0.66047048 |
| L-Palmitoylcarnitine | 1.473412745 | 9.22086E-05 | 0.000419197 | -0.445282162 |
| L-NG-Monomethylarginine | 1.113521645 | 0.009565374 | 0.01655945 | 0.270493041 |
| L-Methionine | 1.500182354 | 0.000488488 | 0.001439616 | 0.166071614 |
| L-Isoleucine | 1.192551832 | 0.002253991 | 0.004891747 | 0.322479617 |
| Linoleoyl ethanolamide | 1.560528961 | 6.95179E-05 | 0.000348649 | -0.408199987 |
| L-Iditol | 1.831340742 | 0.000380277 | 0.001164371 | 0.386547675 |
| L-homoserine | 1.091089356 | 0.023516117 | 0.03550956 | 0.234780498 |
| L-Glutamate | 1.400496093 | 0.002491266 | 0.005303973 | 0.224774379 |
| L-Fucose | 1.816950183 | 0.000459324 | 0.001366867 | 0.438302257 |
| Leu-Val | 1.063232132 | 0.002846463 | 0.005920114 | -0.34287064 |
| L-Cystine | 1.399139992 | 0.000262486 | 0.000889889 | 0.264743893 |
| L-Citrulline | 1.543531225 | 0.000321417 | 0.001030134 | 0.264386809 |
| L-Carnitine | 1.4554022 | 0.000820402 | 0.002179678 | 0.262197346 |
| Larixinic Acid | 1.881451816 | 5.0817E-06 | 4.71089E-05 | 0.714354224 |
| L-Arabinose | 1.326549217 | 0.00032689 | 0.001043142 | 0.325264084 |
| Kynuramine | 1.505883554 | 0.002554599 | 0.005411369 | 0.344506081 |
| Ile-Tyr | 1.884641495 | 0.000108174 | 0.000461414 | 0.668456299 |
| Ile-Pro | 1.045602418 | 0.026701886 | 0.039384755 | -0.170937778 |
| Ile-Asn | 1.003212041 | 0.000688774 | 0.00190434 | -0.216793157 |
| Hypoxanthine | 1.581803559 | 3.00919E-08 | 6.18441E-07 | -0.641440472 |
| Hydrocortisone (Cortisol) | 1.229077692 | 0.001091855 | 0.002724021 | 0.410978198 |
| Homocitrate | 1.978541617 | 0.000652259 | 0.001823948 | 1.047764919 |
| His-Tyr | 1.932403857 | 3.25098E-05 | 0.000199119 | 0.605097266 |
| His-Pro | 1.039320629 | 0.024441891 | 0.036654521 | 0.335350314 |
| His-Met | 1.541398947 | 0.000881632 | 0.002300559 | 0.37193572 |
| His-Cys | 1.634616969 | 0.000663873 | 0.001849715 | 0.332229708 |
| Gly-Pro | 2.796795801 | 1.88336E-19 | 4.55375E-17 | -2.603752686 |
| Glycylproline | 1.343551618 | 0.003172996 | 0.006494828 | -0.754814004 |
| Glycyl-L-leucine | 1.140784112 | 0.02483084 | 0.037130889 | -0.395006926 |
| Glycine | 2.010996597 | 1.01513E-06 | 1.29182E-05 | 0.403378885 |
| Glycerophosphocholine | 1.110692249 | 0.000436156 | 0.001308626 | -0.234982808 |
| Glutaraldehyde | 1.576150323 | 0.000296916 | 0.000970486 | 0.216640825 |
| Folate | 2.103566907 | 3.78928E-06 | 3.72917E-05 | 0.707428445 |
| Famciclovir | 1.963075042 | 6.85751E-06 | 5.98232E-05 | 0.671131708 |
| Ethanolamine | 2.816546298 | 6.3964E-18 | 8.96521E-16 | -2.509502083 |
| Erythrono-1,4-lactone | 1.16516823 | 0.007882459 | 0.014168618 | -0.274060671 |
| Ergothioneine | 1.859525099 | 4.67268E-05 | 0.000261532 | -0.965696937 |
| Eicosapentaenoic acid | 1.807845477 | 4.49425E-05 | 0.00025414 | -1.07267148 |
| EDTA | 1.082308054 | 0.012174838 | 0.020507496 | -0.219411167 |
| Dopamine | 1.674282048 | 7.99745E-05 | 0.000382886 | 0.216608073 |
| DL-Phenylalanine | 1.630633019 | 3.30198E-06 | 3.37938E-05 | 0.499980445 |
| DL-O-tyrosine | 1.151767613 | 0.005431068 | 0.010339944 | 0.122009301 |
| DL-3-Phenyllactic acid | 1.83763081 | 5.33549E-06 | 4.90226E-05 | 0.235647587 |
| DL-.alpha.-Phenylglycine | 1.535359216 | 0.000364084 | 0.00112865 | 0.446053449 |
| Dimethylglycine | 1.433951092 | 0.005017218 | 0.009659295 | 0.649952161 |
| Dimethylbenzimidazole | 2.347024371 | 7.03818E-07 | 9.59896E-06 | 1.016050071 |
| Diethylcarbamazine | 1.642884521 | 0.000557875 | 0.001607395 | 1.208993076 |
| Diaminopimelic acid | 1.252197842 | 7.89217E-05 | 0.000379581 | 0.391960866 |
| Diacetyl | 1.065738867 | 0.001176091 | 0.002890425 | 0.521117008 |
| D-gluconate | 1.598773014 | 0.003025433 | 0.006237378 | 0.34292695 |
| Desipramine | 1.084237893 | 0.018532366 | 0.029288417 | 0.237744163 |
| Decanoyl-L-carnitine | 1.911522868 | 1.30652E-05 | 9.80438E-05 | -1.289227648 |
| Cytosine | 1.305310522 | 5.05044E-05 | 0.000277263 | 0.456289248 |
| Carbaryl | 1.673131585 | 0.000621289 | 0.001754311 | 0.512202923 |
| beta-Octylglucoside | 1.689870607 | 0.001790534 | 0.004041947 | 0.443798062 |
| beta-Nicotinamide D-ribonucleotide | 1.88276969 | 0.000118476 | 0.000491248 | 0.364174418 |
| Betaine | 2.244004417 | 7.91245E-10 | 2.49541E-08 | 0.345335104 |
| Atrolactic acid | 1.459502508 | 0.002329544 | 0.005024731 | 0.765571835 |
| Arg-Tyr | 2.091951392 | 3.62525E-06 | 3.61447E-05 | 0.81197252 |
| Arg-Thr | 2.070117436 | 6.16297E-06 | 5.50332E-05 | 0.74464594 |
| Arg-Cys | 2.290707055 | 1.42425E-06 | 1.72295E-05 | 0.767399331 |
| Arecoline | 1.13840014 | 0.001401546 | 0.003312319 | -0.555820479 |
| Anthranilic acid (Vitamin L1) | 1.457150269 | 0.001588802 | 0.00365676 | 0.956929632 |
| Alpha-N-Phenylacetyl-L-glutamine | 1.762051456 | 0.000569636 | 0.001635096 | 1.436216381 |
| all cis-(6,9,12)-Linolenic acid | 1.263958527 | 0.001364913 | 0.003245985 | -0.549570716 |
| Ala-Thr | 1.267347741 | 0.000827102 | 0.002193119 | -0.37659892 |
| Ala-Glu | 1.279379521 | 0.009156861 | 0.01599543 | 0.428414214 |
| Ala-Ala | 1.348996185 | 0.000598304 | 0.001701745 | 0.848597304 |
| Adenosine | 1.819645303 | 0.000194967 | 0.000718744 | 0.431419663 |
| Adenine | 1.774892246 | 7.64626E-07 | 1.0271E-05 | 0.692954673 |
| Acyclovir | 1.722638727 | 0.000359907 | 0.001119292 | 0.985090868 |
| Acetylglycine | 1.273691327 | 0.034169555 | 0.048094509 | 0.228468885 |
| Acetylcarnitine | 1.00326012 | 0.004257212 | 0.008368678 | -0.242553272 |
| Acetoacetic acid | 1.325516601 | 0.000941733 | 0.002415089 | 0.543658206 |
| 9-Decen-1-ol | 1.451813757 | 1.41016E-05 | 0.000103627 | -0.599182295 |
| 6-Hydroxydopamine | 1.392515977 | 0.002561382 | 0.005422806 | 0.260952579 |
| 5-Methylcytosine | 1.201981936 | 0.001501688 | 0.003496612 | 0.170233599 |
| 5-Methoxytryptamine | 1.617951758 | 0.000103661 | 0.000450019 | 0.429327191 |
| 4-Methoxycinnamic acid | 1.258268441 | 0.022550521 | 0.03429989 | -0.47898624 |
| 4-Imidazoleacetic acid | 1.317527891 | 0.002862932 | 0.005949543 | -0.306771693 |
| 4-Hydroxycinnamic acid | 1.638915638 | 0.000133654 | 0.000535626 | 0.215494948 |
| 3'-O-methyladenosine | 1.075053478 | 0.003590298 | 0.007232357 | 0.336399877 |
| 3-Methylindole | 1.039581597 | 0.010556702 | 0.018084857 | 0.086996147 |
| 3-methylcytidine | 1.743654555 | 0.000219484 | 0.000784242 | 0.404169361 |
| 3-Methoxy-4-Hydroxyphenylglycol Sulfate | 1.673158751 | 0.000206213 | 0.000749297 | 1.007090541 |
| 3-Methoxy-4-hydroxyphenylethyleneglycol | 1.762321032 | 5.34627E-07 | 7.61591E-06 | -0.481114018 |
| 3-Hydroxykynurenine | 1.957182874 | 0.000107992 | 0.000460963 | 0.433522116 |
| 3-Deoxy-2-keto-6-phosphogluconic acid | 1.057078323 | 0.0026221 | 0.005524645 | -0.75248603 |
| 2-Phenylbutyric acid | 1.739445164 | 9.65777E-05 | 0.000431293 | 0.502374744 |
| 2-Octenoic acid | 1.759448593 | 6.26511E-05 | 0.000324338 | 0.364015301 |
| 2-Methylguanosine | 1.553167295 | 0.000494374 | 0.001454145 | 0.265151224 |
| 2-Methylbutyroylcarnitine | 1.00722319 | 0.013588889 | 0.022559078 | 0.352833947 |
| 2-Keto valeric acid | 1.084320887 | 0.031584131 | 0.045060257 | 0.226568019 |
| 2-Ethoxyethanol | 2.064983345 | 1.02523E-07 | 1.81426E-06 | -0.750281828 |
| 2-Amino-2-methyl-1,3-propanediol | 1.569212468 | 0.000677569 | 0.001879864 | 0.209904583 |
| 25-Hydroxycholesterol | 1.688387549 | 6.78212E-06 | 5.9314E-05 | -0.664373585 |
| 1-Stearoyl-2-hydroxy-sn-glycero-3-phosphoethanolamine | 1.455658698 | 0.000105216 | 0.000453992 | -0.436531275 |
| 1-Palmitoyl-sn-glycero-3-phosphocholine | 1.360056522 | 0.000671848 | 0.001867303 | -0.277911333 |
| 1-Aminocyclopropanecarboxylic acid | 1.738216094 | 5.99465E-06 | 5.38381E-05 | -0.606223618 |
| 1-Aminocyclohexanecarboxylic acid | 1.206883011 | 0.014942126 | 0.024467746 | -0.908207159 |
| 16-hydroxy hexadecanoic acid | 2.171638168 | 1.79641E-09 | 5.30718E-08 | -0.971129306 |
| 1,7-Dimethyluric acid | 1.360159962 | 0.01503051 | 0.0245906 | 0.299502325 |
| .gamma.-L-Glu-.epsilon.-L-Lys | 2.06550929 | 8.68074E-06 | 7.15537E-05 | 0.624444897 |
| (S)-2-Hydroxyglutarate | 1.039012189 | 0.002556289 | 0.00541422 | 0.269747068 |
| (-)-Medicarpin | 1.326441367 | 0.049476364 | 0.064845951 | 0.269010037 |
| ***NEG mode*** |  |  |  |  |
| Dihydroxyacetone | 1.178026061 | 0.006132643 | 0.010980585 | 0.240623996 |
| Isobutyric acid | 1.253867958 | 0.001358886 | 0.003021999 | 0.482350901 |
| DL-lactate | 1.842315066 | 1.03575E-08 | 1.86444E-07 | -0.459427105 |
| p-Cresol | 1.06355216 | 0.00200094 | 0.004195594 | 0.453003721 |
| Taurine | 1.979405278 | 1.51349E-06 | 1.28255E-05 | 0.437903609 |
| D-Arabinono-1,4-lactone | 1.022189809 | 0.049910467 | 0.055806478 | 0.234329715 |
| N-Acetyl-L-alanine | 1.545951397 | 0.000614233 | 0.001526586 | 0.270949506 |
| D-Ornithine | 1.802969298 | 1.57263E-06 | 1.31711E-05 | 0.289601921 |
| Adenine | 1.746636319 | 0.000246429 | 0.000716791 | 0.417124805 |
| Hypoxanthine | 1.840356755 | 8.14922E-11 | 2.47767E-09 | -0.739180414 |
| L-Threonate | 1.698041312 | 0.000598009 | 0.001494075 | 0.303448541 |
| Salicylic acid | 1.734352898 | 0.01921337 | 0.0277024 | 4.145007415 |
| 3-Aminopropanesulphonic Acid | 1.039813313 | 0.040379565 | 0.047806112 | 1.796022962 |
| 6-Hydroxynicotinic acid | 1.206818152 | 0.029194166 | 0.037505435 | 2.079415637 |
| L-Glutamate | 1.418368522 | 0.000884404 | 0.002073986 | 0.209626863 |
| L-Methionine | 1.298434632 | 0.000276978 | 0.000790714 | 0.23715414 |
| D-Ribose | 1.471734111 | 0.001142356 | 0.002601819 | 0.359586969 |
| Xanthine | 1.309461528 | 0.001111919 | 0.002541124 | -0.325374324 |
| L-Histidine | 1.196353937 | 0.010801202 | 0.017657903 | -0.222469716 |
| Formylanthranilic acid | 1.949132714 | 0.000220127 | 0.000651909 | 1.426589827 |
| L-Phenylalanine | 1.318641761 | 0.001361295 | 0.003026562 | 0.163770635 |
| Glycerol 3-phosphate | 1.241515084 | 0.002630925 | 0.005321639 | -0.148801079 |
| Maleamic acid | 1.242721859 | 0.001211316 | 0.002737823 | 0.335066177 |
| L-Citrulline | 1.891168687 | 1.48918E-06 | 1.2681E-05 | 0.307008611 |
| Gulonolactone | 1.031298655 | 0.022190624 | 0.03081356 | 0.528750832 |
| Xanthopterin | 2.153639262 | 5.0361E-11 | 1.65249E-09 | -1.016011938 |
| myo-Inositol | 1.841102137 | 7.5895E-05 | 0.000258168 | 0.290618288 |
| D-Mannose | 1.564386737 | 0.000125399 | 0.000398871 | 0.226978004 |
| D-Sorbitol | 1.751600349 | 0.00033603 | 0.000928565 | 0.565650644 |
| 7-Methylxanthine | 1.298730875 | 0.001693739 | 0.003634135 | 0.426975309 |
| Thymine | 1.831842597 | 1.51679E-07 | 1.77992E-06 | -0.375535048 |
| N6-Acetyl-L-lysine | 1.086253844 | 0.015744702 | 0.023841394 | 0.142275644 |
| 3-Hydroxycapric acid | 1.739086212 | 1.53181E-05 | 7.23588E-05 | -0.795086946 |
| N-Acetyl-DL-methionine | 1.185020629 | 0.012456676 | 0.019812113 | 0.408680633 |
| D-galacturonic acid | 1.97825815 | 1.75271E-05 | 8.0357E-05 | 0.378513381 |
| Galactonic acid | 1.155865817 | 0.019537552 | 0.02805269 | 0.283241141 |
| 5,6,7,8-tetrahydro-2-Naphthoic Acid | 2.483967886 | 7.33974E-11 | 2.27499E-09 | -0.837075472 |
| Dodecanoic acid | 1.612149539 | 0.004595188 | 0.008596585 | -0.74834496 |
| N-Acetylmannosamine | 1.571858766 | 6.38728E-05 | 0.000223976 | 0.303041731 |
| N1-Methyl-4-pyridone-3-carboxamide | 2.443377406 | 8.40619E-14 | 3.7485E-12 | -1.180267083 |
| m-Chlorohippuric acid | 1.209759886 | 0.00251502 | 0.00512048 | 0.248316573 |
| Tridecanoic acid (Tridecylic acid) | 1.549246762 | 0.00138576 | 0.003072769 | -0.542805973 |
| 3-Hydroxydodecanoic acid | 1.872339097 | 5.84638E-05 | 0.000209662 | -1.103961553 |
| Salicyluric acid | 1.428929548 | 0.027925205 | 0.036246094 | 1.744879658 |
| 5-L-Glutamyl-L-alanine | 1.395114469 | 0.002679748 | 0.005405593 | -0.293459061 |
| Pantothenate | 1.192135803 | 0.006981744 | 0.012247764 | 0.247464284 |
| L-Carnitine | 1.363042646 | 0.000561507 | 0.001419669 | 0.192747171 |
| D-Quinovose | 1.118902289 | 0.01325692 | 0.020829667 | -0.372182045 |
| Myristoleic acid | 1.378818223 | 0.004151645 | 0.007886098 | -0.521056163 |
| Myristic acid | 1.055498611 | 0.038241021 | 0.045879942 | -0.291726951 |
| Phenylacetylglycine | 1.609315395 | 1.20649E-07 | 1.48763E-06 | 1.576664728 |
| Glucosamine | 1.285534862 | 0.000425852 | 0.001128575 | 0.272173423 |
| Pentadecanoic Acid | 1.561493577 | 0.000123383 | 0.000393295 | -0.442814873 |
| Phosphorylcholine | 1.013751846 | 0.020133997 | 0.028689617 | -0.118992908 |
| Pseudouridine | 1.957899631 | 3.80293E-05 | 0.000149237 | 0.420185711 |
| D-Biotin | 2.385066256 | 4.85697E-11 | 1.60101E-09 | -2.067524087 |
| alpha-hydroxy myristic acid | 1.571173445 | 2.88029E-05 | 0.000119291 | -0.717083867 |
| Muramic acid | 1.947218111 | 4.41531E-05 | 0.000168455 | 0.614676863 |
| Ribothymidine | 1.143054812 | 0.044042908 | 0.050990091 | 0.20437864 |
| 1-Methylpseudouridine | 1.154669992 | 0.021244004 | 0.029849575 | 0.264266629 |
| 3-Methoxy-4-Hydroxyphenylglycol Sulfate | 1.277567181 | 0.007188383 | 0.012556872 | 0.380508005 |
| Alpha-N-Phenylacetyl-L-glutamine | 1.701961669 | 0.000390232 | 0.001051251 | 1.214340063 |
| Hexadecanedioic acid | 1.024861306 | 0.045226641 | 0.051989037 | -0.286195326 |
| Heptadecanoic acid | 1.731120134 | 2.87562E-05 | 0.000119132 | -0.558389033 |
| N-Acetyl-D-glucosamine | 1.530504569 | 0.002374562 | 0.004873155 | 0.336333823 |
| 5-Hydroxymethyluracil | 1.211213579 | 0.002948612 | 0.005876141 | 0.31729154 |
| Stearic acid | 1.125586201 | 0.001278415 | 0.002868178 | -0.213764548 |
| N4-Acetylcytidine | 1.564002648 | 0.000739543 | 0.00176667 | 0.426641546 |
| S-Methyl-5'-thioadenosine | 1.628238387 | 5.80186E-05 | 0.000208454 | 0.34927361 |
| Nname,cis-9,10-Epoxystearic acid | 2.11074521 | 5.1882E-10 | 1.18093E-08 | -0.579505119 |
| Pristanic acid | 1.933153123 | 1.57283E-05 | 7.38805E-05 | -0.781443205 |
| Arachidonic Acid (peroxide free) | 1.257122226 | 0.000662767 | 0.001621839 | 0.410289133 |
| N2,N2-Dimethylguanosine | 1.417847433 | 0.001543417 | 0.003364749 | 0.300108763 |
| Arachidic acid | 2.154753385 | 1.84744E-08 | 3.07886E-07 | -0.818933855 |
| (4Z,7Z,10Z,13Z,16Z,19Z)-4,7,10,13,1 6,19-Docosahexaenoic acid | 1.204800676 | 0.012722344 | 0.020152678 | -0.343649482 |
| Erucic acid | 1.417933116 | 0.00636558 | 0.011324839 | -0.668626565 |
| Behenic acid | 1.082074231 | 0.026186369 | 0.034644936 | -0.432739706 |
| D-Maltose | 1.068186326 | 0.017575547 | 0.025921923 | 0.236133051 |
| Maltitol | 1.045461133 | 0.011729843 | 0.018874903 | 0.255016864 |
| Tranilast | 1.885413773 | 1.87922E-05 | 8.47518E-05 | 0.407210316 |
| Tricosanoic acid | 1.127415959 | 0.039718733 | 0.047216358 | -0.33040473 |
| Nervonic acid | 1.248979564 | 0.010021737 | 0.016606016 | -0.245024517 |
| Sphingosine-1-phosphate | 1.074964643 | 0.001688657 | 0.003625168 | -0.190690614 |
| pregnenolone sulfate | 1.004095364 | 0.016305577 | 0.024490463 | -0.487610259 |
| Cellobiose | 1.05938709 | 0.01032417 | 0.017017521 | 0.449048759 |
| S-Adenosyl-L-homocysteine | 1.025885184 | 0.013414856 | 0.021027587 | 0.299951952 |
| 1-Palmitoyl Lysophosphatidic Acid | 1.584322208 | 7.79015E-06 | 4.14153E-05 | -0.232215543 |
| Bisindolylmaleimide I | 1.51119618 | 0.002220297 | 0.004596919 | -0.719730205 |
| Hydrocortisone (Cortisol) | 1.213267915 | 0.000736418 | 0.00176091 | 0.334633241 |
| Apigenin 7-glucoside | 1.052939547 | 5.87315E-06 | 3.37179E-05 | 0.451620372 |
| 1-Oleoyl-L-.alpha.-lysophosphatidic acid | 1.680788972 | 2.25311E-06 | 1.67192E-05 | -0.352107405 |
| Geranylgeranyl diphosphate (Geranyl-PP) | 1.842495689 | 1.09595E-05 | 5.51173E-05 | -0.57953543 |
| Amygdalin | 1.578506851 | 0.001767478 | 0.003767661 | 0.394588624 |
| 5,10-methylene-THF | 1.922624936 | 8.93527E-07 | 8.64097E-06 | -1.040847641 |
| Telmisartan | 1.693483422 | 5.60067E-05 | 0.000202942 | -0.953462748 |
| 1-Stearoyl-sn-glycerol 3-phosphocholine | 1.328441995 | 0.000318082 | 0.000886539 | -0.687420465 |
