# Supplementary material for: Exploration of Crucial Mediators for Carotid Atherosclerosis Pathogenesis Through Integration of Microbiome, Metabolome, and Transcriptome
Source: Front Physiol. 2021 May 24;12:645212. doi: 10.3389/fphys.2021.645212 (PMC8181762; doi:10.3389/fphys.2021.645212)
Supplement: Supplementary Table 5 — KEGG pathways for differential metabolites. [file Table_5.DOCX]

**Table S5. KEGG pathways for differential metabolites.**

| **Pathway** | **Total** | **Hits** | **Raw p** | **-ln(*p*)** | **Holm adjust** | **FDR** | **Impact** | **Hits Cpd** | **Total Cpd** |
| --- | --- | --- | --- | --- | --- | --- | --- | --- | --- |
| ***POS mode*** |  |  |  |  |  |  |  |  |  |
| Aminoacyl-tRNA biosynthesis | 75 | 7 | 0.00383 | 5.565 | 0.3065 | 0.307 | 0.056 | Glycine cpd:C00037; L-Methionine cpd:C00073; L-Valine cpd:C00183; L-Isoleucine cpd:C00407; L-Tyrosine cpd:C00082; L-Proline cpd:C00148; L-Glutamic acid cpd:C00025 | tRNA(Asn) cpd:C01637; L-Asparagine cpd:C00152; tRNA(His) cpd:C01643; L-Histidine cpd:C00135; tRNA(Phe) cpd:C01648; L-Phenylalanine cpd:C00079; L-Arginine cpd:C00062; tRNA(Arg) cpd:C01636; tRNA(Gln) cpd:C01640; L-Glutamine cpd:C00064; tRNA(Cys) cpd:C01639; L-Cysteine cpd:C00097; tRNA(Gly) cpd:C01642; Glycine cpd:C00037; tRNA(Asp) cpd:C01638; L-Aspartic acid cpd:C00049; tRNA(Ser) cpd:C01650; L-Serine cpd:C00065; L-Methionine cpd:C00073; tRNA(Met) cpd:C01647; L-Valine cpd:C00183; tRNA(Val) cpd:C01653; tRNA(Ala) cpd:C01635; L-Alanine cpd:C00041; tRNA(Lys) cpd:C01646; L-Lysine cpd:C00047; tRNA(Ile) cpd:C01644; L-Isoleucine cpd:C00407; tRNA(Leu) cpd:C01645; L-Leucine cpd:C00123; L-Threonine cpd:C00188; tRNA(Thr) cpd:C01651; tRNA(Trp) cpd:C01652; L-Tryptophan cpd:C00078; L-Methionyl-tRNA cpd:C02430; N10-Formyl-THF cpd:C00234; L-Tyrosine cpd:C00082; tRNA(Tyr) cpd:C00787; L-Proline cpd:C00148; tRNA(Pro) cpd:C01649; tRNA(Glu) cpd:C01641; L-Glutamic acid cpd:C00025; Glutaminyl-tRNA cpd:C02282; L-Asparaginyl-tRNA(Asn) cpd:C03402; O-Phosphoseryl-tRNA(Cys) cpd:C17022; Phosphoserine cpd:C01005; tRNA(Sec) cpd:C16636; L-Seryl-tRNA(Sec) cpd:C06481; O-Phosphoseryl-tRNA(Sec) cpd:C16638; L-Pyrrolysine cpd:C16138; tRNA(Pyl) cpd:C16139; L-Histidyl-tRNA(His) cpd:C02988; L-Phenylalanyl-tRNA(Phe) cpd:C03511; L-Arginyl-tRNA(Arg) cpd:C02163; L-Cysteinyl-tRNA(Cys) cpd:C03125; Glycyl-tRNA(Gly) cpd:C02412; L-Aspartyl-tRNA(Asp) cpd:C02984; L-Seryl-tRNA(Ser) cpd:C02553; L-Valyl-tRNA(Val) cpd:C02554; L-Alanyl-tRNA cpd:C00886; L-Lysyl-tRNA cpd:C01931; L-Isoleucyl-tRNA(Ile) cpd:C03127; L-Leucyl-tRNA cpd:C02047; L-Threonyl-tRNA(Thr) cpd:C02992; L-Tryptophanyl-tRNA(Trp) cpd:C03512; Tetrahydrofolic acid cpd:C00101; N-Formylmethionyl-tRNA cpd:C03294; L-Tyrosyl-tRNA(Tyr) cpd:C02839; L-Prolyl-tRNA(Pro) cpd:C02702; L-Glutamyl-tRNA(Glu) cpd:C02987; L-Glutamyl-tRNA(Gln) cpd:C06112; L-Aspartyl-tRNA(Asn) cpd:C06113; L-Selenocysteinyl-tRNA(Sec) cpd:C06482; L-Pyrrolysyl-tRNA(Pyl) cpd:C17027; L-Lysyl-tRNA(Pyl) cpd:C16140 |
| Cysteine and methionine metabolism | 56 | 5 | 0.01726 | 4.06 | 1 | 0.55 | 0.124 | 5'-Methylthioadenosine cpd:C00170; L-Methionine cpd:C00073; O-Acetylserine cpd:C00979; L-Cystine cpd:C00491; L-Homoserine cpd:C00263 | O-Succinyl-L-homoserine cpd:C01118; 1-Aminocyclopropane-1-carboxylate cpd:C01234; S-Adenosylmethionine cpd:C00019; 2-Oxo-4-methylthiobutanoic acid cpd:C01180; 2-Hydroxy-3-keto-5-methylthiopentenyl-1-phosphate cpd:C15651; 1,2-Dihydroxy-3-keto-5-methylthiopentene cpd:C15606; 2,3-Diketo-5-methylthiopentyl-1-phosphate cpd:C15650; 5-Methylthioribulose 1-phosphate cpd:C04582; 5-Methylthioribose 1-phosphate cpd:C04188; 5-Methylthioribose cpd:C03089; 5'-Methylthioadenosine cpd:C00170; S-Adenosylmethioninamine cpd:C01137; L-Methionine S-oxide cpd:C02989; DL-Homocystine cpd:C01817; L-Cystathionine cpd:C02291; N-Formyl-L-methionine cpd:C03145; L-Homocysteine cpd:C00155; L-Serine cpd:C00065; L-Methionine cpd:C00073; S-Adenosylhomocysteine cpd:C00021; S-Ribosyl-L-homocysteine cpd:C03539; O-Acetylserine cpd:C00979; Hydrogen sulfide cpd:C00283; S-Glutathionyl-L-cysteine cpd:C05526; Sulfate cpd:C00059; Glutathione cpd:C00051; L-Cysteine cpd:C00097; 2-Aminoacrylic acid cpd:C02218; Phosphoserine cpd:C01005; Cysteic acid cpd:C00506; 3-Sulfopyruvic acid cpd:C05528; 3-Sulfolactate cpd:C16069; L-Cystine cpd:C00491; 3-Sulfinoalanine cpd:C00606; 3-Sulfinylpyruvic acid cpd:C05527; D-Cysteine cpd:C00793; Sulfite cpd:C00094; 3-Mercaptopyruvic acid cpd:C00957; L-Homoserine cpd:C00263; O-Acetyl-L-homoserine cpd:C01077; L-Aspartyl-4-phosphate cpd:C03082; L-Aspartic acid cpd:C00049; Ethylene cpd:C06547; 3-Methylthiopropionic acid cpd:C08276; 2-Ketobutyric acid cpd:C00109; Methanethiol cpd:C00409; Cysteine-S-sulfate cpd:C05824; Pyruvic acid cpd:C00022; Hydrogen sulfite cpd:C11481; Thiocysteine cpd:C01962; Thiosulfate cpd:C00320; 3-Mercaptolactic acid cpd:C05823; L-Aspartate-semialdehyde cpd:C00441; Aminoacyl-L-methionine cpd:C05524; L-Alanine cpd:C00041; Sulfur dioxide cpd:C09306 |
| Nitrogen metabolism | 39 | 4 | 0.02063 | 3.881 | 1 | 0.55 | 0 | L-Tyrosine cpd:C00082; Taurine cpd:C00245; L-Glutamic acid cpd:C00025; Glycine cpd:C00037 | L-Phenylalanine cpd:C00079; L-Tyrosine cpd:C00082; Nitrite cpd:C00088; Formamide cpd:C00488; Ammonia cpd:C00014; Carbamic acid cpd:C01563; Cyanate cpd:C01417; Carbon dioxide cpd:C00011; Hydroxylamine cpd:C00192; L-Tryptophan cpd:C00078; L-threo-3-Methylaspartate cpd:C03618; alpha-Amino acid cpd:C05167; Taurine cpd:C00245; Nitrate cpd:C00244; Ethylnitronate cpd:C18091; Nitroethane cpd:C01837; Nitrogen cpd:C00697; Nitric oxide cpd:C00533; Nitrous oxide cpd:C00887; L-Aspartic acid cpd:C00049; L-Asparagine cpd:C00152; L-Glutamic acid cpd:C00025; L-Glutamine cpd:C00064; 2-Aminobenzoic acid cpd:C00108; L-Cystathionine cpd:C02291; L-Homocysteine cpd:C00155; Allocystathionine cpd:C00542; Amine cpd:C00706; Amide cpd:C00241; Cyclic amidines cpd:C06059; Amidines cpd:C06060; Nitrile cpd:C00726; L-Histidine cpd:C00135; Carbamoylphosphate cpd:C00169; Glycine cpd:C00037; Formic acid cpd:C00058; Carbonic acid cpd:C01353; Adenosine monophosphate cpd:C00020; NH4OH cpd:C01358 |
| Phenylalanine metabolism | 45 | 4 | 0.03307 | 3.409 | 1 | 0.576 | 0.173 | trans-Cinnamic acid cpd:C00423; 4-Hydroxycinnamic acid cpd:C00811; Alpha-N-Phenylacetyl-L-glutamine cpd:C04148; L-Tyrosine cpd:C00082 | 4-Hydroxy-2-oxopentanoate cpd:C03589; L-Phenylalanine cpd:C00079; Phenylacetaldehyde cpd:C00601; Phenylacetic acid cpd:C07086; Phenylacetyl-CoA cpd:C00582; 2-Hydroxy-2,4-pentadienoate cpd:C00596; 2-Hydroxy-6-oxonona-2,4-diene-1,9-dioate cpd:C04479; 2-Hydroxy-6-ketononatrienedioate cpd:C12624; 3-(2,3-Dihydroxyphenyl)propanoate cpd:C04044; trans-2,3-Dihydroxycinnamate cpd:C12623; m-Coumaric acid cpd:C12621; Phenylethylamine cpd:C05332; Phenylpyruvic acid cpd:C00166; Phenyllactate cpd:C05607; D-Phenylalanine cpd:C02265; Phenylethyl alcohol cpd:C05853; 2-Phenylacetamide cpd:C02505; trans-Cinnamic acid cpd:C00423; cis-3-(3-Carboxyethenyl)-3,5-cyclohexadiene-1,2-diol cpd:C12622; 3-(2-Hydroxyphenyl)propanoate cpd:C01198; 3-(3-Hydroxyphenyl)propanoic acid cpd:C11457; cis-3-(Carboxy-ethyl)-3,5-cyclo-hexadiene-1,2-diol cpd:C11588; Hydrocinnamic acid cpd:C05629; Benzoic acid cpd:C00180; Hippuric acid cpd:C01586; trans-2-Hydroxycinnamate cpd:C01772; 4-Hydroxycinnamic acid cpd:C00811; Phenylglyoxylic acid cpd:C02137; Phenylglyoxylyl-CoA cpd:C15524; Pyruvic acid cpd:C00022; Acetaldehyde cpd:C00084; Alpha-N-Phenylacetyl-L-glutamine cpd:C04148; Phenylacetylglycine cpd:C05598; Succinic acid cpd:C00042; Fumaric acid cpd:C00122; Ortho-Hydroxyphenylacetic acid cpd:C05852; Enol-phenylpyruvate cpd:C02763; N-Acetyl-D-phenylalanine cpd:C05620; N-Acetyl-L-phenylalanine cpd:C03519; Benzoyl-CoA cpd:C00512; 4-Hydroxybenzoic acid cpd:C00156; p-Hydroxyphenylacetic acid cpd:C00642; Salicylic acid cpd:C00805; L-Tyrosine cpd:C00082; 3-Hydroxyphenylacetic acid cpd:C05593 |
| Glycine, serine and threonine metabolism | 48 | 4 | 0.04064 | 3.203 | 1 | 0.576 | 0.317 | Betaine cpd:C00719; Dimethylglycine cpd:C01026; Glycine cpd:C00037; L-Homoserine cpd:C00263 | Betaine aldehyde cpd:C00576; L-Serine cpd:C00065; Ectoine cpd:C06231; Choline cpd:C00114; N-gamma-Acetyldiaminobutyrate cpd:C06442; L-2,4-Diaminobutanoate cpd:C03283; L-Aspartate-semialdehyde cpd:C00441; 3-Phospho-D-glycerate cpd:C00197; Glyceric acid cpd:C00258; Betaine cpd:C00719; Guanidoacetic acid cpd:C00581; Dimethylglycine cpd:C01026; L-Cystathionine cpd:C02291; Glycine cpd:C00037; L-Aspartic acid cpd:C00049; Phosphoserine cpd:C01005; Sarcosine cpd:C00213; 5,10-Methylene-THF cpd:C00143; L-Threonine cpd:C00188; O-Phosphohomoserine cpd:C01102; L-Aspartyl-4-phosphate cpd:C03082; L-Homoserine cpd:C00263; Lipoylprotein cpd:C02051; D-Serine cpd:C00740; Aminoacetone cpd:C01888; Pyruvaldehyde cpd:C00546; Tetrahydrofolic acid cpd:C00101; S-Aminomethyldihydrolipoylprotein cpd:C01242; D-Lombricine cpd:C01726; Dihydrolipoylprotein cpd:C02972; Creatine cpd:C00300; 5-Hydroxyectoine cpd:C16432; Hydroxypyruvic acid cpd:C00168; Phosphohydroxypyruvic acid cpd:C03232; L-Cysteine cpd:C00097; L-Allothreonine cpd:C05519; 2-Ketobutyric acid cpd:C00109; Glyoxylic acid cpd:C00048; L-2-Amino-3-oxobutanoic acid cpd:C03508; Pyruvic acid cpd:C00022; Carbon dioxide cpd:C00011; 5-Aminolevulinic acid cpd:C00430; Hydroxyacetone cpd:C05235; (R)-1-Aminopropan-2-ol cpd:C03194; Ammonia cpd:C00014; N-Phospho-D-lombricine cpd:C02855; PS(16:0/16:0) cpd:C02737; L-Tryptophan cpd:C00078 |
| Tyrosine metabolism | 76 | 5 | 0.05496 | 2.901 | 1 | 0.576 | 0.134 | Dopamine cpd:C03758; L-Tyrosine cpd:C00082; Tyramine cpd:C00483; Vanylglycol cpd:C05594; Acetoacetic acid cpd:C00164 | L-Dopachrome cpd:C01693; Normetanephrine cpd:C05589; 3-Methoxy-4-hydroxyphenylglycolaldehyde cpd:C05583; Norepinephrine cpd:C00547; Epinephrine cpd:C00788; 3,4-Dihydroxymandelate cpd:C05580; 3,4-Dihydroxymandelaldehyde cpd:C05577; 3,4-Dihydroxyphenylglycol cpd:C05576; Metanephrine cpd:C05588; Dopamine cpd:C03758; 3,4-Dihydroxyphenylacetaldehyde cpd:C04043; 3,4-Dihydroxybenzeneacetic acid cpd:C01161; Homovanillin cpd:C05581; 3-Methoxytyramine cpd:C05587; L-Dopa cpd:C00355; 3,5-Diiodo-L-tyrosine cpd:C01060; Iodotyrosine cpd:C02515; L-Tyrosine cpd:C00082; 3-Fumarylpyruvate cpd:C02514; Maleylpyruvate cpd:C02167; Gentisic acid cpd:C00628; Gentisate aldehyde cpd:C05585; Homogentisic acid cpd:C00544; 4-Fumarylacetoacetic acid cpd:C01061; Maleylacetoacetic acid cpd:C01036; p-Hydroxyphenylacetic acid cpd:C00642; 4-Hydroxyphenylpyruvic acid cpd:C01179; 4-Hydroxyphenylacetyl-CoA cpd:C05338; 4-Hydroxyphenylacetaldehyde cpd:C03765; Tyramine cpd:C00483; 5,6-Dihydroxyindole cpd:C05578; 3-Hydroxyphenylacetic acid cpd:C05593; 2-Hydroxy-5-carboxymethylmuconate semialdehyde cpd:C04642; 5-Carboxymethyl-2-hydroxymuconate cpd:C04186; 5-Carboxy-2-oxohept-3-enedioate cpd:C04052; 2-Hydroxyhepta-2,4-dienedioate cpd:C05600; 2,4-Dihydroxyhept-2-enedioate cpd:C06201; Succinic acid semialdehyde cpd:C00232; 3-(4-Hydroxyphenyl)lactate cpd:C03672; 4-(L-Alanin-3-yl)-2-hydroxy-cis,cis-muconate 6-semialdehyde cpd:C04796; 5-(L-Alanin-3-yl)-2-hydroxy-cis,cis-muconate 6-semialdehyde cpd:C04797; 3-(3,4-Dihydroxyphenyl)pyruvate cpd:C04045; 3-(3,4-Dihydroxyphenyl)lactic acid cpd:C01207; 2-Oxohept-3-enedioate cpd:C03063; N-Methyltyramine cpd:C02442; 4-Chlorophenylacetate cpd:C03077; Tyrosol cpd:C06044; Dopaquinone cpd:C00822; Leucodopachrome cpd:C05604; Indole-5,6-quinone cpd:C05579; 5,6-Indolequinone-2-carboxylic acid cpd:C17938; 5,6-Dihydroxyindole-2-carboxylic acid cpd:C04185; Cysteinyldopa cpd:C17935; 3,4-Dihydroxyhydrocinnamic acid cpd:C10447; Vanillylmandelic acid cpd:C05584; Vanylglycol cpd:C05594; Homovanillic acid cpd:C05582; Liothyronine cpd:C02465; Pyruvic acid cpd:C00022; Hydroquinone cpd:C00530; Fumaric acid cpd:C00122; Acetoacetic acid cpd:C00164; 2-Hydroxy-3-(4-hydroxyphenyl)propenoic acid cpd:C05350; Hydroxyphenylacetylglycine cpd:C05596; Thyroxine cpd:C01829; Phenol cpd:C00146; Beta-Tyrosine cpd:C04368; Succinic acid cpd:C00042; Stizolobate cpd:C06047; Stizolobinate cpd:C06048; Rosmarinic acid cpd:C01850; 4-Hydroxyphenylacetylglutamine cpd:C05595; Hordenine cpd:C06199; Salidroside cpd:C06046; Eumelanin cpd:C17937; Phaeomelanin cpd:C17936 |
| Lysine biosynthesis | 32 | 3 | 0.05547 | 2.892 | 1 | 0.576 | 0.109 | Diaminopimelic acid cpd:C00666; L-Homoserine cpd:C00263; Homocitric acid cpd:C01251 | Diaminopimelic acid cpd:C00666; N-Alpha-ccetyllysine cpd:C12989; N2-Acetyl-L-aminoadipate semialdehyde cpd:C12988; N2-Acetyl-L-aminoadipyl-delta-phosphate cpd:C12987; N2-Acetyl-L-aminoadipate cpd:C12986; Aminoadipic acid cpd:C00956; Oxaloglutarate cpd:C05533; UDP-N-acetylmuramoyl-L-alanyl-D-gamma-glutamyl-meso-2,6-diaminopimelate cpd:C04877; L-Aspartic acid cpd:C00049; L-Aspartyl-4-phosphate cpd:C03082; L-Homoserine cpd:C00263; L-Aspartate-semialdehyde cpd:C00441; Tetrahydrodipicolinate cpd:C03972; L-2-Amino-6-oxoheptanedioate cpd:C03871; N-Acetyl-L-2-amino-6-oxopimelate cpd:C05539; N-Succinyl-L,L-2,6-diaminopimelate cpd:C04421; N6-Acetyl-LL-2,6-diaminoheptanedioate cpd:C04390; meso-2,6-Diaminoheptanedioate cpd:C00680; Saccharopine cpd:C00449; L-Lysine cpd:C00047; alpha-Aminoadipoyl-S-acyl enzyme cpd:C05535; Homoisocitrate cpd:C05662; Homocitric acid cpd:C01251; Oxoglutaric acid cpd:C00026; Acetyl-CoA cpd:C00024; L-2-Aminoadipate adenylate cpd:C05560; Oxoadipic acid cpd:C00322; UDP-N-acetylmuramoyl-L-alanyl-D-glutamyl-6-carboxy-L-lysyl-D-alanyl-D-alanine cpd:C04882; L-2,3-Dihydrodipicolinate cpd:C03340; N-Succinyl-2-amino-6-ketopimelate cpd:C04462; L-2-Aminoadipate 6-semialdehyde cpd:C04076; (Z)-But-1-ene-1,2,4-tricarboxylate cpd:C04002 |
| Arginine and proline metabolism | 77 | 5 | 0.05757 | 2.855 | 1 | 0.576 | 0.188 | Citrulline cpd:C00327; L-Glutamic acid cpd:C00025; L-Proline cpd:C00148; Phosphocreatine cpd:C02305; Urea cpd:C00086 | L-Glutamic-gamma-semialdehyde cpd:C01165; Pyrroline hydroxycarboxylic acid cpd:C04281; L-Glutamine cpd:C00064; Ammonia cpd:C00014; Carbamoylphosphate cpd:C00169; Ornithine cpd:C00077; L-Aspartic acid cpd:C00049; Citrulline cpd:C00327; Argininosuccinic acid cpd:C03406; L-Arginine cpd:C00062; L-Glutamic acid cpd:C00025; N-Acetyl-L-alanine cpd:C00624; N-Acetyl-L-glutamyl 5-phosphate cpd:C04133; N-Acetyl-L-glutamate 5-semialdehyde cpd:C01250; N-Acetylornithine cpd:C00437; L-Proline cpd:C00148; Peptide cpd:C00012; 4-Oxoproline cpd:C01877; D-Proline cpd:C00763; 1-Pyrroline-2-carboxylic acid cpd:C03564; Hydroxyproline cpd:C01157; L-4-Hydroxyglutamate semialdehyde cpd:C05938; L-erythro-4-Hydroxyglutamate cpd:C05947; D-4-Hydroxy-2-oxoglutarate cpd:C05946; Nopaline cpd:C01682; N-(o)-Hydroxyarginine cpd:C05933; Guanidoacetic acid cpd:C00581; Creatine cpd:C00300; N-Carbamoylsarcosine cpd:C01043; Phosphocreatine cpd:C02305; N-Methylhydantoin cpd:C02565; Creatinine cpd:C00791; 5-Guanidino-2-oxopentanoate cpd:C03771; 4-Guanidinobutanal cpd:C02647; 4-Guanidinobutanoic acid cpd:C01035; Gamma-Aminobutyric acid cpd:C00334; 4-Guanidinobutanamide cpd:C03078; Agmatine cpd:C00179; N-Carbamoylputrescine cpd:C00436; N2-Succinyl-L-arginine cpd:C03296; N2-Succinyl-L-ornithine cpd:C03415; N2-Succinyl-L-glutamic acid 5-semialdehyde cpd:C05932; N-Succinyl-L-glutamate cpd:C05931; L-Glutamic acid 5-phosphate cpd:C03287; (S)-1-Pyrroline-5-carboxylate cpd:C03912; Putrescine cpd:C00134; Gamma-glutamyl-L-putrescine cpd:C15699; gamma-Glutamyl-gamma-aminobutyraldehyde cpd:C15700; 4-(Glutamylamino) butanoate cpd:C15767; 4-Aminobutyraldehyde cpd:C00555; S-Adenosylmethioninamine cpd:C01137; S-Adenosylmethionine cpd:C00019; Spermidine cpd:C00315; N-Acetylputrescine cpd:C02714; N4-Acetylaminobutanal cpd:C05936; 4-Acetamidobutanoic acid cpd:C02946; Urea cpd:C00086; Urea-1-carboxylate cpd:C01010; cis-4-Hydroxy-D-proline cpd:C03440; 1-Pyrroline-4-hydroxy-2-carboxylate cpd:C04282; Fumaric acid cpd:C00122; 5-Amino-2-oxopentanoic acid cpd:C01110; 5-Aminopentanoic acid cpd:C00431; Pyruvic acid cpd:C00022; Glyoxylic acid cpd:C00048; N2-(D-1-Carboxyethyl)-L-arginine cpd:C04137; L-Arginine phosphate cpd:C05945; Nitric oxide cpd:C00533; Sarcosine cpd:C00213; Spermine cpd:C00750; Carbon dioxide cpd:C00011; Homocarnosine cpd:C00884; Phosphoguanidinoacetate cpd:C03166; 2,5-Dioxopentanoate cpd:C00433; Pyrrole-2-carboxylic acid cpd:C05942; 2-Oxo-4-hydroxy-5-aminovalerate cpd:C05941; Linatine cpd:C05939 |
| Pyrimidine metabolism | 60 | 4 | 0.07997 | 2.526 | 1 | 0.584 | 0.021 | Cytosine cpd:C00380; 5-Methylcytosine cpd:C02376; Pseudouridine cpd:C02067; Urea cpd:C00086 | Uridine 5'-diphosphate cpd:C00015; Thioredoxin cpd:C00342; 3-Oxo-3-ureidopropanoate cpd:C15607; Uridine 5'-monophosphate cpd:C00105; dCTP cpd:C00458; dUMP cpd:C00365; L-Glutamine cpd:C00064; Carbamoylphosphate cpd:C00169; 4,5-Dihydroorotic acid cpd:C00337; Orotidylic acid cpd:C01103; RNA cpd:C00046; Uridine triphosphate cpd:C00075; Cytidine triphosphate cpd:C00063; Uridine cpd:C00299; Dihydrouracil cpd:C00429; Ureidopropionic acid cpd:C02642; CDP cpd:C00112; Cytidine monophosphate cpd:C00055; Cytidine cpd:C00475; Cytosine cpd:C00380; Uracil cpd:C00106; Barbiturate cpd:C00813; Thioredoxin disulfide cpd:C00343; dCDP cpd:C00705; dCMP cpd:C00239; Deoxycytidine cpd:C00881; Deoxyuridine triphosphate cpd:C00460; dUDP cpd:C01346; Deoxyuridine cpd:C00526; Thymidine 5'-triphosphate cpd:C00459; dTDP cpd:C00363; 5-Thymidylic acid cpd:C00364; Thymidine cpd:C00214; 5-Methylcytosine cpd:C02376; Thymine cpd:C00178; Dihydrothymine cpd:C00906; 5-Methylbarbiturate cpd:C05281; Ureidoisobutyric acid cpd:C05100; Pseudouridine cpd:C02067; Uridine diphosphate glucose cpd:C00029; 3'-UMP cpd:C01368; 2',3'-Cyclic UMP cpd:C02355; 3'-CMP cpd:C05822; 2',3'-Cyclic CMP cpd:C02354; Trimetaphosphate cpd:C02466; P1,P4-Bis(5'-uridyl) tetraphosphate cpd:C06198; 5-Hydroxymethyldeoxycytidylate cpd:C03997; 2'-Deoxy-5-hydroxymethylcytidine-5'-diphosphate cpd:C11038; Malonic acid cpd:C00383; Urea cpd:C00086; Ureidosuccinic acid cpd:C00438; Orotic acid cpd:C00295; Phosphoribosyl pyrophosphate cpd:C00119; Beta-Alanine cpd:C00099; DNA cpd:C00039; Deoxyribose 1-phosphate cpd:C00672; Methylmalonic acid cpd:C02170; 3-Aminoisobutanoic acid cpd:C05145; Pseudouridine 5'-phosphate cpd:C01168; 2'-Deoxy-5-hydroxymethylcytidine-5'-triphosphate cpd:C11039 |
| Glutathione metabolism | 38 | 3 | 0.08411 | 2.476 | 1 | 0.584 | 0.013 | Glycine cpd:C00037; L-Glutamic acid cpd:C00025; Pyroglutamic acid cpd:C01879 | Gamma-Glutamylcysteine cpd:C00669; R-S-Cysteinylglycine cpd:C05729; R-S-Glutathione cpd:C02320; Glutathione cpd:C00051; Oxidized glutathione cpd:C00127; NADP cpd:C00006; NADPH cpd:C00005; Glycine cpd:C00037; L-Cysteine cpd:C00097; L-Glutamic acid cpd:C00025; Cysteinylglycine cpd:C01419; Pyroglutamic acid cpd:C01879; L-Amino acid cpd:C00151; 5-L-Glutamyl-L-alanine cpd:C03740; S-Substituted L-cysteine cpd:C05726; Acetyl-CoA cpd:C00024; RX cpd:C01322; Spermidine cpd:C00315; Glutathionylspermidine cpd:C05730; Trypanothione cpd:C02090; Dehydroascorbate cpd:C05422; Tryparedoxin disulfide cpd:C16664; Ornithine cpd:C00077; Putrescine cpd:C00134; Spermine cpd:C00750; Glutathionylspermine cpd:C16562; Cadaverine cpd:C01672; Aminopropylcadaverine cpd:C16565; Glutathionylaminopropylcadaverine cpd:C16566; Homotrypanothione cpd:C16567; Ascorbic acid cpd:C00072; Trypanothione disulfide cpd:C03170; Tryparedoxin cpd:C16663; Bis(glutathionyl)spermine disulfide cpd:C16564; Homotrypanothione disulfide cpd:C16568; Bis-gamma-glutamylcystine cpd:C03646; S-Substituted N-acetyl-L-cysteine cpd:C05727; Bis(glutathionyl)spermine cpd:C16563 |
| Sulfur metabolism | 18 | 2 | 0.08548 | 2.459 | 1 | 0.584 | 0.077 | O-Acetylserine cpd:C00979; L-Homoserine cpd:C00263 | O-Acetylserine cpd:C00979; Hydrogen sulfide cpd:C00283; Sulfite cpd:C00094; L-Homoserine cpd:C00263; Phosphoadenosine phosphosulfate cpd:C00053; O-Succinyl-L-homoserine cpd:C01118; Adenosine phosphosulfate cpd:C00224; Thiosulfate cpd:C00320; Sulfate cpd:C00059; Trithionate cpd:C01861; L-Serine cpd:C00065; Allocystathionine cpd:C00542; Sulfide cpd:C00087; Acetic acid cpd:C00033; L-Cysteine cpd:C00097; O-Acetyl-L-homoserine cpd:C01077; Adenosine 3',5'-diphosphate cpd:C00054; L-Homocysteine cpd:C00155 |
| Valine, leucine and isoleucine degradation | 40 | 3 | 0.09482 | 2.356 | 1 | 0.584 | 0 | Acetoacetic acid cpd:C00164; L-Valine cpd:C00183; L-Isoleucine cpd:C00407 | Enzyme N6-(lipoyl)lysine cpd:C15972; 2-Methyl-1-hydroxybutyl-ThPP cpd:C15978; Enzyme N6-(dihydrolipoyl)lysine cpd:C15973; 2-Methyl-1-hydroxypropyl-ThPP cpd:C15976; 3-Methyl-1-hydroxybutyl-ThPP cpd:C15974; Acetyl-CoA cpd:C00024; Beta-Leucine cpd:C02486; L-Leucine cpd:C00123; Acetoacetyl-CoA cpd:C00332; Acetoacetic acid cpd:C00164; 3-Hydroxy-3-methylglutaryl-CoA cpd:C00356; 3-Methylcrotonyl-CoA cpd:C03069; 3-Hydroxyisovaleryl-CoA cpd:C05998; Isovaleryl-CoA cpd:C02939; Thiamine pyrophosphate cpd:C00068; 3-Methyl-2-oxovaleric acid cpd:C00671; L-Valine cpd:C00183; 2-Methylacetoacetyl-CoA cpd:C03344; (S)-3-Hydroxyisobutyrate cpd:C06001; Tiglyl-CoA cpd:C03345; Butyryl-CoA cpd:C00630; S-(2-Methylbutanoyl)-dihydrolipoamide cpd:C15979; Alpha-ketoisovaleric acid cpd:C00141; L-Isoleucine cpd:C00407; R-Methylmalonyl-CoA cpd:C01213; Methylmalonyl-CoA cpd:C00683; Propionyl-CoA cpd:C00100; (S)-Methylmalonic acid semialdehyde cpd:C06002; (S)-b-aminoisobutyric acid cpd:C03284; 2-Methyl-3-hydroxybutyryl-CoA cpd:C04405; (S)-3-Hydroxyisobutyryl-CoA cpd:C06000; Methacrylyl-CoA cpd:C03460; (S)-2-Methylbutanoyl-CoA cpd:C15980; S-(2-Methylpropionyl)-dihydrolipoamide-E cpd:C15977; 4-Methyl-2-oxopentanoate cpd:C00233; S-(3-Methylbutanoyl)-dihydrolipoamide-E cpd:C15975; beta-Ketoisocaproate cpd:C03467; 3-Methylglutaconyl-CoA cpd:C03231; Succinyl-CoA cpd:C00091; Methylmalonic acid cpd:C02170 |
| Butanoate metabolism | 40 | 3 | 0.09482 | 2.356 | 1 | 0.584 | 0.04 | Acetoacetic acid cpd:C00164; L-Glutamic acid cpd:C00025; Diacetyl cpd:C00741 | 3-Butyn-1-ol cpd:C06146; 3-Butyn-1-al cpd:C06145; 3-Butynoate cpd:C06144; (R)-3-Hydroxybutyric acid cpd:C01089; (R)-3-((R)-3-Hydroxybutanoyloxy)butanoate cpd:C04546; Acetoacetic acid cpd:C00164; 3-Hydroxy-3-methylglutaryl-CoA cpd:C00356; Acetyl-CoA cpd:C00024; Acetoacetyl-CoA cpd:C00332; (S)-3-Hydroxybutanoyl-CoA cpd:C01144; 3-Hydroxybutyryl-CoA cpd:C03561; Poly-beta-hydroxybutyrate cpd:C06143; Crotonoyl-CoA cpd:C00877; Vinylacetyl-CoA cpd:C02331; 4-Hydroxybutyric acid cpd:C00989; Gamma-Aminobutyric acid cpd:C00334; L-Glutamic acid cpd:C00025; Pyruvic acid cpd:C00022; Butanoyl-CoA cpd:C00136; Butanal cpd:C01412; Succinic acid semialdehyde cpd:C00232; Butyric acid cpd:C00246; (R)-Malate cpd:C00497; Maleic acid cpd:C01384; Succinic acid cpd:C00042; Thiamine pyrophosphate cpd:C00068; 2-(a-Hydroxyethyl)thiamine diphosphate cpd:C05125; 2-Acetolactate cpd:C00900; (S)-Acetoin cpd:C01769; (R)-Acetoin cpd:C00810; 2-Hydroxyglutaryl-CoA cpd:C03058; 2-Hydroxyglutarate cpd:C02630; Glutaconyl-1-CoA cpd:C02411; Oxoglutaric acid cpd:C00026; Butanoylphosphate cpd:C02527; 1-Butanol cpd:C06142; Fumaric acid cpd:C00122; (R,R)-Butane-2,3-diol cpd:C03044; (S,S)-Butane-2,3-diol cpd:C03046; Diacetyl cpd:C00741 |
| Purine metabolism | 92 | 5 | 0.10509 | 2.253 | 1 | 0.601 | 0.03 | Adenosine cpd:C00212; Adenine cpd:C00147; Hypoxanthine cpd:C00262; Urea cpd:C00086; Glycine cpd:C00037 | Guanosine diphosphate cpd:C00035; Xanthine cpd:C00385; Ureidoglycine cpd:C02091; Allantoic acid cpd:C00499; (S)-Ureidoglycolic acid cpd:C00603; Carbamoylphosphate cpd:C00169; D-Ribulose 5-phosphate cpd:C00117; Phosphoribosyl pyrophosphate cpd:C00119; L-Glutamine cpd:C00064; 5-Phosphoribosylamine cpd:C03090; Glycineamideribotide cpd:C03838; Phosphoribosylformylglycineamidine cpd:C04640; AICAR cpd:C04677; SAICAR cpd:C04823; 5-amino-1-(5-phospho-D-ribosyl)imidazole-4-carboxylate cpd:C04751; Phosphoribosyl formamidocarboxamide cpd:C04734; RNA cpd:C00046; Cyclic AMP cpd:C00575; Adenosine triphosphate cpd:C00002; dATP cpd:C00131; ADP cpd:C00008; dADP cpd:C00206; Adenosine monophosphate cpd:C00020; Adenylsuccinic acid cpd:C03794; Inosinic acid cpd:C00130; Adenosine cpd:C00212; Deoxyadenosine monophosphate cpd:C00360; Deoxyadenosine cpd:C00559; Deoxyinosine cpd:C05512; Xanthosine cpd:C01762; Inosine cpd:C00294; Adenine cpd:C00147; IDP cpd:C00104; Guanosine monophosphate cpd:C00144; Xanthylic acid cpd:C00655; Hypoxanthine cpd:C00262; Guanine cpd:C00242; Deoxyguanosine cpd:C00330; (S)(+)-Allantoin cpd:C02350; Uric acid cpd:C00366; Urate-3-ribonucleoside cpd:C05513; Urea cpd:C00086; Adenosine phosphosulfate cpd:C00224; 5-Hydroxyisourate cpd:C11821; Guanosine 3',5'-bis(diphosphate) cpd:C01228; Guanosine 3'-diphosphate 5'-triphosphate cpd:C04494; Guanosine triphosphate cpd:C00044; 2'-Deoxyguanosine 5'-monophosphate cpd:C00362; dGDP cpd:C00361; Guanosine cpd:C00387; dGTP cpd:C00286; Cyclic GMP cpd:C00942; 5-Aminoimidazole cpd:C05239; N-Formiminoglycine cpd:C02718; Diadenosine tetraphosphate cpd:C01260; Sulfate cpd:C00059; Phosphoadenosine phosphosulfate cpd:C00053; 5'-Phosphoribosyl-N-formylglycinamide cpd:C04376; Inosine triphosphate cpd:C00081; Inosine 5'-tetraphosphate cpd:C03614; Xanthosine 5-triphosphate cpd:C00700; 3'-AMP cpd:C01367; Guanosine 3'-phosphate cpd:C06193; Guanosine 2',3'-cyclic phosphate cpd:C06194; P1,P4-Bis(5'-xanthosyl) tetraphosphate cpd:C04392; Adenosine 2',3'-cyclic phosphate cpd:C02353; Adenosine diphosphate ribose cpd:C00301; Adenosine tetraphosphate cpd:C03483; 5-Ureido-4-imidazole carboxylate cpd:C05515; 5-Amino-4-imidazole carboxylate cpd:C05516; Imidazolone cpd:C06195; dIDP cpd:C01344; 2'-Deoxyinosine triphosphate cpd:C01345; Diadenosine triphosphate cpd:C06197; 5'-Butyrylphosphoinosine cpd:C06435; Acetyl adenylate cpd:C05993; 5'-Benzoylphosphoadenosine cpd:C06433; 5-Hydroxy-2-oxo-4-ureido-2,5-dihydro-1H-imidazole-5-carboxylate cpd:C12248; 5-Aminoimidazole ribonucleotide cpd:C03373; 5-Carboxyamino-1-(5-phospho-D-ribosyl)imidazole cpd:C15667; Oxalureate cpd:C00802; DNA cpd:C00039; Ammonia cpd:C00014; Glyoxylic acid cpd:C00048; Carbon dioxide cpd:C00011; (R)(-)-Allantoin cpd:C02348; Glycine cpd:C00037; Adenosine 3',5'-diphosphate cpd:C00054; Diguanosine tetraphosphate cpd:C01261; alpha-D-Ribose 1-phosphate cpd:C00620; dIMP cpd:C06196; 5-Amino-4-imidazolecarboxyamide cpd:C04051 |
| Primary bile acid biosynthesis | 47 | 3 | 0.13632 | 1.993 | 1 | 0.695 | 0.027 | 25-Hydroxycholesterol cpd:C15519; Glycine cpd:C00037; Taurine cpd:C00245 | Cholesterol cpd:C00187; Cholest-5-ene-3beta,26-diol cpd:C15610; 25-Hydroxycholesterol cpd:C15519; 7 alpha,26-Dihydroxy-4-cholesten-3-one cpd:C17336; 4-Cholesten-7alpha,12alpha-diol-3-one cpd:C17339; 7a-Hydroxy-cholestene-3-one cpd:C05455; 7a,12a-Dihydroxy-5b-cholestan-3-one cpd:C05453; 5-b-Cholestane-3a ,7a ,12a-triol cpd:C05454; (25R)-3alpha,7alpha,12alpha-Trihydroxy-5beta-cholestan-26-oyl-CoA cpd:C15613; (25S)-3alpha,7alpha,12alpha-Trihydroxy-5beta-cholestan-26-oyl-CoA cpd:C17343; 7a-Hydroxy-5b-cholestan-3-one cpd:C05451; 3a,7a,12a-Trihydroxy-5b-cholest-24-enoyl-CoA cpd:C05460; 3a,7a,12a-Trihydroxy-5b-24-oxocholestanoyl-CoA cpd:C05467; 3a,7a-Dihydroxy-5b-24-oxocholestanoyl-CoA cpd:C05449; Chenodeoxycholoyl-CoA cpd:C05337; Glycine cpd:C00037; Chenodeoxycholic acid glycine conjugate cpd:C05466; Taurochenodesoxycholic acid cpd:C05465; Taurine cpd:C00245; 3alpha,7alpha,12alpha,26-Tetrahydroxy-5beta-cholestane cpd:C05446; 3a,7a,12a-Trihydroxy-5b-cholestan-26-al cpd:C01301; 3 beta,7 alpha-Dihydroxy-5-cholestenoate cpd:C17335; 7-a,27-dihydroxycholesterol cpd:C06341; 7a-Hydroxycholesterol cpd:C03594; 7-a,25-Dihydroxycholesterol cpd:C15520; Choloyl-CoA cpd:C01794; Glycocholic acid cpd:C01921; Taurocholic acid cpd:C05122; 24-Hydroxycholesterol cpd:C13550; 3alpha,7alpha-Dihydroxy-5beta-cholestanate cpd:C04554; 3a,7a-Dihydroxy-5b-cholestane cpd:C05452; 3 alpha,7 alpha,26-Trihydroxy-5beta-cholestane cpd:C05444; 3a,7a-Dihydroxy-5b-cholestan-26-al cpd:C05445; (25R)-3alpha,7alpha-Dihydroxy-5beta-cholestanoyl-CoA cpd:C17345; (25S)-3alpha,7alpha-Dihydroxy-5beta-cholestanoyl-CoA cpd:C17346; 3a,7a-Dihydroxy-5b-cholest-24-enoyl-CoA cpd:C05447; 3a,7a,12a-Trihydroxy-5b-cholestanoic acid cpd:C04722; 3a,7a,12a-Trihydroxy-5b-cholestanoyl-CoA cpd:C05448; 3a,7a,12a,24-Tetrahydroxy-5b-cholestanoyl-CoA cpd:C05450; 3 beta-Hydroxy-5-cholestenoate cpd:C17333; (24S)-Cholest-5-ene-3beta,7alpha,24-triol cpd:C15518; 7 alpha-Hydroxy-3-oxo-4-cholestenoate cpd:C17337; Chenodeoxycholic acid cpd:C02528; 7alpha,25-Dihydroxy-4-cholesten-3-one cpd:C17332; Cholic acid cpd:C00695; 5b-Cyprinol sulfate cpd:C05468; 7 alpha,24-Dihydroxy-4-cholesten-3-one cpd:C17331 |
| Thiamine metabolism | 24 | 2 | 0.13907 | 1.973 | 1 | 0.695 | 0 | L-Tyrosine cpd:C00082; Glycine cpd:C00037 | L-Tyrosine cpd:C00082; Iminoglycine cpd:C15809; C15815 cpd:C15815; 1-Deoxy-D-xylulose 5-phosphate cpd:C11437; Glycine cpd:C00037; C15814 cpd:C15814; [Enzyme]-S-sulfanylcysteine cpd:C15812; C15813 cpd:C15813; [Enzyme]-cysteine cpd:C15811; L-Cysteine cpd:C00097; C15810 cpd:C15810; 5-Aminoimidazole ribonucleotide cpd:C03373; Thiamine cpd:C00378; Thiamine monophosphate cpd:C01081; Thiamin triphosphate cpd:C03028; Thiamine pyrophosphate cpd:C00068; 4-Methyl-5-(2-phosphoethyl)-thiazole cpd:C04327; 2-Methyl-4-amino-5-hydroxymethylpyrimidine diphosphate cpd:C04752; 5-(2-Hydroxyethyl)-4-methylthiazole cpd:C04294; 4-Amino-2-methyl-5-phosphomethylpyrimidine cpd:C04556; 4-Amino-5-hydroxymethyl-2-methylpyrimidine cpd:C01279; Thiamine aldehyde cpd:C05856; Heteropyrithiamine cpd:C02691; Thiamine acetic acid cpd:C02892 |
| Synthesis and degradation of ketone bodies | 6 | 1 | 0.1538 | 1.872 | 1 | 0.708 | 0.7 | Acetoacetic acid cpd:C00164 | Acetyl-CoA cpd:C00024; Acetoacetyl-CoA cpd:C00332; 3-Hydroxy-3-methylglutaryl-CoA cpd:C00356; Acetoacetic acid cpd:C00164; (R)-3-Hydroxybutyric acid cpd:C01089; Acetone cpd:C00207 |
| Valine, leucine and isoleucine biosynthesis | 27 | 2 | 0.16805 | 1.784 | 1 | 0.708 | 0.027 | L-Valine cpd:C00183; L-Isoleucine cpd:C00407 | (R)-2-Methylmalate cpd:C02612; Pyruvic acid cpd:C00022; Acetyl-CoA cpd:C00024; Citraconic acid cpd:C02226; D-erythro-3-Methylmalate cpd:C06032; L-Threonine cpd:C00188; 3-Methyl-2-oxovaleric acid cpd:C00671; L-Leucine cpd:C00123; (R) 2,3-Dihydroxy-3-methylvalerate cpd:C06007; 2-Isopropylmalic acid cpd:C02504; 3-Isopropylmalate cpd:C04411; Alpha-ketoisovaleric acid cpd:C00141; L-Valine cpd:C00183; (R)-2,3-Dihydroxy-isovalerate cpd:C04272; (S)-2-Aceto-2-hydroxybutanoic acid cpd:C06006; (S)-2-Acetolactate cpd:C06010; 2-(a-Hydroxyethyl)thiamine diphosphate cpd:C05125; L-Isoleucine cpd:C00407; 2-Ketobutyric acid cpd:C00109; 2-Isopropyl-3-oxosuccinate cpd:C04236; L-Leucyl-tRNA cpd:C02047; 4-Methyl-2-oxopentanoate cpd:C00233; (R)-3-Hydroxy-3-methyl-2-oxopentanoate cpd:C14463; Isopropylmaleate cpd:C02631; L-Valyl-tRNA(Val) cpd:C02554; 3-Hydroxy-3-methyl-2-oxobutanoic acid cpd:C04181; L-Isoleucyl-tRNA(Ile) cpd:C03127 |
| Pantothenate and CoA biosynthesis | 27 | 2 | 0.16805 | 1.784 | 1 | 0.708 | 0.18 | Pantothenic acid cpd:C00864; L-Valine cpd:C00183 | Dephospho-CoA cpd:C00882; Coenzyme A cpd:C00010; Apo-[acyl-carrier-protein] cpd:C03688; Pantetheine 4'-phosphate cpd:C01134; Acyl-carrier protein cpd:C00229; Pantetheine cpd:C00831; 4-Phosphopantothenoylcysteine cpd:C04352; D-Pantothenoyl-L-cysteine cpd:C04079; D-4'-Phosphopantothenate cpd:C03492; L-Cysteine cpd:C00097; Pantothenic acid cpd:C00864; Ureidopropionic acid cpd:C02642; Dihydrouracil cpd:C00429; (R)-Pantoate cpd:C00522; Beta-Alanine cpd:C00099; Alpha-ketoisovaleric acid cpd:C00141; L-Valine cpd:C00183; 2,3-Dihydroxy-3-methylbutanoate cpd:C04039; 2-Acetolactate cpd:C00900; Pyruvic acid cpd:C00022; (R)-4-Dehydropantoate cpd:C01053; (R)-3,3-Dimethylmalate cpd:C01088; Pantothenol cpd:C05944; L-Aspartic acid cpd:C00049; Adenosine 3',5'-diphosphate cpd:C00054; Uracil cpd:C00106; 2-Dehydropantoate cpd:C00966 |
| Pentose phosphate pathway | 32 | 2 | 0.21824 | 1.522 | 1 | 0.845 | 0.164 | 2-Keto-3-deoxy-6-phosphogluconic acid cpd:C04442; Gluconic acid cpd:C00257 | Ribose 1,5-bisphosphate cpd:C01151; Glucose 6-phosphate cpd:C00668; 2-Keto-3-deoxy-6-phosphogluconic acid cpd:C04442; Deoxyribose cpd:C01801; Deoxyribose 1-phosphate cpd:C00672; Deoxyribose 5-phosphate cpd:C00673; D-Ribulose 5-phosphate cpd:C00117; alpha-D-Ribose 1-phosphate cpd:C00620; D-Ribose cpd:C00121; Sedoheptulose 7-phosphate cpd:C05382; D-Glyceraldehyde 3-phosphate cpd:C00118; D-Ribulose 5-phosphate cpd:C00199; Beta-D-Fructose 6-phosphate cpd:C05345; beta-D-Fructose 1,6-bisphosphate cpd:C05378; 6-Phosphogluconic acid cpd:C00345; 2-Keto-D-gluconic acid cpd:C06473; 6-Phosphonoglucono-D-lactone cpd:C01236; Beta-D-Glucose 6-phosphate cpd:C01172; 2-Amino-2-deoxy-D-gluconate cpd:C03752; 2-Dehydro-3-deoxy-D-gluconate cpd:C00204; Gluconic acid cpd:C00257; Gluconolactone cpd:C00198; D-Glucose cpd:C00031; Beta-D-Glucose cpd:C00221; D-Glyceraldehyde cpd:C00577; Glyceric acid cpd:C00258; Xylulose 5-phosphate cpd:C00231; Phosphoribosyl pyrophosphate cpd:C00119; Pyruvic acid cpd:C00022; D-Erythrose 4-phosphate cpd:C00279; 6-Phospho-2-dehydro-D-gluconate cpd:C01218; 2-Phospho-D-glyceric acid cpd:C00631 |
| One carbon pool by folate | 9 | 1 | 0.2217 | 1.506 | 1 | 0.845 | 0 | Folic acid cpd:C00504 | 5,10-Methylene-THF cpd:C00143; Tetrahydrofolic acid cpd:C00101; 5-Methyltetrahydrofolic acid cpd:C00440; 5-Formiminotetrahydrofolic acid cpd:C00664; 5,10-Methenyltetrahydrofolic acid cpd:C00445; N5-Formyl-H4F cpd:C03479; N10-Formyl-THF cpd:C00234; Dihydrofolic acid cpd:C00415; Folic acid cpd:C00504 |
| Methane metabolism | 34 | 2 | 0.23872 | 1.433 | 1 | 0.845 | 0 | Glycine cpd:C00037; Trimethylamine N-oxide cpd:C01104 | 5,10-Methylenetetrahydromethanopterin cpd:C04377; Formaldehyde cpd:C00067; 5,6,7,8-Tetrahydromethanopterin cpd:C01217; 5-Formyl-5,6,7,8-tetrahydromethanopterin cpd:C01274; Methanofuran cpd:C00862; 5,10-Methenyltetrahydromethanopterin cpd:C04330; S-Formylglutathione cpd:C01031; 5,10-Methylene-THF cpd:C00143; Glycine cpd:C00037; H+ cpd:C00080; Hydrogen cpd:C00282; Carbon dioxide cpd:C00011; Formic acid cpd:C00058; S-(Hydroxymethyl)glutathione cpd:C14180; Trimethylamine cpd:C00565; Trimethylamine N-oxide cpd:C01104; Dimethylamine cpd:C00543; Methylamine cpd:C00218; N-Methyl-L-glutamate cpd:C01046; Methanol cpd:C00132; Methane cpd:C01438; Xylulose 5-phosphate cpd:C00231; 5-Methyltetrahydrofolic acid cpd:C00440; Formylmethanofuran cpd:C01001; D-arabino-Hex-3-ulose 6-phosphate cpd:C06019; D-Ribulose 5-phosphate cpd:C00199; Methylcorrinoid cpd:C06020; Carbon monoxide cpd:C00237; L-Serine cpd:C00065; Dihydroxyacetone cpd:C00184; D-Glyceraldehyde 3-phosphate cpd:C00118; Fructose 6-phosphate cpd:C00085; Corrinoid cpd:C06021; Acetyl-CoA cpd:C00024 |
| Propanoate metabolism | 35 | 2 | 0.249 | 1.39 | 1 | 0.845 | 0.028 | Acetoacetic acid cpd:C00164; L-Valine cpd:C00183 | Propanoyl phosphate cpd:C02876; 2-Ketobutyric acid cpd:C00109; Propionic acid cpd:C00163; 2-Methylcitrate cpd:C02225; cis-2-Methylaconitate cpd:C04225; (S)-Methylmalonic acid semialdehyde cpd:C06002; Methylmalonic acid cpd:C02170; Methylmalonyl-CoA cpd:C00683; Propionyl-CoA cpd:C00100; Propinol adenylate cpd:C05983; R-Methylmalonyl-CoA cpd:C01213; Succinic acid cpd:C00042; Methylisocitric acid cpd:C04593; Lactyl-CoA cpd:C00827; L-Lactic acid cpd:C00186; Hydroxypropionic acid cpd:C01013; Malonic semialdehyde cpd:C00222; Beta-Alanine cpd:C00099; 2-Propyn-1-ol cpd:C05986; 2-Propyn-1-al cpd:C05985; Propan-2-ol cpd:C01845; Acetoacetic acid cpd:C00164; Acetoacetyl-CoA cpd:C00332; Acetyl-CoA cpd:C00024; Malonyl-CoA cpd:C00083; 1-Aminocyclopropane-1-carboxylate cpd:C01234; 2-Hydroxybutyric acid cpd:C05984; Acrylyl-CoA cpd:C00894; Malonyl-CoA semialdehyde cpd:C05989; Beta-Alanyl-CoA cpd:C02335; L-Valine cpd:C00183; Succinyl-CoA cpd:C00091; 3-Hydroxypropionyl-CoA cpd:C05668; Propynoic acid cpd:C00804; Acetone cpd:C00207 |
| Ubiquinone and other terpenoid-quinone biosynthesis | 36 | 2 | 0.25929 | 1.35 | 1 | 0.845 | 0.034 | L-Tyrosine cpd:C00082; 4-Hydroxycinnamic acid cpd:C00811 | 2-Succinyl-5-enolpyruvyl-6-hydroxy-3-cyclohexene-1-carboxylate cpd:C16519; 2-Succinylbenzoyl-CoA cpd:C03160; 2-Succinylbenzoate cpd:C02730; (1R,6R)-6-Hydroxy-2-succinylcyclohexa-2,4-diene-1-carboxylate cpd:C05817; Isochorismate cpd:C00885; 4-Hydroxybenzoic acid cpd:C00156; Chorismate cpd:C00251; Spirodilactone cpd:C06986; 1,4-Dihydroxy-6-naphthoate cpd:C17018; Futalosine cpd:C16999; de-Hypoxanthine futalosine cpd:C17010; Cyclic de-hypoxanthine futalosine cpd:C17017; 1,4-Dihydroxy-2-naphthoyl-CoA cpd:C15547; 4-hydroxybenzoyl-CoA cpd:C02949; 4-Hydroxyphenylpyruvic acid cpd:C01179; 2-Methyl-6-phytylquinol cpd:C15882; 2-Methyl-6-solanyl-1,4-benzoquinol cpd:C17570; L-Tyrosine cpd:C00082; 4-Hydroxycinnamic acid cpd:C00811; Demethylphylloquinone cpd:C13309; 2-Demethylmenaquinone cpd:C05818; 1,4-Dihydroxy-2-naphthoate cpd:C03657; Octaprenyl diphosphate cpd:C04146; Phytyl diphosphate cpd:C05427; p-Coumaroyl-CoA cpd:C00223; 3-(4-Hydroxyphenyl)lactate cpd:C03672; Beta-tocopherol cpd:C14152; Delta-Tocopherol cpd:C14151; 2,3-Dimethyl-5-phytylquinol cpd:C15883; gamma-Tocopherol cpd:C02483; Homogentisic acid cpd:C00544; Solanesyl-PP cpd:C04145; Menaquinone cpd:C00828; Plastoquinone-9 cpd:C10385; Vitamin K1 cpd:C02059; Alpha-Tocopherol cpd:C02477 |
| D-Glutamine and D-glutamate metabolism | 11 | 1 | 0.26397 | 1.332 | 1 | 0.845 | 0.112 | L-Glutamic acid cpd:C00025 | D-Glutamyl-peptide cpd:C02671; D-Glutamine cpd:C00819; UDP-N-acetylmuraminate cpd:C01050; D-Glutamic acid cpd:C00217; UDP-N-acetylmuramoyl-L-alanine cpd:C01212; L-Glutamic acid cpd:C00025; L-Glutamine cpd:C00064; 5-D-Glutamyl-D-glutamyl-peptide cpd:C03933; UDP-N-acetylmuramoyl-L-alanyl-D-glutamate cpd:C00692; Pyrrolidonecarboxylic acid cpd:C02237; Oxoglutaric acid cpd:C00026 |
| Glycerophospholipid metabolism | 39 | 2 | 0.2902 | 1.237 | 1 | 0.893 | 0.065 | Ethanolamine cpd:C00189; Glycerophosphocholine cpd:C00670 | Phosphatidylethanolamine cpd:C00350; Phosphatidylcholine cpd:C00157; Glycerol 3-phosphate cpd:C00093; Triethanolamine cpd:C06771; Diethanolamine cpd:C06772; Dihydroxyacetone phosphate cpd:C00111; CDP-diacylglycerol cpd:C00269; 2-Acyl-sn-glycerol 3-phosphate cpd:C03974; Acyl-CoA cpd:C00040; 2-Acyl-sn-glycero-3-phosphocholine cpd:C04233; LysoPC(18:1(9Z)) cpd:C04230; 1,2-Diacyl-sn-glycerol cpd:C00641; Citicoline cpd:C00307; Phosphorylcholine cpd:C00588; Choline cpd:C00114; Acetylcholine cpd:C01996; O-Phosphoethanolamine cpd:C00346; Ethanolamine cpd:C00189; Phosphatidylglycerol cpd:C00344; PA(16:0/16:0) cpd:C00416; PS(16:0/16:0) cpd:C02737; Glycerylphosphorylethanolamine cpd:C01233; 1-Acyl-sn-glycerol 3-phosphate cpd:C00681; CDP-Ethanolamine cpd:C00570; L-Serine-phosphoethanolamine cpd:C03872; Phosphatidylglycerophosphate cpd:C03892; Glycerophosphocholine cpd:C00670; N-Methylethanolamine phosphate cpd:C01210; 1-Acyl-sn-glycero-3-phosphoethanolamine cpd:C04438; Phosphodimethylethanolamine cpd:C13482; 2-Acyl-sn-glycero-3-phosphoethanolamine cpd:C05973; Dihydroxyacetone Phosphate Acyl Ester cpd:C03372; CDP-glycerol cpd:C00513; Phosphatidyl-N-dimethylethanolamine cpd:C04308; Phosphatidyl-N-methylethanolamine cpd:C01241; Acetaldehyde cpd:C00084; 2-Acyl-sn-glycero-3-phosphoserine cpd:C05974; 1-Phosphatidyl-D-myo-inositol cpd:C01194; Cardiolipin cpd:C05980 |
| Nicotinate and nicotinamide metabolism | 44 | 2 | 0.34134 | 1.075 | 1 | 0.975 | 0.007 | Nicotinamide ribotide cpd:C00455; Trigonelline cpd:C01004 | (R,S)-Nicotine cpd:C16150; (2R,3S)-2,3-Dimethylmalate cpd:C03652; 2,3-Dimethylmaleate cpd:C00922; Methylitaconate cpd:C02295; 2-Methyleneglutarate cpd:C02930; 2-Formylglutarate cpd:C16159; 6-Oxo-1,4,5,6-tetrahydronicotinate cpd:C04226; 6-Hydroxynicotinic acid cpd:C01020; 2,6-Dihydroxypyridine cpd:C03056; 2,6-Dihydroxypseudooxynicotine cpd:C15986; 6-Hydroxypseudooxynicotine cpd:C01297; (S)-6-Hydroxynicotine cpd:C01056; (R)-6-Hydroxynicotine cpd:C03043; L-Aspartic acid cpd:C00049; NADP cpd:C00006; Nicotinic acid adenine dinucleotide cpd:C00857; Iminoaspartic acid cpd:C05840; Dihydroxyacetone phosphate cpd:C00111; Quinolinic acid cpd:C03722; Maleic acid cpd:C01384; Maleamate cpd:C01596; 2,5-Dihydroxypyridine cpd:C01059; Nicotinic acid cpd:C00253; Nicotinic acid mononucleotide cpd:C01185; Nicotinate D-ribonucleoside cpd:C05841; Nicotinamide ribotide cpd:C00455; Niacinamide cpd:C00153; NAD cpd:C00003; Nicotinamide riboside cpd:C03150; 1-Methylnicotinamide cpd:C02918; 1-Methylpyrrolinium cpd:C06178; (S)-2-(Hydroxymethyl)glutarate cpd:C16390; 2,3,6-Trihydroxypyridine cpd:C03458; Pyruvic acid cpd:C00022; Propionic acid cpd:C00163; 2,6-Dihydroxynicotinate cpd:C15523; 4-Methylaminobutyrate cpd:C15987; Fumaric acid cpd:C00122; Trigonelline cpd:C01004; N1-Methyl-4-pyridone-3-carboxamide cpd:C05843; N1-Methyl-2-pyridone-5-carboxamide cpd:C05842; Nicotine imine cpd:C00745; Blue pigment cpd:C16152; 2,6-Dihydroxy-N-methylmyosmine cpd:C16151 |
| Histidine metabolism | 44 | 2 | 0.34134 | 1.075 | 1 | 0.975 | 0.009 | L-Glutamic acid cpd:C00025; Ergothioneine cpd:C05570 | 4-Imidazolone-5-propionic acid cpd:C03680; Formiminoglutamic acid cpd:C00439; N-Formyl-L-glutamic acid cpd:C01045; L-Glutamic acid cpd:C00025; Urocanic acid cpd:C00785; L-Histidine cpd:C00135; Anserine cpd:C01262; Carnosine cpd:C00386; N-Formimino-L-aspartate cpd:C03409; Imidazoleacetic acid cpd:C02835; Imidazole-4-acetaldehyde cpd:C05130; 1-Methylhistamine cpd:C05127; Methylimidazole acetaldehyde cpd:C05827; Histamine cpd:C00388; Phosphoribosyl-ATP cpd:C02739; L-Histidinal cpd:C01929; L-Histidinol cpd:C00860; L-Histidinol phosphate cpd:C01100; D-Erythro-imidazole-glycerol-phosphate cpd:C04666; PhosphoribosylformiminoAICAR-phosphate cpd:C04896; Phosphoribosyl-AMP cpd:C02741; Phosphoribulosylformimino-AICAR-P cpd:C04916; 1-Methylhistidine cpd:C01152; N-Formyl-L-aspartate cpd:C01044; 4-Imidazolone-5-acetate cpd:C05133; Hercynine cpd:C05575; Ergothioneine cpd:C05570; Hydantoin-5-propionic acid cpd:C05565; Formylisoglutamine cpd:C16674; 4-Oxoglutaramate cpd:C05572; Imidazol-5-yl-pyruvate cpd:C03277; Imidazoleacetic acid ribotide cpd:C04437; N-Carbamyl-L-glutamate cpd:C05829; Methylimidazoleacetic acid cpd:C05828; Phosphoribosyl pyrophosphate cpd:C00119; Imidazole acetol-phosphate cpd:C01267; AICAR cpd:C04677; L-Aspartic acid cpd:C00049; 4-(beta-Acetylaminoethyl)imidazole cpd:C05135; Thiourocanic acid cpd:C05571; Isoglutamine cpd:C16673; Oxoglutaric acid cpd:C00026; Imidazole lactate cpd:C05568; Imidazoleacetic acid riboside cpd:C05131 |
| Cyanoamino acid metabolism | 16 | 1 | 0.35998 | 1.022 | 1 | 0.991 | 0 | Glycine cpd:C00037 | alpha-Aminopropiononitrile cpd:C05714; gamma-Amino-gamma-cyanobutanoate cpd:C05715; Hydrogen cyanide cpd:C01326; L-Aspartic acid cpd:C00049; 3-Cyano-L-alanine cpd:C02512; L-Asparagine cpd:C00152; Beta-Aminopropionitrile cpd:C05670; Glycine cpd:C00037; Monocarboxylic acid amide cpd:C03620; Alanine cpd:C01401; DL-Glutamate cpd:C00302; Formamide cpd:C00488; gamma-Glutamyl-beta-cyanoalanine cpd:C05711; gamma-Glutamyl-beta-aminopropiononitrile cpd:C06114; L-Serine cpd:C00065; Carboxylate cpd:C00060 |
| Lysine degradation | 47 | 2 | 0.37152 | 0.99 | 1 | 0.991 | 0 | N6-Acetyl-L-lysine cpd:C02727; Glycine cpd:C00037 | D-Lysine cpd:C00739; 2,5-Diaminohexanoate cpd:C05161; L-Lysine cpd:C00047; delta1-Piperideine-2-carboxylate cpd:C04092; Saccharopine cpd:C00449; 4-Trimethylammoniobutanoic acid cpd:C01181; 4-Trimethylammoniobutanal cpd:C01149; N6,N6,N6-Trimethyl-L-lysine cpd:C03793; Protein N6,N6,N6-trimethyl-L-lysine cpd:C05546; Protein N6-methyl-L-lysine cpd:C05544; Protein lysine cpd:C02188; 5-Hydroxylysine cpd:C01211; Protein N6,N6-dimethyl-L-lysine cpd:C05545; Crotonoyl-CoA cpd:C00877; (S)-3-Hydroxybutanoyl-CoA cpd:C01144; Pipecolic acid cpd:C00408; N6-Acetyl-L-lysine cpd:C02727; L-2-Aminoadipate 6-semialdehyde cpd:C04076; Aminoadipic acid cpd:C00956; Oxoadipic acid cpd:C00322; Acetoacetyl-CoA cpd:C00332; Glutaryl-CoA cpd:C00527; Glutaric acid cpd:C00489; Glutarate semialdehyde cpd:C03273; 5-Aminopentanamide cpd:C00990; 5-Acetamidopentanoate cpd:C03087; 2-Keto-6-acetamidocaproate cpd:C05548; (3S)-3,6-Diaminohexanoate cpd:C01142; (3S,5S)-3,5-Diaminohexanoate cpd:C01186; N6-Acetyl-N6-hydroxy-L-lysine cpd:C03955; N6-Hydroxy-L-lysine cpd:C01028; 3-Hydroxy-N6,N6,N6-trimethyl-L-lysine cpd:C01259; S-Glutaryldihydrolipoamide cpd:C06157; N2-(D-1-Carboxyethyl)-L-lysine cpd:C04020; Cadaverine cpd:C01672; 2-Keto-6-aminocaproate cpd:C03239; 2-Amino-5-oxohexanoate cpd:C05825; Carnitine cpd:C00487; 5-(D-Galactosyloxy)-L-lysine-procollagen cpd:C04487; 5-Phosphonooxy-L-lysine cpd:C03366; (S)-2,3,4,5-Tetrahydropyridine-2-carboxylate cpd:C00450; Acetyl-CoA cpd:C00024; 5-Aminopentanoic acid cpd:C00431; (S)-5-Amino-3-oxohexanoate cpd:C03656; Aerobactin cpd:C05554; Glycine cpd:C00037; Piperideine cpd:C06181 |
| Taurine and hypotaurine metabolism | 20 | 1 | 0.42782 | 0.849 | 1 | 1 | 0.331 | Taurine cpd:C00245 | Taurine cpd:C00245; 2-Hydroxyethanesulfonate cpd:C05123; Sulfoacetaldehyde cpd:C00593; L-Alanine cpd:C00041; Acetyl-CoA cpd:C00024; Pyruvic acid cpd:C00022; Taurocyamine cpd:C01959; Cysteic acid cpd:C00506; L-Cysteine cpd:C00097; Hypotaurine cpd:C00519; Cysteamine cpd:C01678; 3-Sulfinoalanine cpd:C00606; Acetic acid cpd:C00033; Sulfite cpd:C00094; Aminoacetaldehyde cpd:C06735; Sulfoacetate cpd:C14179; Acetylphosphate cpd:C00227; N-Phosphotaurocyamine cpd:C03149; 5-L-Glutamyl-taurine cpd:C05844; Taurocholic acid cpd:C05122 |
| Pentose and glucuronate interconversions | 53 | 2 | 0.43018 | 0.844 | 1 | 1 | 0.006 | L-Arabinose cpd:C00259; 2-Keto-3-deoxy-6-phosphogluconic acid cpd:C04442 | Pectic acid cpd:C00470; 3-Dehydro-L-gulonate 6-phosphate cpd:C14899; 3-Dehydro-L-gulonate cpd:C00618; 3-Methoxy-4-hydroxyphenylglycol glucuronide cpd:C03033; D-arabino-Hex-3-ulose 6-phosphate cpd:C06019; D-Ribulose 5-phosphate cpd:C00199; L-Arabinose cpd:C00259; L-Arabitol cpd:C00532; L-Ribulose cpd:C00508; Ribitol cpd:C00474; D-Ribulose cpd:C00309; L-Ribulose 5-phosphate cpd:C01101; D-Ribitol 5-phosphate cpd:C01068; D-Arabitol cpd:C01904; D-Xylulose cpd:C00310; 2-Dehydro-3-deoxy-D-xylonate cpd:C03826; D-Xylonate cpd:C00502; L-Threo-2-pentulose cpd:C00312; D-Xylitol cpd:C00379; D-Xylose cpd:C00181; D-Xylono-1,5-lactone cpd:C02266; Gulonic acid cpd:C00800; Glucose 1-phosphate cpd:C00103; 2-Keto-3-deoxy-6-phosphogluconic acid cpd:C04442; Uridine diphosphate glucuronic acid cpd:C00167; Uridine diphosphate glucose cpd:C00029; D-Glucuronic acid cpd:C00191; D-Glucuronic acid 1-phosphate cpd:C05385; 2-Dehydro-3-deoxy-D-gluconate cpd:C00204; D-Mannonate cpd:C00514; D-Altronate cpd:C00817; D-Galacturonate cpd:C00333; 5-Keto-D-gluconate cpd:C00558; Digalacturonate cpd:C02273; Pectin cpd:C00714; 4-(4-Deoxy-alpha-D-gluc-4-enuronosyl)-D-galacturonate cpd:C06118; 5-Dehydro-4-deoxy-D-glucuronate cpd:C04053; L-Xylulose 1-phosphate cpd:C06441; 2,3-Diketo-L-gulonate cpd:C04575; L-Xylulose 5-phosphate cpd:C03291; Fructose 6-phosphate cpd:C00085; Xylulose 5-phosphate cpd:C00231; CDP-ribitol cpd:C00789; D-Lyxose cpd:C00476; Pyruvic acid cpd:C00022; L-Lyxose cpd:C01508; D-Glyceraldehyde 3-phosphate cpd:C00118; D-Fructuronate cpd:C00905; (4S)-4,6-Dihydroxy-2,5-dioxohexanoate cpd:C04349; Glycolaldehyde cpd:C00266; Dihydroxyacetone phosphate cpd:C00111; L-Xylonate cpd:C05411; L-Lyxonate cpd:C05412 |
| Riboflavin metabolism | 21 | 1 | 0.44364 | 0.813 | 1 | 1 | 0 | Dimethylbenzimidazole cpd:C03114 | D-Ribulose 5-phosphate cpd:C00199; 5-Amino-6-(5'-phosphoribitylamino)uracil cpd:C04454; FAD cpd:C00016; Flavin Mononucleotide cpd:C00061; Riboflavin cpd:C00255; 3,4-Dihydroxy-2-butanone 4-phosphate cpd:C15556; 5-Amino-6-ribitylamino uracil cpd:C04732; Oxygen cpd:C00007; Hydroquinone cpd:C00530; 6,7-Dimethyl-8-(1-D-ribityl)lumazine cpd:C04332; 5-Amino-6-(5'-phosphoribosylamino)uracil cpd:C01268; 2,5-Diamino-6-(5'-phosphoribosylamino)-4-pyrimidineone cpd:C01304; Guanosine triphosphate cpd:C00044; Dimethylbenzimidazole cpd:C03114; N1-(5-Phospho-a-D-ribosyl)-5,6-dimethylbenzimidazole cpd:C04778; Quinone cpd:C00472; Lumichrome cpd:C01727; Ribitol cpd:C00474; Water cpd:C00001; N1-(alpha-D-ribosyl)-5,6-dimethyl-benzimidazole cpd:C05775; 7-Hydroxy-6-methyl-8-ribityl lumazine cpd:C05995 |
| Selenoamino acid metabolism | 22 | 1 | 0.45903 | 0.779 | 1 | 1 | 0.005 | O-Acetylserine cpd:C00979 | Adenylylselenate cpd:C05686; Selenate cpd:C05697; Selenite cpd:C05684; L-Seryl-tRNA(Sec) cpd:C06481; Phosphoroselenoic acid cpd:C05172; Hydrogen selenide cpd:C01528; Selenocysteine cpd:C05688; O-Acetylserine cpd:C00979; Selenomethionine cpd:C05335; Se-Adenosylselenomethionine cpd:C05691; Se-Adenosylselenohomocysteine cpd:C05692; Selenohomocysteine cpd:C05698; Selenocystathionine cpd:C05699; Se-Methylselenocysteine cpd:C05689; 3'-Phosphoadenylylselenate cpd:C05696; L-Selenocysteinyl-tRNA(Sec) cpd:C06482; L-Alanine cpd:C00041; Acetic acid cpd:C00033; Se-Methylselenomethionine cpd:C05690; Methaneselenol cpd:C05703; Selenomethionyl-tRNA(Met) cpd:C05336; Gamma-Glutamyl-Se-methylselenocysteine cpd:C05695 |
| Ether lipid metabolism | 23 | 1 | 0.474 | 0.747 | 1 | 1 | 0 | Glycerophosphocholine cpd:C00670 | 1-Alkyl-sn-glycerol cpd:C02773; Dihydroxyacetone Phosphate Acyl Ester cpd:C03372; 2-Acetyl-1-alkyl-sn-glycero-3-phosphocholine cpd:C04598; 1-Alkyl-2-acetyl-sn-glycerol cpd:C03820; 1-Alkyl-2-acylglycerophosphoethanolamine cpd:C04475; 1-Alkyl-2-acylglycerol cpd:C03201; 1-(1-Alkenyl)-sn-glycero-3-phosphoethanolamine cpd:C04635; O-1-Alk-1-enyl-2-acyl-sn-glycero-3-phosphoethanolamine cpd:C04756; 2-Acyl-1-alkyl-sn-glycero-3-phosphate cpd:C05977; Glycerylphosphorylethanolamine cpd:C01233; 1-Radyl-2-acyl-sn-glycero-3-phosphocholine cpd:C05212; 1-Alkyl-sn-glycero-3-phosphate cpd:C03968; LysoPC(O-18:0) cpd:C04317; PA(P-16:0e/18:2(9Z,12Z)) cpd:C15647; 1-Alkenyl-2-acylglycerol cpd:C03454; LPA(P-16:0e/0:0) cpd:C15646; O-Alkylglycerone phosphate cpd:C03715; 1-(1-Alkenyl)-sn-glycero-3-phosphocholine cpd:C04517; 2-Acetyl-1-alkyl-sn-glycero-3-phosphate cpd:C01264; 1-O-Alkyl-2-acetyl-3-acyl-sn-glycerol cpd:C04361; Plasmenylcholine cpd:C00958; Glycerophosphocholine cpd:C00670; 1-(1-Alkenyl)-sn-glycerol cpd:C15645 |
| Alanine, aspartate and glutamate metabolism | 24 | 1 | 0.48856 | 0.716 | 1 | 1 | 0.177 | L-Glutamic acid cpd:C00025 | N-Acetyl-L-aspartic acid cpd:C01042; 2-Oxosuccinamate cpd:C02362; L-Aspartic acid cpd:C00049; L-Asparagine cpd:C00152; D-Aspartic acid cpd:C00402; Argininosuccinic acid cpd:C03406; Adenylsuccinic acid cpd:C03794; L-Alanine cpd:C00041; Pyruvic acid cpd:C00022; Ureidosuccinic acid cpd:C00438; Succinic acid semialdehyde cpd:C00232; Oxoglutaric acid cpd:C00026; L-Glutamine cpd:C00064; L-Glutamic acid cpd:C00025; Gamma-Aminobutyric acid cpd:C00334; Ammonia cpd:C00014; 2-Keto-glutaramic acid cpd:C00940; (S)-1-Pyrroline-5-carboxylate cpd:C03912; Oxalacetic acid cpd:C00036; Fumaric acid cpd:C00122; Succinic acid cpd:C00042; Carbamoylphosphate cpd:C00169; Glucosamine 6-phosphate cpd:C00352; 5-Phosphoribosylamine cpd:C03090 |
| Phenylalanine, tyrosine and tryptophan biosynthesis | 27 | 1 | 0.52991 | 0.635 | 1 | 1 | 0.007 | L-Tyrosine cpd:C00082 | Shikimic acid cpd:C00493; Quinate cpd:C00296; 5-O-(1-Carboxyvinyl)-3-phosphoshikimate cpd:C01269; Indoleglycerol phosphate cpd:C03506; Indole cpd:C00463; 1-(2-Carboxyphenylamino)-1-deoxy-D-ribulose 5-phosphate cpd:C01302; N-(5-Phospho-D-ribosyl)anthranilate cpd:C04302; Chorismate cpd:C00251; Prephenate cpd:C00254; Phenylpyruvic acid cpd:C00166; L-Phenylalanine cpd:C00079; L-Arogenate cpd:C00826; L-Tyrosine cpd:C00082; Shikimate 3-phosphate cpd:C03175; 3-Dehydroquinate cpd:C00944; 2-Dehydro-3-deoxy-D-arabino-heptonate 7-phosphate cpd:C04691; D-Erythrose 4-phosphate cpd:C00279; Phosphoenolpyruvic acid cpd:C00074; 3-Dehydroshikimate cpd:C02637; 2-Amino-3,7-dideoxy-D-threo-hept-6-ulosonic acid cpd:C16850; 6-Deoxy-5-ketofructose 1-phosphate cpd:C16848; L-Aspartate-semialdehyde cpd:C00441; L-Tryptophan cpd:C00078; 2-Aminobenzoic acid cpd:C00108; Phosphoribosyl pyrophosphate cpd:C00119; 4-Hydroxyphenylpyruvic acid cpd:C01179; Protocatechuic acid cpd:C00230 |
| beta-Alanine metabolism | 28 | 1 | 0.54294 | 0.611 | 1 | 1 | 0 | Pantothenic acid cpd:C00864 | Beta-Alanyl-CoA cpd:C02335; Acrylyl-CoA cpd:C00894; 3-Hydroxypropionyl-CoA cpd:C05668; Hydroxypropionic acid cpd:C01013; Malonic acid cpd:C00383; Malonyl-CoA cpd:C00083; Malonic semialdehyde cpd:C00222; Beta-Alanine cpd:C00099; beta-Alanyl-L-lysine cpd:C05341; L-Aspartic acid cpd:C00049; N-Acetyl-beta-alanine cpd:C01073; Spermine cpd:C00750; Spermidine cpd:C00315; 4-Aminobutyraldehyde cpd:C00555; 1,3-Diaminopropane cpd:C00986; 3-Aminopropionaldehyde cpd:C05665; Ureidopropionic acid cpd:C02642; Dihydrouracil cpd:C00429; Carnosine cpd:C00386; Anserine cpd:C01262; beta-Alanyl-L-arginine cpd:C05340; Propionyl-CoA cpd:C00100; Acetyl-CoA cpd:C00024; Propynoic acid cpd:C00804; Pantothenic acid cpd:C00864; Gamma-Aminobutyric acid cpd:C00334; Uracil cpd:C00106; L-Histidine cpd:C00135 |
| Porphyrin and chlorophyll metabolism | 104 | 3 | 0.5499 | 0.598 | 1 | 1 | 0 | Glycine cpd:C00037; Dimethylbenzimidazole cpd:C03114; L-Glutamic acid cpd:C00025 | Adenosyl cobinamide cpd:C06508; Cobinamide cpd:C05774; Cobalt-precorrin 7 cpd:C16244; Cobalt-precorrin 5B cpd:C16243; Cobalt-precorrin 5A cpd:C16242; Heme O cpd:C15672; Heme cpd:C00032; Glycine cpd:C00037; Divinylprotochlorophyllide cpd:C11831; Coproporphyrin III cpd:C03263; O-Phosphothreonine cpd:C12147; D-1-Aminopropan-2-ol O-phosphate cpd:C04122; Adenosyl cobyrinate hexaamide cpd:C06507; Chlorophyllide cpd:C02139; Phytyl diphosphate cpd:C05427; Divinyl chlorophyllide a cpd:C11832; Protochlorophyllide cpd:C02880; 13(1)-Oxo-magnesium-protoporphyrin IX 13-monomethyl ester cpd:C11830; 13(1)-Hydroxy-magnesium-protoporphyrin IX 13-monomethyl ester cpd:C11829; Magnesium protoporphyrin monomethyl ester cpd:C04536; 15,16-Dihydrobiliverdin cpd:C11630; Biliverdin cpd:C00500; Dimethylbenzimidazole cpd:C03114; N1-(5-Phospho-a-D-ribosyl)-5,6-dimethylbenzimidazole cpd:C04778; Adenosylcobinamide-GDP cpd:C06510; N1-(alpha-D-ribosyl)-5,6-dimethyl-benzimidazole cpd:C05775; Aquacobalamin cpd:C00992; Cob(II)alamin cpd:C00541; Cyanocob(III)alamin cpd:C02823; Adenosyl cobinamide phosphate cpd:C06509; Sirohydrochlorin cpd:C05778; Precorrin 3B cpd:C06406; Precorrin 3A cpd:C05772; Precorrin 5 cpd:C06416; Cobalt-precorrin 4 cpd:C11540; Protoporphyrin IX cpd:C02191; Cobalt-sirohydrochlorin cpd:C11538; Magnesium protoporphyrin cpd:C03516; Cobalt-precorrin 3 cpd:C11539; Cob(II)yrinate a,c diamide cpd:C06504; Precorrin 2 cpd:C02463; Adenosyl cobyrinic acid a,c diamide cpd:C06506; Bilirubin diglucuronide cpd:C05787; (R)-1-Aminopropan-2-ol cpd:C03194; Cob(I)yrinate a,c diamide cpd:C06505; Fe2+ cpd:C14818; Transferrin[Fe(II)]2 cpd:C03029; Cobalt-dihydro-precorrin 6 cpd:C11543; Cobyrinate cpd:C05773; Precorrin 8X cpd:C06408; Hydrogenobyrinate cpd:C06399; Bilirubin cpd:C00486; Chlorophyll a cpd:C05306; Hemoglobin cpd:C01708; Cob(I)alamin cpd:C00853; Cobalt-precorrin 6 cpd:C11542; Hydrogenobyrinate a,c diamide cpd:C06503; 5-Aminolevulinic acid cpd:C00430; Protoporphyrinogen IX cpd:C01079; Uroporphyrinogen III cpd:C01051; Hydroxymethylbilane cpd:C01024; Porphobilinogen cpd:C00931; Uroporphyrinogen I cpd:C05766; (S)-4-Amino-5-oxopentanoate cpd:C03741; L-Glutamic acid cpd:C00025; L-Glutamyl-tRNA(Glu) cpd:C02987; Precorrin 4 cpd:C06407; Precorrin 6Y cpd:C06319; Precorrin 6X cpd:C06320; Cobalt-precorrin 8 cpd:C11545; Divinylchlorophyll a cpd:C11850; Mesobilirubinogen cpd:C05790; L-Urobilinogen cpd:C05789; D-Urobilinogen cpd:C05791; Coproporphyrinogen I cpd:C05768; L-Threonine cpd:C00188; (3Z)-Phytochromobilin cpd:C05913; (3Z)-Phycoerythrobilin cpd:C05912; Cobalt-factor III cpd:C17401; Heme A cpd:C15670; (3Z)-Phycocyanobilin cpd:C05786; Adenosylcobalamin cpd:C00194; Siroheme cpd:C00748; Fe3+ cpd:C14819; Transferrin[Fe(III)]2 cpd:C03179; 4,5-Dioxopentanoate cpd:C02800; Cytochrome c cpd:C00524; Chlorophyll b cpd:C05307; Pheophytin a cpd:C05797; Peroxidase cpd:C05785; Myoglobin cpd:C05782; Cytochrome a cpd:C05783; Catalase cpd:C05784; Oxyhemoglobin cpd:C05781; L-Urobilin cpd:C05793; I-Urobilin cpd:C05794; D-Urobilin cpd:C05795; Coproporphyrin III cpd:C05770; Coproporphyrin I cpd:C05769; Uroporphyrin I cpd:C05767; Coenzyme F430 cpd:C05777; Heptanoic acid cpd:C05799; Bacterio-pheophytins cpd:C05798; Uroporphyrin III cpd:C02469 |
| Pyruvate metabolism | 32 | 1 | 0.59162 | 0.525 | 1 | 1 | 0 | Homocitric acid cpd:C01251 | 2-(a-Hydroxyethyl)thiamine diphosphate cpd:C05125; Enzyme N6-(lipoyl)lysine cpd:C15972; Phosphoenolpyruvic acid cpd:C00074; Oxalacetic acid cpd:C00036; Pyruvic acid cpd:C00022; Dihydroxyacetone phosphate cpd:C00111; Pyruvaldehyde cpd:C00546; Lactaldehyde cpd:C05999; Propylene glycol cpd:C00583; D-Lactaldehyde cpd:C00937; S-Lactoylglutathione cpd:C03451; Acetylenedicarboxylate cpd:C03248; D-Lactic acid cpd:C00256; Lactaldehyde cpd:C00424; L-Lactic acid cpd:C00186; L-Malic acid cpd:C00149; Acetyl-CoA cpd:C00024; Formic acid cpd:C00058; Acetyl adenylate cpd:C05993; Acetic acid cpd:C00033; Acetaldehyde cpd:C00084; 2-Hydroxyethylenedicarboxylate cpd:C03981; Thiamine pyrophosphate cpd:C00068; Enzyme N6-(dihydrolipoyl)lysine cpd:C15973; Acetylphosphate cpd:C00227; S-Acetyldihydrolipoamide-E cpd:C16255; Acetoacetyl-CoA cpd:C00332; 2-Isopropylmalic acid cpd:C02504; (R)-2-Ethylmalate cpd:C02488; 2-Propylmalate cpd:C05994; Homocitric acid cpd:C01251; Malonyl-CoA cpd:C00083 |
| Folate biosynthesis | 42 | 1 | 0.69208 | 0.368 | 1 | 1 | 0 | Folic acid cpd:C00504 | Chorismate cpd:C00251; Dyspropterin cpd:C03684; 4-Amino-4-deoxychorismate cpd:C11355; Coenzyme F420 cpd:C00876; Reduced coenzyme F420 cpd:C01080; 5,10-Methylenetetrahydromethanopterin cpd:C04377; 5-Methyl-5,6,7,8-tetrahydromethanopterin cpd:C04488; Mesna cpd:C03576; Coenzyme M 7-mercaptoheptanoylthreonine-phosphate heterodisulfide cpd:C04832; 5-Formyl-5,6,7,8-tetrahydromethanopterin cpd:C01274; Formylmethanofuran cpd:C01001; 5,6,7,8-Tetrahydromethanopterin cpd:C01217; Carbon dioxide cpd:C00011; Methanofuran cpd:C00862; Tetrahydrofolyl-[Glu](n) cpd:C03541; Tetrahydrofolic acid cpd:C00101; Dihydrofolic acid cpd:C00415; 7,8-Dihydropteroic acid cpd:C00921; p-Aminobenzoic acid cpd:C00568; 2-Amino-7,8-dihydro-4-hydroxy-6-(diphosphooxymethyl)pteridine cpd:C04807; 2-Amino-4-hydroxy-6-hydroxymethyl-7,8-dihydropteridine cpd:C01300; 7,8-Dihydroneopterin cpd:C04874; Dihydroneopterin phosphate cpd:C05925; Dihydroneopterin triphosphate cpd:C04895; Dihydrobiopterin cpd:C00268; 6-Lactoyltetrahydropterin cpd:C04244; 2,5-Diamino-6-(5'-triphosphoryl-3',4'-trihydroxy-2'-oxopentyl)-amino-4-oxopyrimidine cpd:C06148; 2,5-Diaminopyrimidine nucleoside triphosphate cpd:C05923; Formamidopyrimidine nucleoside triphosphate cpd:C05922; Guanosine triphosphate cpd:C00044; N-(7-Mercaptoheptanoyl)threonine 3-O-phosphate cpd:C04628; 2-(Methylthio)ethanesulfonate cpd:C03920; Folic acid cpd:C00504; 7,8-Dihydromethanopterin cpd:C05927; 5,10-Methenyltetrahydromethanopterin cpd:C04330; Tetrahydrofolyl-[Glu](2) cpd:C09332; Glycolaldehyde cpd:C00266; Tetrahydrobiopterin cpd:C00272; Methane cpd:C01438; 6-(3'-Triphosphoryl-1'-methylglyceryl)-7-methyl-7,8-dihydrobiopterin cpd:C06149; Molybdopterin cpd:C05924; Neopterin cpd:C05926 |
| Amino sugar and nucleotide sugar metabolism | 88 | 2 | 0.70423 | 0.351 | 1 | 1 | 7E-05 | L-Fucose cpd:C01019; L-Arabinose cpd:C00259 | UDP-4-dehydro-6-deoxy-D-glucose cpd:C04089; UDP-4-keto-rhamnose cpd:C17328; Uridine diphosphate glucose cpd:C00029; D-Glucuronic acid cpd:C00191; D-Glucuronic acid 1-phosphate cpd:C05385; 1-Phospho-alpha-D-galacturonate cpd:C04037; Uridine diphosphate glucuronic acid cpd:C00167; N-Acetyl-D-glucosamine cpd:C00140; N-Acetyl-D-Glucosamine 6-Phosphate cpd:C00357; N-Acetyl-alpha-D-glucosamine 1-phosphate cpd:C04501; Uridine diphosphate-N-acetylglucosamine cpd:C00043; N-Acetylmannosamine cpd:C00645; N-Acetylneuraminic acid 9-phosphate cpd:C06241; N-Acetylneuraminic acid cpd:C00270; Galactose 1-phosphate cpd:C00446; Glucose 1-phosphate cpd:C00103; UDP-L-arabinose cpd:C00935; Glucosamine 6-phosphate cpd:C00352; Mannose 6-phosphate cpd:C00275; Guanosine diphosphate mannose cpd:C00096; alpha-D-Glucosamine 1-phosphate cpd:C06156; Fructose 6-phosphate cpd:C00085; Alpha-D-Glucose cpd:C00267; D-Galactose cpd:C00124; D-Mannose 1-phosphate cpd:C00636; GDP-L-fucose cpd:C00325; Fucose 1-phosphate cpd:C02985; L-Fucose cpd:C01019; Glucosamine cpd:C00329; UDP-L-Ara4O cpd:C16155; UDP-L-Ara4N cpd:C16153; UDP-L-Ara4FN cpd:C16154; L-Arabinose cpd:C00259; Beta-L-arabinose 1-phosphate cpd:C03906; alpha-D-Xylose 1-phosphate cpd:C03737; UDP-D-galacturonate cpd:C00617; UDP-N-acetyl-D-mannosamine cpd:C01170; UDP-N-acetyl-3-(1-carboxyvinyl)-D-glucosamine cpd:C04631; N-Acetyl-D-mannosamine 6-phosphate cpd:C04257; N-Acetylmuramate cpd:C02713; N-Acetylmuramic acid 6-phosphate cpd:C16698; N-Glycolylneuraminic acid cpd:C03410; Cytidine monophosphate N-acetylneuraminic acid cpd:C00128; Ferrocytochrome b5 cpd:C00999; Ferricytochrome b5 cpd:C00996; UDP-N-acetyl-2-amino-2-deoxy-D-glucuronate cpd:C04573; CDP-glucose cpd:C00501; CDP-4-dehydro-6-deoxy-D-glucose cpd:C01219; CDP-3,6-dideoxy-D-glucose cpd:C03598; D-Glucosaminide cpd:C06023; Chitosan cpd:C00734; Chitin cpd:C00461; Chitobiose cpd:C01674; GDP-D-Rhamnose cpd:C03117; GDP-4-Dehydro-6-deoxy-D-mannose cpd:C01222; CDP-4-dehydro-3,6-dideoxy-D-glucose cpd:C04297; D-Mannose cpd:C00159; D-Glucose cpd:C00031; Glucose 6-phosphate cpd:C00668; beta-D-Fructose cpd:C02336; CDP-4-dehydro-3,6-dideoxy-D-glucose epimer cpd:C17326; Uridine diphosphate-N-acetylgalactosamine cpd:C00203; Uridine diphosphategalactose cpd:C00052; alpha-L-Arabinan cpd:C02474; 1,4-beta-D-Xylan cpd:C02352; UDP-D-Xylose cpd:C00190; UDP-L-rhamnose cpd:C02199; D-Galacturonate cpd:C00333; Beta-D-Fructose 6-phosphate cpd:C05345; Undecaprenyl phosphate alpha-L-Ara4FN cpd:C16156; UDP-6-sulfoquinovose cpd:C11521; UDP-N-acetyl-D-mannosaminouronate cpd:C06240; UDP-N-acetylmuraminate cpd:C01050; CMP-N-glycoloylneuraminate cpd:C03691; UDP-N-acetyl-D-galactosaminuronic acid cpd:C13952; UDP-L-iduronate cpd:C02330; UDP-D-apiose cpd:C01623; GDP-D-mannuronate cpd:C00976; GDP-glucose cpd:C00394; CDP-3,6-dideoxy-D-mannose cpd:C03599; GDP-6-deoxy-D-talose cpd:C02977; GDP-L-galactose cpd:C02280; GDP-L-gulose cpd:C15925; CDP-abequose cpd:C01788; CDP-ascarylose cpd:C17327; ADP-glucose cpd:C00498; Pectin cpd:C00714; D-Xylose cpd:C00181 |
| Ascorbate and aldarate metabolism | 45 | 1 | 0.71716 | 0.332 | 1 | 1 | 0 | L-Arabinose cpd:C00259 | Ascorbic acid cpd:C00072; L-Ascorbate 6-phosphate cpd:C16186; L-Galactonate cpd:C15930; L-Gulonolactone cpd:C01040; L-Galactose cpd:C01825; L-Galactose 1-phosphate cpd:C15926; GDP-L-galactose cpd:C02280; GDP-L-gulose cpd:C15925; Guanosine diphosphate mannose cpd:C00096; Myoinositol cpd:C00137; D-Glucuronic acid cpd:C00191; Uridine diphosphate glucuronic acid cpd:C00167; Uridine diphosphate glucose cpd:C00029; 3-Dehydro-L-gulonate cpd:C00618; 2,3-Diketo-L-gulonate cpd:C04575; L-Ribulose 5-phosphate cpd:C01101; 3-Dehydro-L-gulonate 6-phosphate cpd:C14899; L-Xylulose 5-phosphate cpd:C03291; Gulonic acid cpd:C00800; Pyruvic acid cpd:C00022; Tartronate semialdehyde cpd:C01146; D-Glucarate cpd:C00818; L-xylo-Hexulonolactone cpd:C03289; 5-Dehydro-4-deoxy-D-glucarate cpd:C00679; D-Galactarate cpd:C00879; 2,5-Dioxopentanoate cpd:C00433; 2-Dehydro-3-deoxy-L-arabinonate cpd:C00684; 2-Dehydro-3-deoxy-D-xylonate cpd:C03826; Arabinonic acid cpd:C00545; D-Glucurono-6,3-lactone cpd:C02670; Threonic acid cpd:C01620; L-Arabinose cpd:C00259; L-Arabinono-1,4-lactone cpd:C01114; L-Galactono-1,4-lactone cpd:C01115; Dehydroascorbate cpd:C05422; Monodehydroascorbate cpd:C01041; D-Galacturonate cpd:C00333; D-Glucuronic acid 1-phosphate cpd:C05385; L-Lyxonate cpd:C05412; Xylulose 5-phosphate cpd:C00231; 2-Dehydro-3-deoxy-D-glucarate cpd:C03921; 5-Hydroxy-2,4-dioxopentanoate cpd:C05406; Oxoglutaric acid cpd:C00026; L-Xylonate cpd:C05411; 3-Dehydro-L-threonate cpd:C03064 |
| Fructose and mannose metabolism | 48 | 1 | 0.74022 | 0.301 | 1 | 1 | 0.032 | L-Fucose cpd:C01019 | GDP-D-Rhamnose cpd:C03117; D-Fructose cpd:C00095; L-Sorbose cpd:C00247; Sorbitol cpd:C00794; Alpha-D-Glucose cpd:C00267; Levan cpd:C01355; D-Mannose cpd:C00159; Mannitol cpd:C00392; Beta-D-Fructose 2-phosphate cpd:C03267; Mannitol 1-phosphate cpd:C00644; D-Fructose 2,6-bisphosphate cpd:C00665; 1,4-b-D-Mannan cpd:C02492; Beta-D-Fructose 6-phosphate cpd:C05345; Mannose 6-phosphate cpd:C00275; Guanosine diphosphate mannose cpd:C00096; GDP-4-Dehydro-6-deoxy-D-mannose cpd:C01222; Fucose 1-phosphate cpd:C02985; D-Mannose 1-phosphate cpd:C00636; Sorbitol-6-phosphate cpd:C01096; (Alginate)n cpd:C01768; GDP-D-mannuronate cpd:C00976; Fructose 1-phosphate cpd:C01094; D-Glyceraldehyde 3-phosphate cpd:C00118; D-Glyceraldehyde cpd:C00577; beta-D-Fructose 1,6-bisphosphate cpd:C05378; Rhamnose cpd:C00507; L-Rhamnulose cpd:C00861; L-Rhamnofuranose cpd:C02431; L-Rhamnulose 1-phosphate cpd:C01131; L-Rhamnono-1,4-lactone cpd:C02991; L-Rhamnonate cpd:C01934; 2-Dehydro-3-deoxy-L-rhamnonate cpd:C03979; L-Fuculose cpd:C01721; 2-Dehydro-3-deoxy-D-fuconate cpd:C06159; L-Fuculose 1-phosphate cpd:C01099; D-Fuconate cpd:C01680; ADP-Mannose cpd:C06192; L-Fucose cpd:C01019; 2-(alpha-D-Mannosyl)-3-phosphoglycerate cpd:C11516; GDP-6-deoxy-D-talose cpd:C02977; GDP-L-fucose cpd:C00325; Oligouronide with 4-deoxy-alpha-L-erythro-hex-4-enopyranuronosyl group cpd:C05392; Dihydroxyacetone phosphate cpd:C00111; Lactaldehyde cpd:C00424; Mannan cpd:C00464; D-Lactaldehyde cpd:C00937; Sorbose 1-phosphate cpd:C02888; 2(alpha-D-Mannosyl)-D-glycerate cpd:C11544 |
| Fatty acid metabolism | 50 | 1 | 0.75456 | 0.282 | 1 | 1 | 0 | L-Palmitoylcarnitine cpd:C02990 | Palmityl-CoA cpd:C00154; Tetradecanoyl-CoA cpd:C02593; Lauroyl-CoA cpd:C01832; Decanoyl-CoA (n-C10:0CoA) cpd:C05274; Octanoyl-CoA cpd:C01944; Hexanoyl-CoA cpd:C05270; Butanoyl-CoA cpd:C00136; Acetyl-CoA cpd:C00024; (S)-3-Hydroxybutanoyl-CoA cpd:C01144; Acyl-carrier protein cpd:C00229; Long-chain fatty acid cpd:C00638; cis,cis-3,6-Dodecadienoyl-CoA cpd:C05280; (S)-Hydroxyhexanoyl-CoA cpd:C05268; (S)-Hydroxyoctanoyl-CoA cpd:C05266; (S)-Hydroxydecanoyl-CoA cpd:C05264; Reduced rubredoxin cpd:C00340; Alkane cpd:C01371; Oxidized rubredoxin cpd:C00435; (S)-3-Hydroxydodecanoyl-CoA cpd:C05262; Primary alcohol cpd:C00226; Glutaryl-CoA cpd:C00527; Glutaric acid cpd:C00489; (S)-3-Hydroxytetradecanoyl-CoA cpd:C05260; Fatty acid cpd:C00162; Aldehyde cpd:C00071; (S)-3-Hydroxyhexadecanoyl-CoA cpd:C05258; Palmitic acid cpd:C00249; Palmitaldehyde cpd:C00517; 1-Hexadecanol cpd:C00823; (2E)-Hexadecenoyl-CoA cpd:C05272; (2E)-Tetradecenoyl-CoA cpd:C05273; (2E)-Dodecenoyl-CoA cpd:C03221; (2E)-Decenoyl-CoA cpd:C05275; (2E)-Octenoyl-CoA cpd:C05276; trans-2-Hexenoyl-CoA cpd:C05271; Crotonoyl-CoA cpd:C00877; Acetoacetyl-CoA cpd:C00332; Acyl-[acyl-carrier protein] cpd:C00173; trans,cis-Lauro-2,6-dienoyl-CoA cpd:C05279; 3-Hydroxybutyryl-CoA cpd:C03561; Coenzyme A cpd:C00010; 3-Oxohexanoyl-CoA cpd:C05269; 3-Oxooctanoyl-CoA cpd:C05267; 3-Oxodecanoyl-CoA cpd:C05265; 3-Oxododecanoyl-CoA cpd:C05263; 3-Oxotetradecanoyl-CoA cpd:C05261; omega-Hydroxy fatty acid cpd:C03547; alpha-Hydroxy fatty acid cpd:C05102; 3-Oxohexadecanoyl-CoA cpd:C05259; L-Palmitoylcarnitine cpd:C02990 |
| Tryptophan metabolism | 79 | 1 | 0.89283 | 0.113 | 1 | 1 | 0 | 5-Methoxytryptamine cpd:C05659 | L-Tryptophan cpd:C00078; Indoleacrylic acid cpd:C00331; 5-Hydroxy-L-tryptophan cpd:C00643; Melatonin cpd:C01598; N-Acetylserotonin cpd:C00978; Serotonin cpd:C00780; 5-Hydroxyindoleacetic acid cpd:C05635; 5-Hydroxykynurenamine cpd:C05638; 5-Hydroxykynurenine cpd:C05651; Indole cpd:C00463; N-Acetylindoxyl cpd:C02298; L-Formylkynurenine cpd:C02700; 2,3-Dihydroxyindole cpd:C02775; 2-Aminobenzoic acid cpd:C00108; Acetoacetyl-CoA cpd:C00332; (S)-3-Hydroxybutanoyl-CoA cpd:C01144; Crotonoyl-CoA cpd:C00877; Glutaryl-CoA cpd:C00527; Oxoadipic acid cpd:C00322; 2-Aminomuconic acid semialdehyde cpd:C03824; 2-Amino-3-carboxymuconic acid semialdehyde cpd:C04409; 3-Hydroxyanthranilic acid cpd:C00632; L-Kynurenine cpd:C00328; Formylanthranilic acid cpd:C05653; L-3-Hydroxykynurenine cpd:C03227; 2-Aminophenol cpd:C01987; 3-Hydroxykynurenamine cpd:C05636; 2-Aminomuconic acid cpd:C02220; 5-(3'-Carboxy-3'-oxopropenyl)-4,6-dihydroxypicolinate cpd:C05641; 7,8-Dihydroxykynurenate cpd:C01111; 7,8-Dihydro-7,8-dihydroxykynurenate cpd:C01249; Kynurenic acid cpd:C01717; Indoleacetaldehyde cpd:C00637; 5-Hydroxy-N-formylkynurenine cpd:C05648; 5-Hydroxyindoleacetaldehyde cpd:C05634; 5-(2'-Formylethyl)-4,6-dihydroxypicolinate cpd:C05654; Xanthurenic acid cpd:C02470; Tryptamine cpd:C00398; 3-Indoleacetonitrile cpd:C02938; (Indol-3-yl)acetamide cpd:C02693; Glucobrassicin cpd:C05837; Indoleacetic acid cpd:C00954; 2-Formaminobenzoylacetate cpd:C05835; Indole-3-acetaldehyde oxime cpd:C02937; S-(Indolylmethylthiohydroximoyl)-L-cysteine cpd:C16518; Indolylmethyl-desulfoglucosinolate cpd:C16517; Indolylmethylthiohydroximate cpd:C16516; N-Hydroxyl-tryptamine cpd:C17203; 5-Hydroxyindolepyruvate cpd:C05646; Acetyl-N-formyl-5-methoxykynurenamine cpd:C05642; 6-Hydroxymelatonin cpd:C05643; 5-Methoxytryptamine cpd:C05659; Formyl-5-hydroxykynurenamine cpd:C05647; 5-Methoxyindoleacetate cpd:C05660; 4,6-Dihydroxyquinoline cpd:C05639; 4-(2-Amino-5-hydroxyphenyl)-2,4-dioxobutanoate cpd:C05652; 2-Formylaminobenzaldehyde cpd:C03574; N-Acetylisatin cpd:C02172; Indoxyl cpd:C05658; Acetyl-CoA cpd:C00024; L-Tryptophanyl-tRNA(Trp) cpd:C03512; (Indol-3-yl)glycolaldehyde cpd:C03230; Cinnavalininate cpd:C05640; 4-(2-Amino-3-hydroxyphenyl)-2,4-dioxobutanoic acid cpd:C05645; 4-(2-Aminophenyl)-2,4-dioxobutanoic acid cpd:C01252; Isophenoxazine cpd:C02161; 4,8-Dihydroxyquinoline cpd:C05637; 5-(3'-Carboxy-3'-oxopropyl)-4,6-dihydroxypicolinate cpd:C05656; 3-Methylindolepyruvate cpd:C05644; Indolelactic acid cpd:C02043; 5-(2'-Carboxyethyl)-4,6-Dihydroxypicolinate cpd:C05655; 5-Hydroxyindoleacetylglycine cpd:C05832; 3-Methoxyanthranilate cpd:C05831; 8-Methoxykynurenate cpd:C05830; Tryptophanol cpd:C00955; N-Methylserotonin cpd:C06212; N-Methyltryptamine cpd:C06213; (Z)-5-Oxohex-2-enedioate cpd:C03453; 3-Methyldioxyindole cpd:C05834 |
| ***NEG mode*** |  |  |  |  |  |  |  |  |  |
| Aminoacyl-tRNA biosynthesis | 75 | 4 | 9.74E-05 | 9.24 | 0.0077932 | 0.0078 | 0 | L-Methionine cpd:C00073; L-Valine cpd:C00183; L-Leucine cpd:C00123; L-Tryptophan cpd:C00078 | tRNA(Asn) cpd:C01637; L-Asparagine cpd:C00152; tRNA(His) cpd:C01643; L-Histidine cpd:C00135; tRNA(Phe) cpd:C01648; L-Phenylalanine cpd:C00079; L-Arginine cpd:C00062; tRNA(Arg) cpd:C01636; tRNA(Gln) cpd:C01640; L-Glutamine cpd:C00064; tRNA(Cys) cpd:C01639; L-Cysteine cpd:C00097; tRNA(Gly) cpd:C01642; Glycine cpd:C00037; tRNA(Asp) cpd:C01638; L-Aspartic acid cpd:C00049; tRNA(Ser) cpd:C01650; L-Serine cpd:C00065; L-Methionine cpd:C00073; tRNA(Met) cpd:C01647; L-Valine cpd:C00183; tRNA(Val) cpd:C01653; tRNA(Ala) cpd:C01635; L-Alanine cpd:C00041; tRNA(Lys) cpd:C01646; L-Lysine cpd:C00047; tRNA(Ile) cpd:C01644; L-Isoleucine cpd:C00407; tRNA(Leu) cpd:C01645; L-Leucine cpd:C00123; L-Threonine cpd:C00188; tRNA(Thr) cpd:C01651; tRNA(Trp) cpd:C01652; L-Tryptophan cpd:C00078; L-Methionyl-tRNA cpd:C02430; N10-Formyl-THF cpd:C00234; L-Tyrosine cpd:C00082; tRNA(Tyr) cpd:C00787; L-Proline cpd:C00148; tRNA(Pro) cpd:C01649; tRNA(Glu) cpd:C01641; L-Glutamic acid cpd:C00025; Glutaminyl-tRNA cpd:C02282; L-Asparaginyl-tRNA(Asn) cpd:C03402; O-Phosphoseryl-tRNA(Cys) cpd:C17022; Phosphoserine cpd:C01005; tRNA(Sec) cpd:C16636; L-Seryl-tRNA(Sec) cpd:C06481; O-Phosphoseryl-tRNA(Sec) cpd:C16638; L-Pyrrolysine cpd:C16138; tRNA(Pyl) cpd:C16139; L-Histidyl-tRNA(His) cpd:C02988; L-Phenylalanyl-tRNA(Phe) cpd:C03511; L-Arginyl-tRNA(Arg) cpd:C02163; L-Cysteinyl-tRNA(Cys) cpd:C03125; Glycyl-tRNA(Gly) cpd:C02412; L-Aspartyl-tRNA(Asp) cpd:C02984; L-Seryl-tRNA(Ser) cpd:C02553; L-Valyl-tRNA(Val) cpd:C02554; L-Alanyl-tRNA cpd:C00886; L-Lysyl-tRNA cpd:C01931; L-Isoleucyl-tRNA(Ile) cpd:C03127; L-Leucyl-tRNA cpd:C02047; L-Threonyl-tRNA(Thr) cpd:C02992; L-Tryptophanyl-tRNA(Trp) cpd:C03512; Tetrahydrofolic acid cpd:C00101; N-Formylmethionyl-tRNA cpd:C03294; L-Tyrosyl-tRNA(Tyr) cpd:C02839; L-Prolyl-tRNA(Pro) cpd:C02702; L-Glutamyl-tRNA(Glu) cpd:C02987; L-Glutamyl-tRNA(Gln) cpd:C06112; L-Aspartyl-tRNA(Asn) cpd:C06113; L-Selenocysteinyl-tRNA(Sec) cpd:C06482; L-Pyrrolysyl-tRNA(Pyl) cpd:C17027; L-Lysyl-tRNA(Pyl) cpd:C16140 |
| Valine, leucine and isoleucine biosynthesis | 27 | 2 | 0.00416 | 5.48 | 0.32839 | 0.155 | 0.027 | L-Leucine cpd:C00123; L-Valine cpd:C00183 | (R)-2-Methylmalate cpd:C02612; Pyruvic acid cpd:C00022; Acetyl-CoA cpd:C00024; Citraconic acid cpd:C02226; D-erythro-3-Methylmalate cpd:C06032; L-Threonine cpd:C00188; 3-Methyl-2-oxovaleric acid cpd:C00671; L-Leucine cpd:C00123; (R) 2,3-Dihydroxy-3-methylvalerate cpd:C06007; 2-Isopropylmalic acid cpd:C02504; 3-Isopropylmalate cpd:C04411; Alpha-ketoisovaleric acid cpd:C00141; L-Valine cpd:C00183; (R)-2,3-Dihydroxy-isovalerate cpd:C04272; (S)-2-Aceto-2-hydroxybutanoic acid cpd:C06006; (S)-2-Acetolactate cpd:C06010; 2-(a-Hydroxyethyl)thiamine diphosphate cpd:C05125; L-Isoleucine cpd:C00407; 2-Ketobutyric acid cpd:C00109; 2-Isopropyl-3-oxosuccinate cpd:C04236; L-Leucyl-tRNA cpd:C02047; 4-Methyl-2-oxopentanoate cpd:C00233; (R)-3-Hydroxy-3-methyl-2-oxopentanoate cpd:C14463; Isopropylmaleate cpd:C02631; L-Valyl-tRNA(Val) cpd:C02554; 3-Hydroxy-3-methyl-2-oxobutanoic acid cpd:C04181; L-Isoleucyl-tRNA(Ile) cpd:C03127 |
| Pentose phosphate pathway | 32 | 2 | 0.00582 | 5.15 | 0.45374 | 0.155 | 0 | Deoxyribose cpd:C01801; D-Ribose cpd:C00121 | Ribose 1,5-bisphosphate cpd:C01151; Glucose 6-phosphate cpd:C00668; 2-Keto-3-deoxy-6-phosphogluconic acid cpd:C04442; Deoxyribose cpd:C01801; Deoxyribose 1-phosphate cpd:C00672; Deoxyribose 5-phosphate cpd:C00673; D-Ribulose 5-phosphate cpd:C00117; alpha-D-Ribose 1-phosphate cpd:C00620; D-Ribose cpd:C00121; Sedoheptulose 7-phosphate cpd:C05382; D-Glyceraldehyde 3-phosphate cpd:C00118; D-Ribulose 5-phosphate cpd:C00199; Beta-D-Fructose 6-phosphate cpd:C05345; beta-D-Fructose 1,6-bisphosphate cpd:C05378; 6-Phosphogluconic acid cpd:C00345; 2-Keto-D-gluconic acid cpd:C06473; 6-Phosphonoglucono-D-lactone cpd:C01236; Beta-D-Glucose 6-phosphate cpd:C01172; 2-Amino-2-deoxy-D-gluconate cpd:C03752; 2-Dehydro-3-deoxy-D-gluconate cpd:C00204; Gluconic acid cpd:C00257; Gluconolactone cpd:C00198; D-Glucose cpd:C00031; Beta-D-Glucose cpd:C00221; D-Glyceraldehyde cpd:C00577; Glyceric acid cpd:C00258; Xylulose 5-phosphate cpd:C00231; Phosphoribosyl pyrophosphate cpd:C00119; Pyruvic acid cpd:C00022; D-Erythrose 4-phosphate cpd:C00279; 6-Phospho-2-dehydro-D-gluconate cpd:C01218; 2-Phospho-D-glyceric acid cpd:C00631 |
| Valine, leucine and isoleucine degradation | 40 | 2 | 0.00901 | 4.71 | 0.69351 | 0.18 | 0.022 | L-Leucine cpd:C00123; L-Valine cpd:C00183 | Enzyme N6-(lipoyl)lysine cpd:C15972; 2-Methyl-1-hydroxybutyl-ThPP cpd:C15978; Enzyme N6-(dihydrolipoyl)lysine cpd:C15973; 2-Methyl-1-hydroxypropyl-ThPP cpd:C15976; 3-Methyl-1-hydroxybutyl-ThPP cpd:C15974; Acetyl-CoA cpd:C00024; Beta-Leucine cpd:C02486; L-Leucine cpd:C00123; Acetoacetyl-CoA cpd:C00332; Acetoacetic acid cpd:C00164; 3-Hydroxy-3-methylglutaryl-CoA cpd:C00356; 3-Methylcrotonyl-CoA cpd:C03069; 3-Hydroxyisovaleryl-CoA cpd:C05998; Isovaleryl-CoA cpd:C02939; Thiamine pyrophosphate cpd:C00068; 3-Methyl-2-oxovaleric acid cpd:C00671; L-Valine cpd:C00183; 2-Methylacetoacetyl-CoA cpd:C03344; (S)-3-Hydroxyisobutyrate cpd:C06001; Tiglyl-CoA cpd:C03345; Butyryl-CoA cpd:C00630; S-(2-Methylbutanoyl)-dihydrolipoamide cpd:C15979; Alpha-ketoisovaleric acid cpd:C00141; L-Isoleucine cpd:C00407; R-Methylmalonyl-CoA cpd:C01213; Methylmalonyl-CoA cpd:C00683; Propionyl-CoA cpd:C00100; (S)-Methylmalonic acid semialdehyde cpd:C06002; (S)-b-aminoisobutyric acid cpd:C03284; 2-Methyl-3-hydroxybutyryl-CoA cpd:C04405; (S)-3-Hydroxyisobutyryl-CoA cpd:C06000; Methacrylyl-CoA cpd:C03460; (S)-2-Methylbutanoyl-CoA cpd:C15980; S-(2-Methylpropionyl)-dihydrolipoamide-E cpd:C15977; 4-Methyl-2-oxopentanoate cpd:C00233; S-(3-Methylbutanoyl)-dihydrolipoamide-E cpd:C15975; beta-Ketoisocaproate cpd:C03467; 3-Methylglutaconyl-CoA cpd:C03231; Succinyl-CoA cpd:C00091; Methylmalonic acid cpd:C02170 |
| Phenylalanine, tyrosine and tryptophan biosynthesis | 27 | 1 | 0.0967 | 2.34 | 1 | 1 | 0 | L-Tryptophan cpd:C00078 | Shikimic acid cpd:C00493; Quinate cpd:C00296; 5-O-(1-Carboxyvinyl)-3-phosphoshikimate cpd:C01269; Indoleglycerol phosphate cpd:C03506; Indole cpd:C00463; 1-(2-Carboxyphenylamino)-1-deoxy-D-ribulose 5-phosphate cpd:C01302; N-(5-Phospho-D-ribosyl)anthranilate cpd:C04302; Chorismate cpd:C00251; Prephenate cpd:C00254; Phenylpyruvic acid cpd:C00166; L-Phenylalanine cpd:C00079; L-Arogenate cpd:C00826; L-Tyrosine cpd:C00082; Shikimate 3-phosphate cpd:C03175; 3-Dehydroquinate cpd:C00944; 2-Dehydro-3-deoxy-D-arabino-heptonate 7-phosphate cpd:C04691; D-Erythrose 4-phosphate cpd:C00279; Phosphoenolpyruvic acid cpd:C00074; 3-Dehydroshikimate cpd:C02637; 2-Amino-3,7-dideoxy-D-threo-hept-6-ulosonic acid cpd:C16850; 6-Deoxy-5-ketofructose 1-phosphate cpd:C16848; L-Aspartate-semialdehyde cpd:C00441; L-Tryptophan cpd:C00078; 2-Aminobenzoic acid cpd:C00108; Phosphoribosyl pyrophosphate cpd:C00119; 4-Hydroxyphenylpyruvic acid cpd:C01179; Protocatechuic acid cpd:C00230 |
| Pantothenate and CoA biosynthesis | 27 | 1 | 0.0967 | 2.34 | 1 | 1 | 0 | L-Valine cpd:C00183 | Dephospho-CoA cpd:C00882; Coenzyme A cpd:C00010; Apo-[acyl-carrier-protein] cpd:C03688; Pantetheine 4'-phosphate cpd:C01134; Acyl-carrier protein cpd:C00229; Pantetheine cpd:C00831; 4-Phosphopantothenoylcysteine cpd:C04352; D-Pantothenoyl-L-cysteine cpd:C04079; D-4'-Phosphopantothenate cpd:C03492; L-Cysteine cpd:C00097; Pantothenic acid cpd:C00864; Ureidopropionic acid cpd:C02642; Dihydrouracil cpd:C00429; (R)-Pantoate cpd:C00522; Beta-Alanine cpd:C00099; Alpha-ketoisovaleric acid cpd:C00141; L-Valine cpd:C00183; 2,3-Dihydroxy-3-methylbutanoate cpd:C04039; 2-Acetolactate cpd:C00900; Pyruvic acid cpd:C00022; (R)-4-Dehydropantoate cpd:C01053; (R)-3,3-Dimethylmalate cpd:C01088; Pantothenol cpd:C05944; L-Aspartic acid cpd:C00049; Adenosine 3',5'-diphosphate cpd:C00054; Uracil cpd:C00106; 2-Dehydropantoate cpd:C00966 |
| Propanoate metabolism | 35 | 1 | 0.1237 | 2.09 | 1 | 1 | 0 | L-Valine cpd:C00183 | Propanoyl phosphate cpd:C02876; 2-Ketobutyric acid cpd:C00109; Propionic acid cpd:C00163; 2-Methylcitrate cpd:C02225; cis-2-Methylaconitate cpd:C04225; (S)-Methylmalonic acid semialdehyde cpd:C06002; Methylmalonic acid cpd:C02170; Methylmalonyl-CoA cpd:C00683; Propionyl-CoA cpd:C00100; Propinol adenylate cpd:C05983; R-Methylmalonyl-CoA cpd:C01213; Succinic acid cpd:C00042; Methylisocitric acid cpd:C04593; Lactyl-CoA cpd:C00827; L-Lactic acid cpd:C00186; Hydroxypropionic acid cpd:C01013; Malonic semialdehyde cpd:C00222; Beta-Alanine cpd:C00099; 2-Propyn-1-ol cpd:C05986; 2-Propyn-1-al cpd:C05985; Propan-2-ol cpd:C01845; Acetoacetic acid cpd:C00164; Acetoacetyl-CoA cpd:C00332; Acetyl-CoA cpd:C00024; Malonyl-CoA cpd:C00083; 1-Aminocyclopropane-1-carboxylate cpd:C01234; 2-Hydroxybutyric acid cpd:C05984; Acrylyl-CoA cpd:C00894; Malonyl-CoA semialdehyde cpd:C05989; Beta-Alanyl-CoA cpd:C02335; L-Valine cpd:C00183; Succinyl-CoA cpd:C00091; 3-Hydroxypropionyl-CoA cpd:C05668; Propynoic acid cpd:C00804; Acetone cpd:C00207 |
| Nitrogen metabolism | 39 | 1 | 0.13694 | 1.99 | 1 | 1 | 0 | L-Tryptophan cpd:C00078 | L-Phenylalanine cpd:C00079; L-Tyrosine cpd:C00082; Nitrite cpd:C00088; Formamide cpd:C00488; Ammonia cpd:C00014; Carbamic acid cpd:C01563; Cyanate cpd:C01417; Carbon dioxide cpd:C00011; Hydroxylamine cpd:C00192; L-Tryptophan cpd:C00078; L-threo-3-Methylaspartate cpd:C03618; alpha-Amino acid cpd:C05167; Taurine cpd:C00245; Nitrate cpd:C00244; Ethylnitronate cpd:C18091; Nitroethane cpd:C01837; Nitrogen cpd:C00697; Nitric oxide cpd:C00533; Nitrous oxide cpd:C00887; L-Aspartic acid cpd:C00049; L-Asparagine cpd:C00152; L-Glutamic acid cpd:C00025; L-Glutamine cpd:C00064; 2-Aminobenzoic acid cpd:C00108; L-Cystathionine cpd:C02291; L-Homocysteine cpd:C00155; Allocystathionine cpd:C00542; Amine cpd:C00706; Amide cpd:C00241; Cyclic amidines cpd:C06059; Amidines cpd:C06060; Nitrile cpd:C00726; L-Histidine cpd:C00135; Carbamoylphosphate cpd:C00169; Glycine cpd:C00037; Formic acid cpd:C00058; Carbonic acid cpd:C01353; Adenosine monophosphate cpd:C00020; NH4OH cpd:C01358 |
| Glycine, serine and threonine metabolism | 48 | 1 | 0.16606 | 1.8 | 1 | 1 | 0 | L-Tryptophan cpd:C00078 | Betaine aldehyde cpd:C00576; L-Serine cpd:C00065; Ectoine cpd:C06231; Choline cpd:C00114; N-gamma-Acetyldiaminobutyrate cpd:C06442; L-2,4-Diaminobutanoate cpd:C03283; L-Aspartate-semialdehyde cpd:C00441; 3-Phospho-D-glycerate cpd:C00197; Glyceric acid cpd:C00258; Betaine cpd:C00719; Guanidoacetic acid cpd:C00581; Dimethylglycine cpd:C01026; L-Cystathionine cpd:C02291; Glycine cpd:C00037; L-Aspartic acid cpd:C00049; Phosphoserine cpd:C01005; Sarcosine cpd:C00213; 5,10-Methylene-THF cpd:C00143; L-Threonine cpd:C00188; O-Phosphohomoserine cpd:C01102; L-Aspartyl-4-phosphate cpd:C03082; L-Homoserine cpd:C00263; Lipoylprotein cpd:C02051; D-Serine cpd:C00740; Aminoacetone cpd:C01888; Pyruvaldehyde cpd:C00546; Tetrahydrofolic acid cpd:C00101; S-Aminomethyldihydrolipoylprotein cpd:C01242; D-Lombricine cpd:C01726; Dihydrolipoylprotein cpd:C02972; Creatine cpd:C00300; 5-Hydroxyectoine cpd:C16432; Hydroxypyruvic acid cpd:C00168; Phosphohydroxypyruvic acid cpd:C03232; L-Cysteine cpd:C00097; L-Allothreonine cpd:C05519; 2-Ketobutyric acid cpd:C00109; Glyoxylic acid cpd:C00048; L-2-Amino-3-oxobutanoic acid cpd:C03508; Pyruvic acid cpd:C00022; Carbon dioxide cpd:C00011; 5-Aminolevulinic acid cpd:C00430; Hydroxyacetone cpd:C05235; (R)-1-Aminopropan-2-ol cpd:C03194; Ammonia cpd:C00014; N-Phospho-D-lombricine cpd:C02855; PS(16:0/16:0) cpd:C02737; L-Tryptophan cpd:C00078 |
| Cysteine and methionine metabolism | 56 | 1 | 0.19121 | 1.65 | 1 | 1 | 0.038 | L-Methionine cpd:C00073 | O-Succinyl-L-homoserine cpd:C01118; 1-Aminocyclopropane-1-carboxylate cpd:C01234; S-Adenosylmethionine cpd:C00019; 2-Oxo-4-methylthiobutanoic acid cpd:C01180; 2-Hydroxy-3-keto-5-methylthiopentenyl-1-phosphate cpd:C15651; 1,2-Dihydroxy-3-keto-5-methylthiopentene cpd:C15606; 2,3-Diketo-5-methylthiopentyl-1-phosphate cpd:C15650; 5-Methylthioribulose 1-phosphate cpd:C04582; 5-Methylthioribose 1-phosphate cpd:C04188; 5-Methylthioribose cpd:C03089; 5'-Methylthioadenosine cpd:C00170; S-Adenosylmethioninamine cpd:C01137; L-Methionine S-oxide cpd:C02989; DL-Homocystine cpd:C01817; L-Cystathionine cpd:C02291; N-Formyl-L-methionine cpd:C03145; L-Homocysteine cpd:C00155; L-Serine cpd:C00065; L-Methionine cpd:C00073; S-Adenosylhomocysteine cpd:C00021; S-Ribosyl-L-homocysteine cpd:C03539; O-Acetylserine cpd:C00979; Hydrogen sulfide cpd:C00283; S-Glutathionyl-L-cysteine cpd:C05526; Sulfate cpd:C00059; Glutathione cpd:C00051; L-Cysteine cpd:C00097; 2-Aminoacrylic acid cpd:C02218; Phosphoserine cpd:C01005; Cysteic acid cpd:C00506; 3-Sulfopyruvic acid cpd:C05528; 3-Sulfolactate cpd:C16069; L-Cystine cpd:C00491; 3-Sulfinoalanine cpd:C00606; 3-Sulfinylpyruvic acid cpd:C05527; D-Cysteine cpd:C00793; Sulfite cpd:C00094; 3-Mercaptopyruvic acid cpd:C00957; L-Homoserine cpd:C00263; O-Acetyl-L-homoserine cpd:C01077; L-Aspartyl-4-phosphate cpd:C03082; L-Aspartic acid cpd:C00049; Ethylene cpd:C06547; 3-Methylthiopropionic acid cpd:C08276; 2-Ketobutyric acid cpd:C00109; Methanethiol cpd:C00409; Cysteine-S-sulfate cpd:C05824; Pyruvic acid cpd:C00022; Hydrogen sulfite cpd:C11481; Thiocysteine cpd:C01962; Thiosulfate cpd:C00320; 3-Mercaptolactic acid cpd:C05823; L-Aspartate-semialdehyde cpd:C00441; Aminoacyl-L-methionine cpd:C05524; L-Alanine cpd:C00041; Sulfur dioxide cpd:C09306 |
| Tryptophan metabolism | 79 | 1 | 0.25981 | 1.35 | 1 | 1 | 0.109 | L-Tryptophan cpd:C00078 | L-Tryptophan cpd:C00078; Indoleacrylic acid cpd:C00331; 5-Hydroxy-L-tryptophan cpd:C00643; Melatonin cpd:C01598; N-Acetylserotonin cpd:C00978; Serotonin cpd:C00780; 5-Hydroxyindoleacetic acid cpd:C05635; 5-Hydroxykynurenamine cpd:C05638; 5-Hydroxykynurenine cpd:C05651; Indole cpd:C00463; N-Acetylindoxyl cpd:C02298; L-Formylkynurenine cpd:C02700; 2,3-Dihydroxyindole cpd:C02775; 2-Aminobenzoic acid cpd:C00108; Acetoacetyl-CoA cpd:C00332; (S)-3-Hydroxybutanoyl-CoA cpd:C01144; Crotonoyl-CoA cpd:C00877; Glutaryl-CoA cpd:C00527; Oxoadipic acid cpd:C00322; 2-Aminomuconic acid semialdehyde cpd:C03824; 2-Amino-3-carboxymuconic acid semialdehyde cpd:C04409; 3-Hydroxyanthranilic acid cpd:C00632; L-Kynurenine cpd:C00328; Formylanthranilic acid cpd:C05653; L-3-Hydroxykynurenine cpd:C03227; 2-Aminophenol cpd:C01987; 3-Hydroxykynurenamine cpd:C05636; 2-Aminomuconic acid cpd:C02220; 5-(3'-Carboxy-3'-oxopropenyl)-4,6-dihydroxypicolinate cpd:C05641; 7,8-Dihydroxykynurenate cpd:C01111; 7,8-Dihydro-7,8-dihydroxykynurenate cpd:C01249; Kynurenic acid cpd:C01717; Indoleacetaldehyde cpd:C00637; 5-Hydroxy-N-formylkynurenine cpd:C05648; 5-Hydroxyindoleacetaldehyde cpd:C05634; 5-(2'-Formylethyl)-4,6-dihydroxypicolinate cpd:C05654; Xanthurenic acid cpd:C02470; Tryptamine cpd:C00398; 3-Indoleacetonitrile cpd:C02938; (Indol-3-yl)acetamide cpd:C02693; Glucobrassicin cpd:C05837; Indoleacetic acid cpd:C00954; 2-Formaminobenzoylacetate cpd:C05835; Indole-3-acetaldehyde oxime cpd:C02937; S-(Indolylmethylthiohydroximoyl)-L-cysteine cpd:C16518; Indolylmethyl-desulfoglucosinolate cpd:C16517; Indolylmethylthiohydroximate cpd:C16516; N-Hydroxyl-tryptamine cpd:C17203; 5-Hydroxyindolepyruvate cpd:C05646; Acetyl-N-formyl-5-methoxykynurenamine cpd:C05642; 6-Hydroxymelatonin cpd:C05643; 5-Methoxytryptamine cpd:C05659; Formyl-5-hydroxykynurenamine cpd:C05647; 5-Methoxyindoleacetate cpd:C05660; 4,6-Dihydroxyquinoline cpd:C05639; 4-(2-Amino-5-hydroxyphenyl)-2,4-dioxobutanoate cpd:C05652; 2-Formylaminobenzaldehyde cpd:C03574; N-Acetylisatin cpd:C02172; Indoxyl cpd:C05658; Acetyl-CoA cpd:C00024; L-Tryptophanyl-tRNA(Trp) cpd:C03512; (Indol-3-yl)glycolaldehyde cpd:C03230; Cinnavalininate cpd:C05640; 4-(2-Amino-3-hydroxyphenyl)-2,4-dioxobutanoic acid cpd:C05645; 4-(2-Aminophenyl)-2,4-dioxobutanoic acid cpd:C01252; Isophenoxazine cpd:C02161; 4,8-Dihydroxyquinoline cpd:C05637; 5-(3'-Carboxy-3'-oxopropyl)-4,6-dihydroxypicolinate cpd:C05656; 3-Methylindolepyruvate cpd:C05644; Indolelactic acid cpd:C02043; 5-(2'-Carboxyethyl)-4,6-Dihydroxypicolinate cpd:C05655; 5-Hydroxyindoleacetylglycine cpd:C05832; 3-Methoxyanthranilate cpd:C05831; 8-Methoxykynurenate cpd:C05830; Tryptophanol cpd:C00955; N-Methylserotonin cpd:C06212; N-Methyltryptamine cpd:C06213; (Z)-5-Oxohex-2-enedioate cpd:C03453; 3-Methyldioxyindole cpd:C05834 |
