# Supplementary material for: Exploration of Crucial Mediators for Carotid Atherosclerosis Pathogenesis Through Integration of Microbiome, Metabolome, and Transcriptome
Source: Front Physiol. 2021 May 24;12:645212. doi: 10.3389/fphys.2021.645212 (PMC8181762; doi:10.3389/fphys.2021.645212)
Supplement: Supplementary Table 6 — Covariates adjustment for differentially enriched genera between CAS patients and healthy controls using GLM analysis. [file Table_6.DOCX]

**Table S6. Co-variates adjustment for differentially enriched genera between CAS patients and healthy controls using GLM analysis.**

|  |  | **Estimate** | **Std. Error** | **z value** | **Pr(>\|z\|)** | **5%** | **95%** |
| --- | --- | --- | --- | --- | --- | --- | --- |
| g__Acidaminococcus | (Intercept) | 2.20795578 | 4.9567 | 0.44544874 | 0.65599549 | -5.94509019 | 10.3610018 |
|  | Age | 0.00926793 | 0.072282 | 0.12821905 | 0.89797562 | -0.10962538 | 0.12816124 |
|  | Sex | -0.90200558 | 1.6912 | -0.5333524 | 0.59378966 | -3.68378203 | 1.87977088 |
| g__Anaerostipes | (Intercept) | 2.84833017 | 2.3379 | 1.21832849 | 0.22309917 | -0.99717313 | 6.69383346 |
|  | Age | 0.02677426 | 0.035932 | 0.7451368 | 0.45618903 | -0.03232863 | 0.08587714 |
|  | Sex | -1.4045783 | 0.62231 | -2.25703958 | 0.0240056 | -2.42818716 | -0.38096944 |
| g__Christensenella | (Intercept) | -18.0978522 | 69.558 | -0.26018362 | 0.79472214 | -132.510581 | 96.3148764 |
|  | Age | 0.11933404 | 0.17836 | 0.66906278 | 0.50345543 | -0.17404206 | 0.41271013 |
|  | Sex | 9.17236225 | 68.62 | 0.13366893 | 0.89366437 | -103.697494 | 122.042218 |
| g__Clostridium_XlVa | (Intercept) | 8.79239661 | 1.2479 | 7.04575416 | 1.8446E-12 | 6.73978377 | 10.8450095 |
|  | Age | -0.03793017 | 0.019044 | -1.99171229 | 0.04640263 | -0.06925476 | -0.00660558 |
|  | Sex | -0.69996958 | 0.32378 | -2.16186786 | 0.03062836 | -1.23254028 | -0.16739887 |
| g__Clostridium_XlVb | (Intercept) | 2.86533146 | 1.8328 | 1.56336287 | 0.11796727 | -0.14935627 | 5.88001919 |
|  | Age | 0.01778517 | 0.028903 | 0.61533989 | 0.53833028 | -0.02975604 | 0.06532637 |
|  | Sex | -0.17768966 | 0.43806 | -0.4056286 | 0.68501551 | -0.89823424 | 0.54285492 |
| g__Clostridium_XVIII | (Intercept) | 6.46427529 | 2.5461 | 2.53889293 | 0.01112038 | 2.27631347 | 10.6522371 |
|  | Age | -0.05282829 | 0.039358 | -1.34225044 | 0.17951481 | -0.11756644 | 0.01190986 |
|  | Sex | -1.70701588 | 0.62124 | -2.74775591 | 0.00600047 | -2.72886475 | -0.68516701 |
| g__Fusobacterium | (Intercept) | -1.92659396 | 11.017 | -0.17487465 | 0.86117813 | -20.0479464 | 16.1947584 |
|  | Age | 0.10153656 | 0.17306 | 0.58671303 | 0.55739645 | -0.18312181 | 0.38619493 |
|  | Sex | -2.14837376 | 1.7585 | -1.22170814 | 0.22181802 | -5.04084886 | 0.74410134 |
| g__Gemella | (Intercept) | -136.199722 | 11.017 | -12.3626869 | 4.1601E-35 | -154.321074 | -118.078369 |
|  | Age | 0.01013485 | 0.17306 | 0.05856264 | 0.95330047 | -0.27452352 | 0.29479322 |
|  | Sex | 132.893361 | 1.7585 | 75.5719993 | 0 | 130.000886 | 135.785836 |
| g__Lactobacillus | (Intercept) | -1.60786386 | 6.186 | -0.2599198 | 0.79492564 | -11.7829284 | 8.56720067 |
|  | Age | 0.00352226 | 0.095308 | 0.03695658 | 0.97051963 | -0.15324545 | 0.16028997 |
|  | Sex | 5.11062866 | 1.6257 | 3.14364807 | 0.00166856 | 2.43659012 | 7.7846672 |
| g__Parvimonas | (Intercept) | -79.3450794 | 6.186 | -12.8265566 | 1.1642E-37 | -89.520144 | -69.1700149 |
|  | Age | -0.26988358 | 0.095308 | -2.83169914 | 0.00463014 | -0.42665129 | -0.11311587 |
|  | Sex | 94.0057096 | 1.6257 | 57.8247583 | 0 | 91.3316711 | 96.6797482 |
| g__Romboutsia | (Intercept) | 2.12218006 | 2.9691 | 0.71475533 | 0.47476024 | -2.76155485 | 7.00591496 |
|  | Age | 0.00323214 | 0.045893 | 0.07042773 | 0.94385322 | -0.07225513 | 0.07871941 |
|  | Sex | 0.25906261 | 0.63399 | 0.40862254 | 0.68281669 | -0.78375815 | 1.30188336 |
