# Supplementary material for: Exploration of Crucial Mediators for Carotid Atherosclerosis Pathogenesis Through Integration of Microbiome, Metabolome, and Transcriptome
Source: Front Physiol. 2021 May 24;12:645212. doi: 10.3389/fphys.2021.645212 (PMC8181762; doi:10.3389/fphys.2021.645212)
Supplement: Supplementary Table 7 — Covariates adjustment for differential metabolites between CAS patients and healthy controls using PERMANOVA analysis. [file Table_7.DOCX]

**Table S7. Co-variates adjustment for differential metabolites between CAS patients and healthy controls using PERMANOVA analysis.**

| **Phenotype** | **Df** | **SumsOfSqs** | **MeanSqs** | **F.Model** | **R2** | **Pr(>F)** |
| --- | --- | --- | --- | --- | --- | --- |
| ***POS*** |  |  |  |  |  |  |
| Ethanolamine | 1 | 80.725596 | 80.725596 | 2.3626441 | 0.0367083 | 0.1365 |
| Gly-Pro | 1 | 98.2351553 | 98.235155 | 2.8990691 | 0.0446704 | 0.1006 |
| Propoxur | 1 | 98.9959604 | 98.99596 | 2.92258 | 0.0450164 | 0.0986 |
| Homocitrate | 1 | 41.1342984 | 41.134298 | 1.1818146 | 0.018705 | 0.2568 |
| Alpha-N-Phenylacetyl-L-glutamine | 1 | 3.72816207 | 3.7281621 | 0.1052874 | 0.0016953 | 0.7461 |
| Diethylcarbamazine | 1 | 4.68992767 | 4.6899277 | 0.1325068 | 0.0021326 | 0.7168 |
| Dimethylbenzimidazole | 1 | 13.4380443 | 13.438044 | 0.3811912 | 0.0061107 | 0.5374 |
| Eicosapentaenoic acid | 1 | 4.38046356 | 4.3804636 | 0.1237459 | 0.0019919 | 0.7265 |
| Decanoyl-L-carnitine | 1 | 106.531619 | 106.53162 | 3.1563751 | 0.0484431 | 0.082 |
| 3-Methoxy-4-Hydroxyphenylglycol Sulfate | 1 | 4.23624081 | 4.2362408 | 0.1196638 | 0.0019263 | 0.731 |
| O-Desmethylnaproxen | 1 | 83.5462895 | 83.54629 | 2.4484592 | 0.037991 | 0.1264 |
| ***NEG*** |  |  |  |  |  |  |
| Salicylic acid | 1 | 34.8569208 | 34.856921 | 0.9985568 | 0.0158505 | 0.3025 |
| 3-Aminopropanesulphonic Acid | 1 | 38.9061337 | 38.906134 | 1.1166451 | 0.0176918 | 0.2734 |
| 6-Hydroxynicotinic acid | 1 | 41.2307502 | 41.23075 | 1.1846387 | 0.0187488 | 0.2607 |
| Formylanthranilic acid | 1 | 52.041702 | 52.041702 | 1.5027871 | 0.0236649 | 0.2201 |
| Xanthopterin | 1 | 55.8962186 | 55.896219 | 1.6169953 | 0.0254177 | 0.2135 |
| N1-Methyl-4-pyridone-3-carboxamide | 1 | 32.5650053 | 32.565005 | 0.9319128 | 0.0148083 | 0.3466 |
| 3-Hydroxydodecanoic acid | 1 | 29.2955327 | 29.295533 | 0.837087 | 0.0133215 | 0.3703 |
| Salicyluric acid | 1 | 24.9730038 | 24.973004 | 0.7121569 | 0.011356 | 0.3833 |
| Phenylacetylglycine | 1 | 6.54031574 | 6.5403157 | 0.1849427 | 0.0029741 | 0.677 |
| D-Biotin | 1 | 39.2434527 | 39.243453 | 1.1265024 | 0.0178452 | 0.3006 |
| Alpha-N-Phenylacetyl-L-glutamine | 1 | 5.22016733 | 5.2201673 | 0.1475236 | 0.0023738 | 0.7037 |
| 5,10-methylene-THF | 1 | 49.9908055 | 49.990806 | 1.4421866 | 0.0227323 | 0.2362 |
