# Supplementary material for: Exploration of Crucial Mediators for Carotid Atherosclerosis Pathogenesis Through Integration of Microbiome, Metabolome, and Transcriptome
Source: Front Physiol. 2021 May 24;12:645212. doi: 10.3389/fphys.2021.645212 (PMC8181762; doi:10.3389/fphys.2021.645212)
Supplement: Supplementary Table 8 — Functional enrichment analysis for DEGs. [file Table_8.DOCX]

**Table S8. Functional enrichment analysis for DEGs.**

| **Category** | **Term** | **Count** | ***p*-value** | **Genes** | **FDR** |
| --- | --- | --- | --- | --- | --- |
| ***GO-BP*** |  |  |  |  |  |
| GOTERM_BP_DIRECT | GO:0006955~immune response | 16 | 2.39E-08 | CCR1, SBSPON, ADAMDEC1, IL1RN, IGHV1OR15-1, AQP9, IGHV4-59, IGKV3D-11, IGKV1D-33, CXCL10, IGKV2D-28, C7, IGKC, RGS1, CD36, IGHV3-7 | 1.74E-05 |
| GOTERM_BP_DIRECT | GO:0006958~complement activation, classical pathway | 9 | 1.21E-07 | CR1, IGKV2D-28, C7, IGKC, IGHV4-59, IGKV3D-11, IGKV1D-33, IGHV3-7, C2 | 4.40E-05 |
| GOTERM_BP_DIRECT | GO:0006956~complement activation | 8 | 7.94E-07 | IGKV2D-28, C7, IGKC, IGHV4-59, IGKV3D-11, IGKV1D-33, IGHV3-7, C2 | 1.93E-04 |
| GOTERM_BP_DIRECT | GO:0006508~proteolysis | 15 | 1.34E-06 | ADAMDEC1, MMP7, MME, IGHV4-59, IGKV3D-11, IGKV1D-33, MMP8, MMP9, C2, MMP12, DPP4, IGKV2D-28, IGKC, ANPEP, IGHV3-7 | 2.45E-04 |
| GOTERM_BP_DIRECT | GO:0006898~receptor-mediated endocytosis | 9 | 1.41E-05 | SBSPON, CD163, IGKV2D-28, IGKC, IGHV4-59, IGKV3D-11, CD36, IGKV1D-33, IGHV3-7 | 0.0020629 |
| GOTERM_BP_DIRECT | GO:0038096~Fc-gamma receptor signaling pathway involved in phagocytosis | 7 | 1.05E-04 | VAV3, IGKV2D-28, IGKC, IGHV4-59, IGKV3D-11, IGKV1D-33, IGHV3-7 | 0.0127577 |
| GOTERM_BP_DIRECT | GO:0038095~Fc-epsilon receptor signaling pathway | 7 | 6.47E-04 | VAV3, IGKV2D-28, IGKC, IGHV4-59, IGKV3D-11, IGKV1D-33, IGHV3-7 | 0.0646788 |
| GOTERM_BP_DIRECT | GO:0019441~tryptophan catabolic process to kynurenine | 3 | 7.09E-04 | TDO2, KYNU, KMO | 0.0646788 |
| GOTERM_BP_DIRECT | GO:0006569~tryptophan catabolic process | 3 | 0.0012058 | TDO2, KYNU, KMO | 0.0978023 |
| GOTERM_BP_DIRECT | GO:0007155~cell adhesion | 10 | 0.0015501 | CCR1, NLGN1, MMRN1, IBSP, ITGAX, CNTN1, CNTN3, CD36, CNTN4, SELE | 0.1131559 |
| GOTERM_BP_DIRECT | GO:0007229~integrin-mediated signaling pathway | 5 | 0.0027889 | VAV3, ADAMDEC1, IBSP, PLEK, ITGAX | 0.1850842 |
| GOTERM_BP_DIRECT | GO:0090026~positive regulation of monocyte chemotaxis | 3 | 0.0039126 | CCR1, CXCL10, PLA2G7 | 0.2277434 |
| GOTERM_BP_DIRECT | GO:0050776~regulation of immune response | 6 | 0.0040557 | IGKV2D-28, IGKC, IGHV4-59, IGKV3D-11, IGKV1D-33, IGHV3-7 | 0.2277434 |
| GOTERM_BP_DIRECT | GO:1903779~regulation of cardiac conduction | 4 | 0.0043945 | RYR2, PLN, NPR1, CASQ2 | 0.2291395 |
| GOTERM_BP_DIRECT | GO:0010881~regulation of cardiac muscle contraction by regulation of the release of sequestered calcium ion | 3 | 0.0055117 | RYR2, PLN, CASQ2 | 0.2682379 |
| GOTERM_BP_DIRECT | GO:0030574~collagen catabolic process | 4 | 0.0063849 | MMP12, MMP7, MMP8, MMP9 | 0.286226 |
| GOTERM_BP_DIRECT | GO:0008217~regulation of blood pressure | 4 | 0.0066655 | NPR1, NPR3, NPY1R, HMOX1 | 0.286226 |
| GOTERM_BP_DIRECT | GO:0022617~extracellular matrix disassembly | 4 | 0.0102405 | MMP12, MMP7, MMP8, MMP9 | 0.4153102 |
| GOTERM_BP_DIRECT | GO:0086029~Purkinje myocyte to ventricular cardiac muscle cell signaling | 2 | 0.0117569 | RYR2, CASQ2 | 0.4291276 |
| GOTERM_BP_DIRECT | GO:0019442~tryptophan catabolic process to acetyl-CoA | 2 | 0.0117569 | TDO2, KYNU | 0.4291276 |
| GOTERM_BP_DIRECT | GO:0048662~negative regulation of smooth muscle cell proliferation | 3 | 0.0125956 | NPR1, NPR3, HMOX1 | 0.4378477 |
| GOTERM_BP_DIRECT | GO:0030449~regulation of complement activation | 3 | 0.013444 | CR1, C7, C2 | 0.4460948 |
| GOTERM_BP_DIRECT | GO:2000721~positive regulation of transcription from RNA polymerase II promoter involved in smooth muscle cell differentiation | 2 | 0.017584 | MYOCD, NPNT | 0.458439 |
| GOTERM_BP_DIRECT | GO:0015855~pyrimidine nucleobase transport | 2 | 0.017584 | AQP9, SLC28A3 | 0.458439 |
| GOTERM_BP_DIRECT | GO:0043420~anthranilate metabolic process | 2 | 0.017584 | KYNU, KMO | 0.458439 |
| GOTERM_BP_DIRECT | GO:1904823~purine nucleobase transmembrane transport | 2 | 0.017584 | AQP9, SLC28A3 | 0.458439 |
| GOTERM_BP_DIRECT | GO:0072531~pyrimidine-containing compound transmembrane transport | 2 | 0.017584 | AQP9, SLC28A3 | 0.458439 |
| GOTERM_BP_DIRECT | GO:0019805~quinolinate biosynthetic process | 2 | 0.017584 | KYNU, KMO | 0.458439 |
| GOTERM_BP_DIRECT | GO:0007631~feeding behavior | 3 | 0.0210753 | NEGR1, NPY1R, MRAP2 | 0.5181386 |
| GOTERM_BP_DIRECT | GO:0031175~neuron projection development | 4 | 0.0212934 | LGI1, NLGN1, CNTN1, CNTN4 | 0.5181386 |
| GOTERM_BP_DIRECT | GO:0034354~'de novo' NAD biosynthetic process from tryptophan | 2 | 0.023377 | KYNU, KMO | 0.5504905 |
| GOTERM_BP_DIRECT | GO:0071356~cellular response to tumor necrosis factor | 4 | 0.0272551 | DCSTAMP, FABP4, CHI3L1, NPNT | 0.6028133 |
| GOTERM_BP_DIRECT | GO:0007399~nervous system development | 6 | 0.0274485 | TTLL7, LGI1, NLGN1, CNTN3, CNTN4, SCRG1 | 0.6028133 |
| GOTERM_BP_DIRECT | GO:0060048~cardiac muscle contraction | 3 | 0.028902 | RYR2, ACTC1, CASQ2 | 0.6028133 |
| GOTERM_BP_DIRECT | GO:0008015~blood circulation | 3 | 0.028902 | CXCL10, PLN, NPY1R | 0.6028133 |
| GOTERM_BP_DIRECT | GO:0007193~adenylate cyclase-inhibiting G-protein coupled receptor signaling pathway | 3 | 0.0313208 | RGS1, NPR3, NPY1R | 0.6351153 |
| GOTERM_BP_DIRECT | GO:0034220~ion transmembrane transport | 5 | 0.0356792 | GRIA1, RYR2, GRIA2, CASQ2, ATP6V0D2 | 0.7039411 |
| GOTERM_BP_DIRECT | GO:1904706~negative regulation of vascular smooth muscle cell proliferation | 2 | 0.0462129 | CNN1, HMOX1 | 0.8433853 |
| GOTERM_BP_DIRECT | GO:0034383~low-density lipoprotein particle clearance | 2 | 0.0462129 | HMOX1, CD36 | 0.8433853 |
| GOTERM_BP_DIRECT | GO:0071313~cellular response to caffeine | 2 | 0.0462129 | RYR2, CASQ2 | 0.8433853 |
| ***KEGG*** |  |  |  |  |  |
| KEGG_PATHWAY | hsa04024:cAMP signaling pathway | 7 | 0.0014944 | VAV3, GRIA1, RYR2, GRIA2, PLN, NPR1, NPY1R | 0.1237424 |
| KEGG_PATHWAY | hsa00380:Tryptophan metabolism | 4 | 0.0020453 | TPH1, TDO2, KYNU, KMO | 0.1237424 |
| KEGG_PATHWAY | hsa04720:Long-term potentiation | 4 | 0.0084405 | GRIA1, GRIA2, RPS6KA6, PLCB4 | 0.2661143 |
| KEGG_PATHWAY | hsa03320:PPAR signaling pathway | 4 | 0.0087972 | FABP4, ACADL, FABP5, CD36 | 0.2661143 |
| KEGG_PATHWAY | hsa04640:Hematopoietic cell lineage | 4 | 0.0178192 | CR1, MME, ANPEP, CD36 | 0.4312258 |
| KEGG_PATHWAY | hsa04713:Circadian entrainment | 4 | 0.0224642 | GRIA1, RYR2, GRIA2, PLCB4 | 0.453029 |
| KEGG_PATHWAY | hsa05144:Malaria | 3 | 0.0388915 | CR1, CD36, SELE | 0.6722669 |
| KEGG_PATHWAY | hsa04923:Regulation of lipolysis in adipocytes | 3 | 0.049525 | FABP4, NPR1, NPY1R | 0.6790625 |
