# Supplementary material for: Exploration of Crucial Mediators for Carotid Atherosclerosis Pathogenesis Through Integration of Microbiome, Metabolome, and Transcriptome
Source: Front Physiol. 2021 May 24;12:645212. doi: 10.3389/fphys.2021.645212 (PMC8181762; doi:10.3389/fphys.2021.645212)
Supplement: Supplementary Table 9 — Enrichment analysis on the DEGs using the Reactome database. [file Table_9.DOCX]

**Table S9. Enrichment analysis on the DEGs using the Reactome database.**

| **Pathways** | **Count** | ***p*-value** | **Genes** | **FDR** |
| --- | --- | --- | --- | --- |
| Scavenging of heme from plasma | 8 | 3.80E-07 | CD163, IGKV2D-28, IGHV1OR15-1, IGKC, IGHV4-59, IGKV3D-11, IGKV1D-33, IGHV3-7 | 2.81E-05 |
| Initial triggering of complement | 8 | 6.77E-07 | IGKV2D-28, IGHV1OR15-1, IGKC, IGHV4-59, IGKV3D-11, IGKV1D-33, IGHV3-7, C2 | 2.81E-05 |
| FCERI mediated MAPK activation | 8 | 1.87E-06 | VAV3, IGKV2D-28, IGHV1OR15-1, IGKC, IGHV4-59, IGKV3D-11, IGKV1D-33, IGHV3-7 | 4.19E-05 |
| FCERI mediated Ca+2 mobilization | 8 | 2.02E-06 | VAV3, IGKV2D-28, IGHV1OR15-1, IGKC, IGHV4-59, IGKV3D-11, IGKV1D-33, IGHV3-7 | 4.19E-05 |
| CD22 mediated BCR regulation | 7 | 3.29E-06 | IGKV2D-28, IGHV1OR15-1, IGKC, IGHV4-59, IGKV3D-11, IGKV1D-33, IGHV3-7 | 4.55E-05 |
| Fc epsilon receptor (FCERI) signaling | 7 | 3.29E-06 | IGKV2D-28, IGHV1OR15-1, IGKC, IGHV4-59, IGKV3D-11, IGKV1D-33, IGHV3-7 | 4.55E-05 |
| Classical antibody-mediated complement activation | 7 | 4.37E-06 | IGKV2D-28, IGHV1OR15-1, IGKC, IGHV4-59, IGKV3D-11, IGKV1D-33, IGHV3-7 | 5.19E-05 |
| FCGR activation | 7 | 7.41E-06 | IGKV2D-28, IGHV1OR15-1, IGKC, IGHV4-59, IGKV3D-11, IGKV1D-33, IGHV3-7 | 7.43E-05 |
| Role of LAT2/NTAL/LAB on calcium mobilization | 7 | 8.05E-06 | IGKV2D-28, IGHV1OR15-1, IGKC, IGHV4-59, IGKV3D-11, IGKV1D-33, IGHV3-7 | 7.43E-05 |
| Regulation of actin dynamics for phagocytic cup formation | 8 | 1.43E-05 | VAV3, IGKV2D-28, IGHV1OR15-1, IGKC, IGHV4-59, IGKV3D-11, IGKV1D-33, IGHV3-7 | 1.19E-04 |
| Role of phospholipids in phagocytosis | 7 | 1.99E-05 | IGKV2D-28, IGHV1OR15-1, IGKC, IGHV4-59, IGKV3D-11, IGKV1D-33, IGHV3-7 | 1.50E-04 |
| Antigen activates B Cell Receptor (BCR) leading to generation of second messengers | 7 | 3.81E-05 | IGKV2D-28, IGHV1OR15-1, IGKC, IGHV4-59, IGKV3D-11, IGKV1D-33, IGHV3-7 | 2.64E-04 |
| FCERI mediated NF-kB activation | 7 | 3.26E-04 | IGKV2D-28, IGHV1OR15-1, IGKC, IGHV4-59, IGKV3D-11, IGKV1D-33, IGHV3-7 | 0.0020801 |
| Immunoregulatory interactions between a Lymphoid and a non-Lymphoid cell | 7 | 0.0016221 | IGKV2D-28, IGHV1OR15-1, IGKC, IGHV4-59, IGKV3D-11, IGKV1D-33, IGHV3-7 | 0.0096167 |
| Tryptophan catabolism | 3 | 0.0024277 | TDO2, KYNU, KMO | 0.0134335 |
| Collagen degradation | 4 | 0.0094117 | MMP12, MMP7, MMP8, MMP9 | 0.0488234 |
| Regulation of Complement cascade | 3 | 0.0154873 | CR1, C7, C2 | 0.0740877 |
| Degradation of the extracellular matrix | 4 | 0.0160672 | MMP12, MMP7, MMP8, MMP9 | 0.0740877 |
| Activation of Matrix Metalloproteinases | 3 | 0.0211666 | MMP7, MMP8, MMP9 | 0.0924647 |
| Activation of AMPA receptors | 2 | 0.0270535 | GRIA1, GRIA2 | 0.1122719 |
