# Supplementary material for: Exploration of Crucial Mediators for Carotid Atherosclerosis Pathogenesis Through Integration of Microbiome, Metabolome, and Transcriptome
Source: Front Physiol. 2021 May 24;12:645212. doi: 10.3389/fphys.2021.645212 (PMC8181762; doi:10.3389/fphys.2021.645212)
Supplement: Supplementary Table 10 — Details for correlation analysis. [file Table_10.DOCX]

**Table S7. Details for correlation analysis.**

| **Var1** | **Var2** | **cor** | ***p*-value** | **FDR** |
| --- | --- | --- | --- | --- |
| Ethanolamine | g__Gemella | 0.307793503 | 0.013353346 | 0.075151391 |
| Gly_Pro | g__Gemella | 0.306551373 | 0.013750815 | 0.07562948 |
| Propoxur | g__Gemella | 0.327954232 | 0.008156395 | 0.051943359 |
| Homocitrate | g__Gemella | -0.12379898 | 0.329744894 | 0.378190826 |
| Alpha_N_Phenylacetyl_L_glutamine | g__Gemella | -0.152240578 | 0.229776222 | 0.294842665 |
| Diethylcarbamazine | g__Gemella | -0.057743131 | 0.650391079 | 0.678425177 |
| Dimethylbenzimidazole | g__Gemella | -0.302793132 | 0.015016082 | 0.075634433 |
| Eicosapentaenoic.acid | g__Gemella | 0.359803725 | 0.003498298 | 0.044703529 |
| Decanoyl_L_carnitine | g__Gemella | 0.205620328 | 0.103099137 | 0.172068904 |
| X3_Methoxy_4_Hydroxyphenylglycol.Sulfate | g__Gemella | -0.138194951 | 0.276151585 | 0.330428096 |
| O_Desmethylnaproxen | g__Gemella | 0.250942157 | 0.045489568 | 0.113022306 |
| Salicylic.acid | g__Gemella | -0.040990298 | 0.747759914 | 0.766770759 |
| X3_Aminopropanesulphonic.Acid | g__Gemella | -0.230526632 | 0.066857019 | 0.133769971 |
| X6_Hydroxynicotinic.acid | g__Gemella | -0.210270354 | 0.095373166 | 0.169195479 |
| Formylanthranilic.acid | g__Gemella | -0.2357181 | 0.060779665 | 0.133204277 |
| Xanthopterin | g__Gemella | 0.264382644 | 0.034763969 | 0.101360006 |
| N1_Methyl_4_pyridone_3_carboxamide | g__Gemella | 0.201161399 | 0.110955272 | 0.179451178 |
| X3_Hydroxydodecanoic.acid | g__Gemella | 0.15208133 | 0.230269685 | 0.294842665 |
| Salicyluric.acid | g__Gemella | -0.051628029 | 0.685364139 | 0.711837432 |
| Phenylacetylglycine | g__Gemella | -0.342827945 | 0.005551687 | 0.046327869 |
| D_Biotin | g__Gemella | 0.265529225 | 0.033955882 | 0.101360006 |
| X5_10_methylene_THF | g__Gemella | 0.129531889 | 0.307663501 | 0.357954651 |
| Ethanolamine | g__Lactobacillus | -0.269393077 | 0.031347175 | 0.099147756 |
| Gly_Pro | g__Lactobacillus | -0.265504133 | 0.033973397 | 0.101360006 |
| Propoxur | g__Lactobacillus | -0.288606006 | 0.020733991 | 0.08064103 |
| Homocitrate | g__Lactobacillus | 0.438832566 | 0.000286382 | 0.015815606 |
| Alpha_N_Phenylacetyl_L_glutamine | g__Lactobacillus | 0.215025124 | 0.087948595 | 0.163540249 |
| Diethylcarbamazine | g__Lactobacillus | 0.201452452 | 0.110428803 | 0.179451178 |
| Dimethylbenzimidazole | g__Lactobacillus | 0.369269401 | 0.002674742 | 0.041287255 |
| Eicosapentaenoic.acid | g__Lactobacillus | -0.252781362 | 0.04387828 | 0.111774145 |
| Decanoyl_L_carnitine | g__Lactobacillus | -0.264628477 | 0.034589375 | 0.101360006 |
| X3_Methoxy_4_Hydroxyphenylglycol.Sulfate | g__Lactobacillus | 0.208019874 | 0.099053832 | 0.171006527 |
| O_Desmethylnaproxen | g__Lactobacillus | -0.175852383 | 0.164544008 | 0.231509593 |
| Salicylic.acid | g__Lactobacillus | 0.304470837 | 0.014439407 | 0.075634433 |
| X3_Aminopropanesulphonic.Acid | g__Lactobacillus | 0.14002774 | 0.269770529 | 0.32642234 |
| X6_Hydroxynicotinic.acid | g__Lactobacillus | 0.18821459 | 0.136385522 | 0.203736397 |
| Formylanthranilic.acid | g__Lactobacillus | 0.086561198 | 0.49642885 | 0.533936808 |
| Xanthopterin | g__Lactobacillus | -0.233516924 | 0.063299553 | 0.133204277 |
| N1_Methyl_4_pyridone_3_carboxamide | g__Lactobacillus | -0.318017755 | 0.010441429 | 0.063170644 |
| X3_Hydroxydodecanoic.acid | g__Lactobacillus | -0.207247236 | 0.100342673 | 0.171006527 |
| Salicyluric.acid | g__Lactobacillus | 0.253682773 | 0.04310591 | 0.110974791 |
| Phenylacetylglycine | g__Lactobacillus | 0.220253307 | 0.080317879 | 0.154261323 |
| D_Biotin | g__Lactobacillus | -0.230503637 | 0.066884985 | 0.133769971 |
| X5_10_methylene_THF | g__Lactobacillus | -0.071958342 | 0.572036944 | 0.604510657 |
| Ethanolamine | g__Christensenella | -0.354803369 | 0.00401842 | 0.044703529 |
| Gly_Pro | g__Christensenella | -0.331768847 | 0.007402914 | 0.04961242 |
| Propoxur | g__Christensenella | -0.336949622 | 0.006477435 | 0.047501192 |
| Homocitrate | g__Christensenella | 0.115451574 | 0.363639611 | 0.407411046 |
| Alpha_N_Phenylacetyl_L_glutamine | g__Christensenella | 0.240666915 | 0.055409859 | 0.131273305 |
| Diethylcarbamazine | g__Christensenella | 0.231261816 | 0.065967845 | 0.133769971 |
| Dimethylbenzimidazole | g__Christensenella | 0.294706382 | 0.018081424 | 0.078136017 |
| Eicosapentaenoic.acid | g__Christensenella | -0.234609394 | 0.062038574 | 0.133204277 |
| Decanoyl_L_carnitine | g__Christensenella | -0.124936377 | 0.325286272 | 0.376647262 |
| X3_Methoxy_4_Hydroxyphenylglycol.Sulfate | g__Christensenella | 0.249593789 | 0.04670149 | 0.114159198 |
| O_Desmethylnaproxen | g__Christensenella | -0.352770911 | 0.004248683 | 0.044703529 |
| Salicylic.acid | g__Christensenella | 0.186786857 | 0.139438616 | 0.207019294 |
| X3_Aminopropanesulphonic.Acid | g__Christensenella | 0.059260093 | 0.641826804 | 0.672389985 |
| X6_Hydroxynicotinic.acid | g__Christensenella | 0.254774564 | 0.04218546 | 0.109945001 |
| Formylanthranilic.acid | g__Christensenella | 0.233892056 | 0.06286425 | 0.133204277 |
| Xanthopterin | g__Christensenella | -0.190214139 | 0.132194616 | 0.199944357 |
| N1_Methyl_4_pyridone_3_carboxamide | g__Christensenella | -0.270237184 | 0.030800178 | 0.099147756 |
| X3_Hydroxydodecanoic.acid | g__Christensenella | -0.196112252 | 0.120399199 | 0.185212288 |
| Salicyluric.acid | g__Christensenella | 0.265375226 | 0.034063498 | 0.101360006 |
| Phenylacetylglycine | g__Christensenella | 0.251785655 | 0.04474466 | 0.112793831 |
| D_Biotin | g__Christensenella | -0.313755692 | 0.011580417 | 0.068352706 |
| X5_10_methylene_THF | g__Christensenella | -0.282033409 | 0.023955225 | 0.089187145 |
| Ethanolamine | g__Parvimonas | 0.334712981 | 0.006863732 | 0.048774617 |
| Gly_Pro | g__Parvimonas | 0.35542861 | 0.003949843 | 0.044703529 |
| Propoxur | g__Parvimonas | 0.333650641 | 0.00705418 | 0.048774617 |
| Homocitrate | g__Parvimonas | -0.207383944 | 0.100113686 | 0.171006527 |
| Alpha_N_Phenylacetyl_L_glutamine | g__Parvimonas | -0.201009904 | 0.111230069 | 0.179451178 |
| Diethylcarbamazine | g__Parvimonas | -0.1009223 | 0.427489736 | 0.474552826 |
| Dimethylbenzimidazole | g__Parvimonas | -0.288045902 | 0.020993326 | 0.08064103 |
| Eicosapentaenoic.acid | g__Parvimonas | 0.209584505 | 0.096483372 | 0.169195479 |
| Decanoyl_L_carnitine | g__Parvimonas | 0.300338693 | 0.015895346 | 0.075634433 |
| X3_Methoxy_4_Hydroxyphenylglycol.Sulfate | g__Parvimonas | -0.170960858 | 0.17679198 | 0.240357636 |
| O_Desmethylnaproxen | g__Parvimonas | 0.340100562 | 0.005965635 | 0.046685613 |
| Salicylic.acid | g__Parvimonas | -0.213075051 | 0.090936644 | 0.165463667 |
| X3_Aminopropanesulphonic.Acid | g__Parvimonas | -0.262549742 | 0.036089001 | 0.102375289 |
| X6_Hydroxynicotinic.acid | g__Parvimonas | -0.076184954 | 0.549614499 | 0.585932638 |
| Formylanthranilic.acid | g__Parvimonas | -0.259590366 | 0.038316884 | 0.10537143 |
| Xanthopterin | g__Parvimonas | 0.291991736 | 0.019223665 | 0.078136017 |
| N1_Methyl_4_pyridone_3_carboxamide | g__Parvimonas | 0.207839232 | 0.099354001 | 0.171006527 |
| X3_Hydroxydodecanoic.acid | g__Parvimonas | 0.197822884 | 0.117133247 | 0.183719282 |
| Salicyluric.acid | g__Parvimonas | -0.269758478 | 0.031109398 | 0.099147756 |
| Phenylacetylglycine | g__Parvimonas | -0.211785067 | 0.092956625 | 0.16787689 |
| D_Biotin | g__Parvimonas | 0.308078599 | 0.013263529 | 0.075151391 |
| X5_10_methylene_THF | g__Parvimonas | 0.156922794 | 0.215594272 | 0.280715063 |
| Ethanolamine | g__Anaerostipes | 0.159570033 | 0.207853933 | 0.273373107 |
| Gly_Pro | g__Anaerostipes | 0.152599306 | 0.228667315 | 0.294842665 |
| Propoxur | g__Anaerostipes | 0.138955942 | 0.273490026 | 0.329276549 |
| Homocitrate | g__Anaerostipes | -0.296668643 | 0.017292324 | 0.078136017 |
| Alpha_N_Phenylacetyl_L_glutamine | g__Anaerostipes | -0.164591708 | 0.193715443 | 0.259000758 |
| Diethylcarbamazine | g__Anaerostipes | -0.223040338 | 0.076470053 | 0.149239943 |
| Dimethylbenzimidazole | g__Anaerostipes | -0.261815007 | 0.036631815 | 0.102375289 |
| Eicosapentaenoic.acid | g__Anaerostipes | 0.132076935 | 0.298174277 | 0.348590218 |
| Decanoyl_L_carnitine | g__Anaerostipes | 0.133498596 | 0.292957534 | 0.344153996 |
| X3_Methoxy_4_Hydroxyphenylglycol.Sulfate | g__Anaerostipes | -0.151406945 | 0.23236756 | 0.295962893 |
| O_Desmethylnaproxen | g__Anaerostipes | 0.02953387 | 0.81679841 | 0.82705111 |
| Salicylic.acid | g__Anaerostipes | -0.174612128 | 0.167588805 | 0.234270455 |
| X3_Aminopropanesulphonic.Acid | g__Anaerostipes | -0.034486755 | 0.786747267 | 0.803345311 |
| X6_Hydroxynicotinic.acid | g__Anaerostipes | -0.049437131 | 0.698060823 | 0.72192615 |
| Formylanthranilic.acid | g__Anaerostipes | -0.181422345 | 0.151369841 | 0.220671696 |
| Xanthopterin | g__Anaerostipes | 0.258260854 | 0.039354269 | 0.107008237 |
| N1_Methyl_4_pyridone_3_carboxamide | g__Anaerostipes | 0.188737023 | 0.13528101 | 0.203341643 |
| X3_Hydroxydodecanoic.acid | g__Anaerostipes | 0.11698898 | 0.357242395 | 0.405881031 |
| Salicyluric.acid | g__Anaerostipes | -0.099814392 | 0.432606228 | 0.478039759 |
| Phenylacetylglycine | g__Anaerostipes | -0.172754796 | 0.172225413 | 0.238163142 |
| D_Biotin | g__Anaerostipes | 0.222077277 | 0.077782736 | 0.150587378 |
| X5_10_methylene_THF | g__Anaerostipes | 0.179886034 | 0.154922595 | 0.221841823 |
| Ethanolamine | g__Clostridium.XlVa | 0.226510989 | 0.071885016 | 0.141432308 |
| Gly_Pro | g__Clostridium.XlVa | 0.19775641 | 0.117258878 | 0.183719282 |
| Propoxur | g__Clostridium.XlVa | 0.201877289 | 0.109663795 | 0.179451178 |
| Homocitrate | g__Clostridium.XlVa | -0.470970696 | 8.58E-05 | 0.006918801 |
| Alpha_N_Phenylacetyl_L_glutamine | g__Clostridium.XlVa | -0.330815018 | 0.00758537 | 0.04961242 |
| Diethylcarbamazine | g__Clostridium.XlVa | -0.404624542 | 0.000912366 | 0.022321804 |
| Dimethylbenzimidazole | g__Clostridium.XlVa | -0.238827839 | 0.057358484 | 0.131273305 |
| Eicosapentaenoic.acid | g__Clostridium.XlVa | 0.239148352 | 0.057014936 | 0.131273305 |
| Decanoyl_L_carnitine | g__Clostridium.XlVa | 0.146749084 | 0.247218596 | 0.308386083 |
| X3_Methoxy_4_Hydroxyphenylglycol.Sulfate | g__Clostridium.XlVa | -0.349358974 | 0.004661552 | 0.046327869 |
| O_Desmethylnaproxen | g__Clostridium.XlVa | 0.090613553 | 0.476410303 | 0.51933015 |
| Salicylic.acid | g__Clostridium.XlVa | -0.232142857 | 0.064914814 | 0.133769971 |
| X3_Aminopropanesulphonic.Acid | g__Clostridium.XlVa | -0.060714286 | 0.633660293 | 0.66672083 |
| X6_Hydroxynicotinic.acid | g__Clostridium.XlVa | -0.122802198 | 0.333683895 | 0.380903314 |
| Formylanthranilic.acid | g__Clostridium.XlVa | -0.174267399 | 0.168442393 | 0.234270455 |
| Xanthopterin | g__Clostridium.XlVa | 0.140796703 | 0.267122923 | 0.324842951 |
| N1_Methyl_4_pyridone_3_carboxamide | g__Clostridium.XlVa | 0.280082418 | 0.024989267 | 0.091627312 |
| X3_Hydroxydodecanoic.acid | g__Clostridium.XlVa | 0.161401099 | 0.202616384 | 0.267940792 |
| Salicyluric.acid | g__Clostridium.XlVa | -0.346840659 | 0.004988625 | 0.046327869 |
| Phenylacetylglycine | g__Clostridium.XlVa | -0.156868132 | 0.215756205 | 0.280715063 |
| D_Biotin | g__Clostridium.XlVa | 0.180311355 | 0.153932904 | 0.221841823 |
| X5_10_methylene_THF | g__Clostridium.XlVa | 0.166300366 | 0.189065584 | 0.254188174 |
| Ethanolamine | g__Clostridium.XlVb | 0.368561457 | 0.002729736 | 0.041287255 |
| Gly_Pro | g__Clostridium.XlVb | 0.354456471 | 0.004056921 | 0.044703529 |
| Propoxur | g__Clostridium.XlVb | 0.345961422 | 0.005107494 | 0.046327869 |
| Homocitrate | g__Clostridium.XlVb | -0.371492363 | 0.002508422 | 0.041287255 |
| Alpha_N_Phenylacetyl_L_glutamine | g__Clostridium.XlVb | -0.238067437 | 0.058180257 | 0.131273305 |
| Diethylcarbamazine | g__Clostridium.XlVb | -0.321873037 | 0.009496205 | 0.058925172 |
| Dimethylbenzimidazole | g__Clostridium.XlVb | -0.272207915 | 0.02955416 | 0.099147756 |
| Eicosapentaenoic.acid | g__Clostridium.XlVb | 0.1975385 | 0.117671441 | 0.183719282 |
| Decanoyl_L_carnitine | g__Clostridium.XlVb | 0.23364818 | 0.063146968 | 0.133204277 |
| X3_Methoxy_4_Hydroxyphenylglycol.Sulfate | g__Clostridium.XlVb | -0.264811957 | 0.034459542 | 0.101360006 |
| O_Desmethylnaproxen | g__Clostridium.XlVb | 0.238708573 | 0.05748675 | 0.131273305 |
| Salicylic.acid | g__Clostridium.XlVb | -0.338794441 | 0.006173304 | 0.046685613 |
| X3_Aminopropanesulphonic.Acid | g__Clostridium.XlVb | -0.180067551 | 0.154499644 | 0.221841823 |
| X6_Hydroxynicotinic.acid | g__Clostridium.XlVb | -0.229091537 | 0.068620429 | 0.136115934 |
| Formylanthranilic.acid | g__Clostridium.XlVb | -0.171824377 | 0.17458298 | 0.238860489 |
| Xanthopterin | g__Clostridium.XlVb | 0.26158338 | 0.03680434 | 0.102375289 |
| N1_Methyl_4_pyridone_3_carboxamide | g__Clostridium.XlVb | 0.209651386 | 0.096374669 | 0.169195479 |
| X3_Hydroxydodecanoic.acid | g__Clostridium.XlVb | 0.17049631 | 0.177988729 | 0.240632806 |
| Salicyluric.acid | g__Clostridium.XlVb | -0.291648067 | 0.019372566 | 0.078136017 |
| Phenylacetylglycine | g__Clostridium.XlVb | -0.140958271 | 0.266568855 | 0.324842951 |
| D_Biotin | g__Clostridium.XlVb | 0.294166814 | 0.018303723 | 0.078136017 |
| X5_10_methylene_THF | g__Clostridium.XlVb | 0.387497859 | 0.001559012 | 0.031440073 |
| Ethanolamine | g__Romboutsia | 0.403598676 | 0.000942879 | 0.022321804 |
| Gly_Pro | g__Romboutsia | 0.421299364 | 0.000526611 | 0.01820571 |
| Propoxur | g__Romboutsia | 0.425253284 | 0.000460334 | 0.01820571 |
| Homocitrate | g__Romboutsia | -0.271326262 | 0.030106267 | 0.099147756 |
| Alpha_N_Phenylacetyl_L_glutamine | g__Romboutsia | -0.143490513 | 0.257985737 | 0.318243632 |
| Diethylcarbamazine | g__Romboutsia | -0.076779608 | 0.546494084 | 0.58518393 |
| Dimethylbenzimidazole | g__Romboutsia | -0.401299885 | 0.001014627 | 0.022321804 |
| Eicosapentaenoic.acid | g__Romboutsia | 0.237786906 | 0.05848583 | 0.131273305 |
| Decanoyl_L_carnitine | g__Romboutsia | 0.491711322 | 3.69E-05 | 0.004461852 |
| X3_Methoxy_4_Hydroxyphenylglycol.Sulfate | g__Romboutsia | -0.087974719 | 0.489396795 | 0.528723323 |
| O_Desmethylnaproxen | g__Romboutsia | 0.302957621 | 0.014958681 | 0.075634433 |
| Salicylic.acid | g__Romboutsia | -0.162685415 | 0.198999167 | 0.264603288 |
| X3_Aminopropanesulphonic.Acid | g__Romboutsia | -0.042458663 | 0.739039947 | 0.761053903 |
| X6_Hydroxynicotinic.acid | g__Romboutsia | -0.01328701 | 0.917006165 | 0.917006165 |
| Formylanthranilic.acid | g__Romboutsia | -0.143168682 | 0.259066097 | 0.318243632 |
| Xanthopterin | g__Romboutsia | 0.34514043 | 0.005220737 | 0.046327869 |
| N1_Methyl_4_pyridone_3_carboxamide | g__Romboutsia | 0.414908726 | 0.000652254 | 0.019730697 |
| X3_Hydroxydodecanoic.acid | g__Romboutsia | 0.506354619 | 1.96E-05 | 0.004461852 |
| Salicyluric.acid | g__Romboutsia | -0.08818161 | 0.48837195 | 0.528723323 |
| Phenylacetylglycine | g__Romboutsia | -0.179121768 | 0.156712817 | 0.223085304 |
| D_Biotin | g__Romboutsia | 0.380426866 | 0.001929169 | 0.035912215 |
| X5_10_methylene_THF | g__Romboutsia | 0.255763449 | 0.041365824 | 0.109945001 |
| Ethanolamine | g__Clostridium.XVIII | 0.301584728 | 0.015443607 | 0.075634433 |
| Gly_Pro | g__Clostridium.XVIII | 0.291938078 | 0.019246849 | 0.078136017 |
| Propoxur | g__Clostridium.XVIII | 0.293438156 | 0.018607627 | 0.078136017 |
| Homocitrate | g__Clostridium.XVIII | -0.353695098 | 0.00414257 | 0.044703529 |
| Alpha_N_Phenylacetyl_L_glutamine | g__Clostridium.XVIII | -0.231288808 | 0.065935382 | 0.133769971 |
| Diethylcarbamazine | g__Clostridium.XVIII | -0.195840833 | 0.120923725 | 0.185212288 |
| Dimethylbenzimidazole | g__Clostridium.XVIII | -0.28886869 | 0.020613306 | 0.08064103 |
| Eicosapentaenoic.acid | g__Clostridium.XVIII | 0.237427585 | 0.058879122 | 0.131273305 |
| Decanoyl_L_carnitine | g__Clostridium.XVIII | 0.246728063 | 0.049365344 | 0.119464132 |
| X3_Methoxy_4_Hydroxyphenylglycol.Sulfate | g__Clostridium.XVIII | -0.198240956 | 0.116345475 | 0.183719282 |
| O_Desmethylnaproxen | g__Clostridium.XVIII | 0.148230694 | 0.242425993 | 0.305557761 |
| Salicylic.acid | g__Clostridium.XVIII | -0.210472354 | 0.095048093 | 0.169195479 |
| X3_Aminopropanesulphonic.Acid | g__Clostridium.XVIII | -0.095935699 | 0.450788631 | 0.495867494 |
| X6_Hydroxynicotinic.acid | g__Clostridium.XVIII | -0.133945344 | 0.291330625 | 0.34391225 |
| Formylanthranilic.acid | g__Clostridium.XVIII | -0.28508388 | 0.022411083 | 0.084741906 |
| Xanthopterin | g__Clostridium.XVIII | 0.435114666 | 0.000326769 | 0.015815606 |
| N1_Methyl_4_pyridone_3_carboxamide | g__Clostridium.XVIII | 0.339525139 | 0.006056356 | 0.046685613 |
| X3_Hydroxydodecanoic.acid | g__Clostridium.XVIII | 0.218549692 | 0.082744466 | 0.156438756 |
| Salicyluric.acid | g__Clostridium.XVIII | -0.176847549 | 0.162130443 | 0.229447761 |
| Phenylacetylglycine | g__Clostridium.XVIII | -0.277652729 | 0.0263295 | 0.093702045 |
| D_Biotin | g__Clostridium.XVIII | 0.362072452 | 0.003282695 | 0.044703529 |
| X5_10_methylene_THF | g__Clostridium.XVIII | 0.250628263 | 0.045769364 | 0.113022306 |
| Ethanolamine | g__Acidaminococcus | -0.293687152 | 0.018503297 | 0.078136017 |
| Gly_Pro | g__Acidaminococcus | -0.273935381 | 0.028497 | 0.099084144 |
| Propoxur | g__Acidaminococcus | -0.273664396 | 0.028660703 | 0.099084144 |
| Homocitrate | g__Acidaminococcus | 0.343819313 | 0.005407612 | 0.046327869 |
| Alpha_N_Phenylacetyl_L_glutamine | g__Acidaminococcus | 0.214680069 | 0.088471614 | 0.163540249 |
| Diethylcarbamazine | g__Acidaminococcus | 0.214228428 | 0.089159882 | 0.163540249 |
| Dimethylbenzimidazole | g__Acidaminococcus | 0.305038398 | 0.014248682 | 0.075634433 |
| Eicosapentaenoic.acid | g__Acidaminococcus | -0.269449079 | 0.031310634 | 0.099147756 |
| Decanoyl_L_carnitine | g__Acidaminococcus | -0.184058802 | 0.145414642 | 0.214575264 |
| X3_Methoxy_4_Hydroxyphenylglycol.Sulfate | g__Acidaminococcus | 0.193242171 | 0.12603438 | 0.191825912 |
| O_Desmethylnaproxen | g__Acidaminococcus | -0.219738449 | 0.081045208 | 0.154432601 |
| Salicylic.acid | g__Acidaminococcus | 0.269087766 | 0.031547013 | 0.099147756 |
| X3_Aminopropanesulphonic.Acid | g__Acidaminococcus | 0.027188794 | 0.831122 | 0.838048017 |
| X6_Hydroxynicotinic.acid | g__Acidaminococcus | 0.233799542 | 0.062971379 | 0.133204277 |
| Formylanthranilic.acid | g__Acidaminococcus | 0.149493204 | 0.238392738 | 0.302047344 |
| Xanthopterin | g__Acidaminococcus | -0.206038669 | 0.102384797 | 0.172063339 |
| N1_Methyl_4_pyridone_3_carboxamide | g__Acidaminococcus | -0.292302119 | 0.019090026 | 0.078136017 |
| X3_Hydroxydodecanoic.acid | g__Acidaminococcus | -0.263788511 | 0.035188964 | 0.101377728 |
| Salicyluric.acid | g__Acidaminococcus | 0.25469547 | 0.042251591 | 0.109945001 |
| Phenylacetylglycine | g__Acidaminococcus | 0.199203834 | 0.114546578 | 0.18357796 |
| D_Biotin | g__Acidaminococcus | -0.237201905 | 0.059127232 | 0.131273305 |
| X5_10_methylene_THF | g__Acidaminococcus | -0.206309654 | 0.10192413 | 0.172063339 |
| Ethanolamine | g__Fusobacterium | 0.21419972 | 0.089203772 | 0.163540249 |
| Gly_Pro | g__Fusobacterium | 0.196052063 | 0.120515366 | 0.185212288 |
| Propoxur | g__Fusobacterium | 0.203561438 | 0.106671284 | 0.176811307 |
| Homocitrate | g__Fusobacterium | -0.257091813 | 0.04028556 | 0.108323394 |
| Alpha_N_Phenylacetyl_L_glutamine | g__Fusobacterium | -0.171776964 | 0.174703746 | 0.238860489 |
| Diethylcarbamazine | g__Fusobacterium | -0.112979597 | 0.374071082 | 0.417166829 |
| Dimethylbenzimidazole | g__Fusobacterium | -0.278211931 | 0.026015791 | 0.093702045 |
| Eicosapentaenoic.acid | g__Fusobacterium | 0.239361343 | 0.056787562 | 0.131273305 |
| Decanoyl_L_carnitine | g__Fusobacterium | 0.124452254 | 0.327179348 | 0.377035248 |
| X3_Methoxy_4_Hydroxyphenylglycol.Sulfate | g__Fusobacterium | -0.137828329 | 0.277439974 | 0.330428096 |
| O_Desmethylnaproxen | g__Fusobacterium | 0.116160652 | 0.360680468 | 0.405975224 |
| Salicylic.acid | g__Fusobacterium | -0.094310455 | 0.458531025 | 0.502101846 |
| X3_Aminopropanesulphonic.Acid | g__Fusobacterium | -0.030924025 | 0.808335202 | 0.821920668 |
| X6_Hydroxynicotinic.acid | g__Fusobacterium | -0.074859087 | 0.556602612 | 0.590779966 |
| Formylanthranilic.acid | g__Fusobacterium | -0.14630245 | 0.248675945 | 0.308613224 |
| Xanthopterin | g__Fusobacterium | 0.230626479 | 0.066735696 | 0.133769971 |
| N1_Methyl_4_pyridone_3_carboxamide | g__Fusobacterium | 0.300218573 | 0.015939488 | 0.075634433 |
| X3_Hydroxydodecanoic.acid | g__Fusobacterium | 0.137515438 | 0.278542693 | 0.330428096 |
| Salicyluric.acid | g__Fusobacterium | -0.147371493 | 0.245197447 | 0.307449649 |
| Phenylacetylglycine | g__Fusobacterium | -0.181528722 | 0.151126103 | 0.220671696 |
| D_Biotin | g__Fusobacterium | 0.11639532 | 0.359704401 | 0.405975224 |
| X5_10_methylene_THF | g__Fusobacterium | 0.023545021 | 0.853485293 | 0.857026725 |
| CASQ2 | Ethanolamine | 0.567216117 | 1.02E-06 | 4.50591E-05 |
| CD36 | Ethanolamine | -0.477014652 | 6.74E-05 | 0.000485901 |
| CNTN1 | Ethanolamine | 0.527884615 | 7.37E-06 | 0.000181398 |
| CNTN4 | Ethanolamine | 0.531393445 | 6.24E-06 | 0.000181398 |
| FABP4 | Ethanolamine | -0.494871795 | 3.23E-05 | 0.000364041 |
| IBSP | Ethanolamine | -0.59514652 | 2.14E-07 | 3.61491E-05 |
| IGHV3_43 | Ethanolamine | -0.489789377 | 4.00E-05 | 0.000399093 |
| IGHV3_52 | Ethanolamine | -0.501923077 | 2.38E-05 | 0.000349689 |
| IGHV4_59 | Ethanolamine | -0.459340659 | 0.000134535 | 0.000672677 |
| IGJ | Ethanolamine | -0.425228938 | 0.000460718 | 0.001793946 |
| IGKC | Ethanolamine | -0.484935897 | 4.89E-05 | 0.00041591 |
| IGKV1D_27 | Ethanolamine | -0.476648352 | 6.84E-05 | 0.000485901 |
| IGKV1D_33 | Ethanolamine | -0.497527473 | 2.88E-05 | 0.00035796 |
| IGKV1OR2_3 | Ethanolamine | -0.478205128 | 6.43E-05 | 0.000485901 |
| IGKV2D_26 | Ethanolamine | -0.483699634 | 5.14E-05 | 0.000426897 |
| IGKV3D_11 | Ethanolamine | -0.469230769 | 9.18E-05 | 0.000553558 |
| IGKV3D_20 | Ethanolamine | -0.482554945 | 5.39E-05 | 0.000439084 |
| MMP7 | Ethanolamine | -0.443360806 | 0.000243372 | 0.001049839 |
| MMP9 | Ethanolamine | -0.453205128 | 0.000169504 | 0.000810673 |
| TPH1 | Ethanolamine | 0.46717033 | 9.95E-05 | 0.000573888 |
| CASQ2 | Gly_Pro | 0.568406593 | 9.61E-07 | 4.50591E-05 |
| CD36 | Gly_Pro | -0.475457875 | 7.18E-05 | 0.000485901 |
| CNTN1 | Gly_Pro | 0.528296703 | 7.23E-06 | 0.000181398 |
| CNTN4 | Gly_Pro | 0.532400783 | 5.95E-06 | 0.000181398 |
| FABP4 | Gly_Pro | -0.496474359 | 3.01E-05 | 0.00035796 |
| IBSP | Gly_Pro | -0.591758242 | 2.61E-07 | 3.61491E-05 |
| IGHV3_43 | Gly_Pro | -0.476053114 | 7.01E-05 | 0.000485901 |
| IGHV3_52 | Gly_Pro | -0.485760073 | 4.72E-05 | 0.000415749 |
| IGHV4_59 | Gly_Pro | -0.448489011 | 0.000201852 | 0.000923352 |
| IGJ | Gly_Pro | -0.407738095 | 0.000825143 | 0.002913142 |
| IGKC | Gly_Pro | -0.470512821 | 8.73E-05 | 0.000533688 |
| IGKV1D_27 | Gly_Pro | -0.465888278 | 0.000104625 | 0.000590193 |
| IGKV1D_33 | Gly_Pro | -0.481959707 | 5.52E-05 | 0.0004417 |
| IGKV1OR2_3 | Gly_Pro | -0.460989011 | 0.000126343 | 0.000645718 |
| IGKV2D_26 | Gly_Pro | -0.479349817 | 6.14E-05 | 0.00047385 |
| IGKV3D_11 | Gly_Pro | -0.460714286 | 0.000127676 | 0.000645718 |
| IGKV3D_20 | Gly_Pro | -0.471932234 | 8.26E-05 | 0.000526575 |
| MMP7 | Gly_Pro | -0.441346154 | 0.000261719 | 0.001096728 |
| MMP9 | Gly_Pro | -0.449404762 | 0.00019516 | 0.000913513 |
| TPH1 | Gly_Pro | 0.475732601 | 7.10E-05 | 0.000485901 |
| CASQ2 | Propoxur | 0.561492674 | 1.39E-06 | 5.54678E-05 |
| CD36 | Propoxur | -0.464423077 | 0.00011073 | 0.000602619 |
| CNTN1 | Propoxur | 0.527747253 | 7.42E-06 | 0.000181398 |
| CNTN4 | Propoxur | 0.531004247 | 6.36E-06 | 0.000181398 |
| FABP4 | Propoxur | -0.491575092 | 3.71E-05 | 0.000388525 |
| IBSP | Propoxur | -0.587728938 | 3.29E-07 | 3.61491E-05 |
| IGHV3_43 | Propoxur | -0.481181319 | 5.70E-05 | 0.000447784 |
| IGHV3_52 | Propoxur | -0.490155678 | 3.94E-05 | 0.000399093 |
| IGHV4_59 | Propoxur | -0.452701465 | 0.000172717 | 0.000817154 |
| IGJ | Propoxur | -0.406318681 | 0.000863925 | 0.002993126 |
| IGKC | Propoxur | -0.472664835 | 8.02E-05 | 0.00052378 |
| IGKV1D_27 | Propoxur | -0.466941392 | 0.00010043 | 0.000573888 |
| IGKV1D_33 | Propoxur | -0.484798535 | 4.92E-05 | 0.00041591 |
| IGKV1OR2_3 | Propoxur | -0.464056777 | 0.000112306 | 0.000602619 |
| IGKV2D_26 | Propoxur | -0.485943223 | 4.69E-05 | 0.000415749 |
| IGKV3D_11 | Propoxur | -0.462545788 | 0.00011903 | 0.000631004 |
| IGKV3D_20 | Propoxur | -0.475457875 | 7.18E-05 | 0.000485901 |
| MMP7 | Propoxur | -0.42481685 | 0.000467258 | 0.00180345 |
| MMP9 | Propoxur | -0.4375 | 0.000300301 | 0.001234881 |
| TPH1 | Propoxur | 0.464285714 | 0.000111319 | 0.000602619 |
| CASQ2 | Homocitrate | -0.471108059 | 8.53E-05 | 0.000528664 |
| CD36 | Homocitrate | 0.364697802 | 0.003048002 | 0.007450671 |
| CNTN1 | Homocitrate | -0.412225275 | 0.000712681 | 0.002570324 |
| CNTN4 | Homocitrate | -0.444235854 | 0.000235775 | 0.001027141 |
| FABP4 | Homocitrate | 0.314606227 | 0.0113449 | 0.019022148 |
| IBSP | Homocitrate | 0.426465201 | 0.000441596 | 0.00175047 |
| IGHV3_43 | Homocitrate | 0.339606227 | 0.006043499 | 0.012241876 |
| IGHV3_52 | Homocitrate | 0.341071429 | 0.005815273 | 0.011845926 |
| IGHV4_59 | Homocitrate | 0.361401099 | 0.003345231 | 0.007999464 |
| IGJ | Homocitrate | 0.331959707 | 0.00736687 | 0.013676889 |
| IGKC | Homocitrate | 0.344871795 | 0.005258268 | 0.011030776 |
| IGKV1D_27 | Homocitrate | 0.303159341 | 0.014888545 | 0.023735362 |
| IGKV1D_33 | Homocitrate | 0.341758242 | 0.005710926 | 0.011742091 |
| IGKV1OR2_3 | Homocitrate | 0.337362637 | 0.006408228 | 0.012644037 |
| IGKV2D_26 | Homocitrate | 0.323489011 | 0.009122707 | 0.016317038 |
| IGKV3D_11 | Homocitrate | 0.331043956 | 0.007541222 | 0.013883421 |
| IGKV3D_20 | Homocitrate | 0.337087912 | 0.00645419 | 0.012677873 |
| MMP7 | Homocitrate | 0.386446886 | 0.001609644 | 0.004575668 |
| MMP9 | Homocitrate | 0.454624542 | 0.000160744 | 0.000777225 |
| TPH1 | Homocitrate | -0.456868132 | 0.000147741 | 0.000730407 |
| CASQ2 | Alpha_N_Phenylacetyl_L_glutamine | -0.320375458 | 0.009854144 | 0.017205649 |
| CD36 | Alpha_N_Phenylacetyl_L_glutamine | 0.241804029 | 0.054232176 | 0.066468405 |
| CNTN1 | Alpha_N_Phenylacetyl_L_glutamine | -0.196932234 | 0.11882511 | 0.132027899 |
| CNTN4 | Alpha_N_Phenylacetyl_L_glutamine | -0.232214197 | 0.064830144 | 0.077095306 |
| FABP4 | Alpha_N_Phenylacetyl_L_glutamine | 0.275503663 | 0.027564952 | 0.039124448 |
| IBSP | Alpha_N_Phenylacetyl_L_glutamine | 0.338232601 | 0.006264571 | 0.012472449 |
| IGHV3_43 | Alpha_N_Phenylacetyl_L_glutamine | 0.276419414 | 0.027032669 | 0.038493121 |
| IGHV3_52 | Alpha_N_Phenylacetyl_L_glutamine | 0.27202381 | 0.029668743 | 0.041051091 |
| IGHV4_59 | Alpha_N_Phenylacetyl_L_glutamine | 0.239194139 | 0.056965994 | 0.068671335 |
| IGJ | Alpha_N_Phenylacetyl_L_glutamine | 0.312362637 | 0.011975305 | 0.019734586 |
| IGKC | Alpha_N_Phenylacetyl_L_glutamine | 0.278891941 | 0.025638561 | 0.037108444 |
| IGKV1D_27 | Alpha_N_Phenylacetyl_L_glutamine | 0.227289377 | 0.070887484 | 0.082953439 |
| IGKV1D_33 | Alpha_N_Phenylacetyl_L_glutamine | 0.298443223 | 0.016604253 | 0.026374987 |
| IGKV1OR2_3 | Alpha_N_Phenylacetyl_L_glutamine | 0.282554945 | 0.023685019 | 0.034507975 |
| IGKV2D_26 | Alpha_N_Phenylacetyl_L_glutamine | 0.235760073 | 0.060732414 | 0.072812703 |
| IGKV3D_11 | Alpha_N_Phenylacetyl_L_glutamine | 0.264468864 | 0.03470265 | 0.04698205 |
| IGKV3D_20 | Alpha_N_Phenylacetyl_L_glutamine | 0.269871795 | 0.031035967 | 0.042541513 |
| MMP7 | Alpha_N_Phenylacetyl_L_glutamine | 0.225732601 | 0.072893733 | 0.08462597 |
| MMP9 | Alpha_N_Phenylacetyl_L_glutamine | 0.24478022 | 0.051245998 | 0.06333775 |
| TPH1 | Alpha_N_Phenylacetyl_L_glutamine | -0.288049451 | 0.020991674 | 0.031523333 |
| CASQ2 | Diethylcarbamazine | -0.366071429 | 0.002931256 | 0.007245802 |
| CD36 | Diethylcarbamazine | 0.295100733 | 0.017920417 | 0.027598535 |
| CNTN1 | Diethylcarbamazine | -0.245833333 | 0.050222074 | 0.062599753 |
| CNTN4 | Diethylcarbamazine | -0.297439302 | 0.016990571 | 0.0266344 |
| FABP4 | Diethylcarbamazine | 0.323946886 | 0.009019233 | 0.016197806 |
| IBSP | Diethylcarbamazine | 0.377564103 | 0.002100166 | 0.005669159 |
| IGHV3_43 | Diethylcarbamazine | 0.254258242 | 0.042618711 | 0.054991885 |
| IGHV3_52 | Diethylcarbamazine | 0.252747253 | 0.043907729 | 0.056426308 |
| IGHV4_59 | Diethylcarbamazine | 0.228800366 | 0.068982723 | 0.081156145 |
| IGJ | Diethylcarbamazine | 0.286858974 | 0.021552012 | 0.032036774 |
| IGKC | Diethylcarbamazine | 0.262362637 | 0.036226593 | 0.048539214 |
| IGKV1D_27 | Diethylcarbamazine | 0.193452381 | 0.125614968 | 0.138870819 |
| IGKV1D_33 | Diethylcarbamazine | 0.279532967 | 0.025287194 | 0.036720678 |
| IGKV1OR2_3 | Diethylcarbamazine | 0.260164835 | 0.03787571 | 0.049896145 |
| IGKV2D_26 | Diethylcarbamazine | 0.231135531 | 0.066119902 | 0.078206335 |
| IGKV3D_11 | Diethylcarbamazine | 0.257051282 | 0.040318172 | 0.052797606 |
| IGKV3D_20 | Diethylcarbamazine | 0.254258242 | 0.042618711 | 0.054991885 |
| MMP7 | Diethylcarbamazine | 0.265750916 | 0.03380146 | 0.046088714 |
| MMP9 | Diethylcarbamazine | 0.296199634 | 0.0174782 | 0.027174587 |
| TPH1 | Diethylcarbamazine | -0.333516484 | 0.007078557 | 0.013253468 |
| CASQ2 | Dimethylbenzimidazole | -0.333562271 | 0.007070229 | 0.013253468 |
| CD36 | Dimethylbenzimidazole | 0.276648352 | 0.026900959 | 0.038429941 |
| CNTN1 | Dimethylbenzimidazole | -0.412637363 | 0.000703083 | 0.002556666 |
| CNTN4 | Dimethylbenzimidazole | -0.346821736 | 0.004991157 | 0.010694634 |
| FABP4 | Dimethylbenzimidazole | 0.277289377 | 0.026535043 | 0.038154964 |
| IBSP | Dimethylbenzimidazole | 0.394688645 | 0.00124927 | 0.003926278 |
| IGHV3_43 | Dimethylbenzimidazole | 0.389972527 | 0.001445396 | 0.004326355 |
| IGHV3_52 | Dimethylbenzimidazole | 0.391437729 | 0.001381689 | 0.004192712 |
| IGHV4_59 | Dimethylbenzimidazole | 0.371932234 | 0.002476627 | 0.006448023 |
| IGJ | Dimethylbenzimidazole | 0.371062271 | 0.002539862 | 0.006459765 |
| IGKC | Dimethylbenzimidazole | 0.371291209 | 0.002523083 | 0.006454399 |
| IGKV1D_27 | Dimethylbenzimidazole | 0.347435897 | 0.004909542 | 0.010659888 |
| IGKV1D_33 | Dimethylbenzimidazole | 0.373763736 | 0.002348073 | 0.006223808 |
| IGKV1OR2_3 | Dimethylbenzimidazole | 0.373305861 | 0.00237964 | 0.006232391 |
| IGKV2D_26 | Dimethylbenzimidazole | 0.374267399 | 0.002313782 | 0.006170085 |
| IGKV3D_11 | Dimethylbenzimidazole | 0.344871795 | 0.005258268 | 0.011030776 |
| IGKV3D_20 | Dimethylbenzimidazole | 0.339239927 | 0.006101771 | 0.012259265 |
| MMP7 | Dimethylbenzimidazole | 0.283470696 | 0.023216812 | 0.034051325 |
| MMP9 | Dimethylbenzimidazole | 0.261492674 | 0.036872084 | 0.049014251 |
| TPH1 | Dimethylbenzimidazole | -0.314880952 | 0.011269719 | 0.019022148 |
| CASQ2 | Eicosapentaenoic.acid | 0.288415751 | 0.020821774 | 0.031375276 |
| CD36 | Eicosapentaenoic.acid | -0.22293956 | 0.076606585 | 0.088237951 |
| CNTN1 | Eicosapentaenoic.acid | 0.334981685 | 0.00681628 | 0.013070716 |
| CNTN4 | Eicosapentaenoic.acid | 0.347371192 | 0.004918085 | 0.010659888 |
| FABP4 | Eicosapentaenoic.acid | -0.233150183 | 0.063727466 | 0.07598939 |
| IBSP | Eicosapentaenoic.acid | -0.260760073 | 0.037423041 | 0.049447862 |
| IGHV3_43 | Eicosapentaenoic.acid | -0.402655678 | 0.000971737 | 0.003233616 |
| IGHV3_52 | Eicosapentaenoic.acid | -0.407234432 | 0.000838721 | 0.002928865 |
| IGHV4_59 | Eicosapentaenoic.acid | -0.423214286 | 0.0004935 | 0.001871897 |
| IGJ | Eicosapentaenoic.acid | -0.405769231 | 0.000879374 | 0.003003846 |
| IGKC | Eicosapentaenoic.acid | -0.393452381 | 0.001298217 | 0.004022643 |
| IGKV1D_27 | Eicosapentaenoic.acid | -0.440659341 | 0.000268258 | 0.001113522 |
| IGKV1D_33 | Eicosapentaenoic.acid | -0.40952381 | 0.000778594 | 0.002785214 |
| IGKV1OR2_3 | Eicosapentaenoic.acid | -0.468452381 | 9.47E-05 | 0.000562974 |
| IGKV2D_26 | Eicosapentaenoic.acid | -0.354624542 | 0.004038227 | 0.009158865 |
| IGKV3D_11 | Eicosapentaenoic.acid | -0.386401099 | 0.001611883 | 0.004575668 |
| IGKV3D_20 | Eicosapentaenoic.acid | -0.390201465 | 0.001435271 | 0.004325473 |
| MMP7 | Eicosapentaenoic.acid | -0.230860806 | 0.066451664 | 0.078388022 |
| MMP9 | Eicosapentaenoic.acid | -0.192445055 | 0.127634466 | 0.140749787 |
| TPH1 | Eicosapentaenoic.acid | 0.334065934 | 0.006979185 | 0.013226273 |
| CASQ2 | Decanoyl_L_carnitine | 0.446978022 | 0.000213354 | 0.00093876 |
| CD36 | Decanoyl_L_carnitine | -0.353708791 | 0.004141016 | 0.009343831 |
| CNTN1 | Decanoyl_L_carnitine | 0.416483516 | 0.000618996 | 0.002288723 |
| CNTN4 | Decanoyl_L_carnitine | 0.429652354 | 0.000395584 | 0.001582334 |
| FABP4 | Decanoyl_L_carnitine | -0.433424908 | 0.000346789 | 0.001412843 |
| IBSP | Decanoyl_L_carnitine | -0.40764652 | 0.000827597 | 0.002913142 |
| IGHV3_43 | Decanoyl_L_carnitine | -0.362225275 | 0.003268605 | 0.007858942 |
| IGHV3_52 | Decanoyl_L_carnitine | -0.371657509 | 0.002496443 | 0.006454399 |
| IGHV4_59 | Decanoyl_L_carnitine | -0.359249084 | 0.003552882 | 0.008404667 |
| IGJ | Decanoyl_L_carnitine | -0.3875 | 0.00155891 | 0.004512635 |
| IGKC | Decanoyl_L_carnitine | -0.334249084 | 0.006946334 | 0.013226273 |
| IGKV1D_27 | Decanoyl_L_carnitine | -0.335485348 | 0.006728111 | 0.013041273 |
| IGKV1D_33 | Decanoyl_L_carnitine | -0.348031136 | 0.004831568 | 0.010576567 |
| IGKV1OR2_3 | Decanoyl_L_carnitine | -0.341346154 | 0.005773335 | 0.011815196 |
| IGKV2D_26 | Decanoyl_L_carnitine | -0.321886447 | 0.009493052 | 0.016842512 |
| IGKV3D_11 | Decanoyl_L_carnitine | -0.33489011 | 0.00683242 | 0.013070716 |
| IGKV3D_20 | Decanoyl_L_carnitine | -0.322619048 | 0.00932216 | 0.016606276 |
| MMP7 | Decanoyl_L_carnitine | -0.339468864 | 0.006065293 | 0.012241876 |
| MMP9 | Decanoyl_L_carnitine | -0.314514652 | 0.011370056 | 0.019022148 |
| TPH1 | Decanoyl_L_carnitine | 0.448992674 | 0.000198145 | 0.000917726 |
| CASQ2 | X3_Methoxy_4_Hydroxyphenylglycol.Sulfate | -0.294871795 | 0.018013739 | 0.027616882 |
| CD36 | X3_Methoxy_4_Hydroxyphenylglycol.Sulfate | 0.191849817 | 0.128839331 | 0.141723264 |
| CNTN1 | X3_Methoxy_4_Hydroxyphenylglycol.Sulfate | -0.189194139 | 0.134320131 | 0.146652252 |
| CNTN4 | X3_Methoxy_4_Hydroxyphenylglycol.Sulfate | -0.213257936 | 0.090653074 | 0.102275263 |
| FABP4 | X3_Methoxy_4_Hydroxyphenylglycol.Sulfate | 0.23992674 | 0.056187552 | 0.067919019 |
| IBSP | X3_Methoxy_4_Hydroxyphenylglycol.Sulfate | 0.311721612 | 0.012160894 | 0.019957001 |
| IGHV3_43 | X3_Methoxy_4_Hydroxyphenylglycol.Sulfate | 0.274084249 | 0.028407404 | 0.03993373 |
| IGHV3_52 | X3_Methoxy_4_Hydroxyphenylglycol.Sulfate | 0.265705128 | 0.033833306 | 0.046088714 |
| IGHV4_59 | X3_Methoxy_4_Hydroxyphenylglycol.Sulfate | 0.262271062 | 0.036294094 | 0.048539214 |
| IGJ | X3_Methoxy_4_Hydroxyphenylglycol.Sulfate | 0.331639194 | 0.007427487 | 0.013731489 |
| IGKC | X3_Methoxy_4_Hydroxyphenylglycol.Sulfate | 0.283562271 | 0.023170426 | 0.034051325 |
| IGKV1D_27 | X3_Methoxy_4_Hydroxyphenylglycol.Sulfate | 0.250549451 | 0.045839837 | 0.058125441 |
| IGKV1D_33 | X3_Methoxy_4_Hydroxyphenylglycol.Sulfate | 0.296520147 | 0.017350992 | 0.027072469 |
| IGKV1OR2_3 | X3_Methoxy_4_Hydroxyphenylglycol.Sulfate | 0.303434066 | 0.014793481 | 0.023669569 |
| IGKV2D_26 | X3_Methoxy_4_Hydroxyphenylglycol.Sulfate | 0.225961538 | 0.072595886 | 0.08462597 |
| IGKV3D_11 | X3_Methoxy_4_Hydroxyphenylglycol.Sulfate | 0.286950549 | 0.021508462 | 0.032036774 |
| IGKV3D_20 | X3_Methoxy_4_Hydroxyphenylglycol.Sulfate | 0.291620879 | 0.019384388 | 0.029615036 |
| MMP7 | X3_Methoxy_4_Hydroxyphenylglycol.Sulfate | 0.211858974 | 0.092839952 | 0.104474626 |
| MMP9 | X3_Methoxy_4_Hydroxyphenylglycol.Sulfate | 0.208608059 | 0.098081348 | 0.10953247 |
| TPH1 | X3_Methoxy_4_Hydroxyphenylglycol.Sulfate | -0.277106227 | 0.02663916 | 0.038179904 |
| CASQ2 | O_Desmethylnaproxen | 0.354990842 | 0.003997748 | 0.009114037 |
| CD36 | O_Desmethylnaproxen | -0.249587912 | 0.046706829 | 0.058602043 |
| CNTN1 | O_Desmethylnaproxen | 0.312362637 | 0.011975305 | 0.019734586 |
| CNTN4 | O_Desmethylnaproxen | 0.295447521 | 0.017779842 | 0.027546234 |
| FABP4 | O_Desmethylnaproxen | -0.343681319 | 0.005427467 | 0.011211669 |
| IBSP | O_Desmethylnaproxen | -0.358836996 | 0.003593925 | 0.008456294 |
| IGHV3_43 | O_Desmethylnaproxen | -0.30782967 | 0.013341923 | 0.021503466 |
| IGHV3_52 | O_Desmethylnaproxen | -0.3128663 | 0.011831209 | 0.019644271 |
| IGHV4_59 | O_Desmethylnaproxen | -0.295054945 | 0.017939048 | 0.027598535 |
| IGJ | O_Desmethylnaproxen | -0.308012821 | 0.013284206 | 0.021489157 |
| IGKC | O_Desmethylnaproxen | -0.278342491 | 0.025943003 | 0.037425972 |
| IGKV1D_27 | O_Desmethylnaproxen | -0.349725275 | 0.004615591 | 0.0101543 |
| IGKV1D_33 | O_Desmethylnaproxen | -0.315659341 | 0.011059044 | 0.018787565 |
| IGKV1OR2_3 | O_Desmethylnaproxen | -0.311584249 | 0.012200985 | 0.019957001 |
| IGKV2D_26 | O_Desmethylnaproxen | -0.286034799 | 0.021947336 | 0.032405462 |
| IGKV3D_11 | O_Desmethylnaproxen | -0.270467033 | 0.030652624 | 0.042147357 |
| IGKV3D_20 | O_Desmethylnaproxen | -0.287408425 | 0.021291836 | 0.031865332 |
| MMP7 | O_Desmethylnaproxen | -0.2746337 | 0.028078762 | 0.039675184 |
| MMP9 | O_Desmethylnaproxen | -0.223443223 | 0.075926162 | 0.087683757 |
| TPH1 | O_Desmethylnaproxen | 0.258287546 | 0.039333215 | 0.051661536 |
| CASQ2 | Salicylic.acid | -0.308333333 | 0.013183719 | 0.0214053 |
| CD36 | Salicylic.acid | 0.330586081 | 0.007629744 | 0.013987864 |
| CNTN1 | Salicylic.acid | -0.317032967 | 0.010695584 | 0.018383035 |
| CNTN4 | Salicylic.acid | -0.315983471 | 0.010972328 | 0.018712498 |
| FABP4 | Salicylic.acid | 0.244826007 | 0.051201128 | 0.06333775 |
| IBSP | Salicylic.acid | 0.325091575 | 0.008765007 | 0.015936377 |
| IGHV3_43 | Salicylic.acid | 0.227518315 | 0.07059621 | 0.082832886 |
| IGHV3_52 | Salicylic.acid | 0.241300366 | 0.05475127 | 0.066918219 |
| IGHV4_59 | Salicylic.acid | 0.252335165 | 0.044264799 | 0.056617766 |
| IGJ | Salicylic.acid | 0.256501832 | 0.040762433 | 0.053063523 |
| IGKC | Salicylic.acid | 0.222161172 | 0.077667677 | 0.089226575 |
| IGKV1D_27 | Salicylic.acid | 0.219368132 | 0.081571565 | 0.093224645 |
| IGKV1D_33 | Salicylic.acid | 0.241025641 | 0.055036111 | 0.067080025 |
| IGKV1OR2_3 | Salicylic.acid | 0.256868132 | 0.040465812 | 0.052833701 |
| IGKV2D_26 | Salicylic.acid | 0.240201465 | 0.055897874 | 0.067754999 |
| IGKV3D_11 | Salicylic.acid | 0.189377289 | 0.133936589 | 0.146597261 |
| IGKV3D_20 | Salicylic.acid | 0.210302198 | 0.095321864 | 0.10672168 |
| MMP7 | Salicylic.acid | 0.389423077 | 0.001469958 | 0.004370147 |
| MMP9 | Salicylic.acid | 0.371382784 | 0.002516399 | 0.006454399 |
| TPH1 | Salicylic.acid | -0.273809524 | 0.028572933 | 0.040038505 |
| CASQ2 | X3_Aminopropanesulphonic.Acid | 0.034340659 | 0.787629501 | 0.80407652 |
| CD36 | X3_Aminopropanesulphonic.Acid | 0.064194139 | 0.614295322 | 0.632997522 |
| CNTN1 | X3_Aminopropanesulphonic.Acid | 0.098305861 | 0.439628147 | 0.461662016 |
| CNTN4 | X3_Aminopropanesulphonic.Acid | 0.002037569 | 0.987250912 | 0.987250912 |
| FABP4 | X3_Aminopropanesulphonic.Acid | -0.003067766 | 0.980805973 | 0.983040156 |
| IBSP | X3_Aminopropanesulphonic.Acid | 0.141163004 | 0.26586787 | 0.286019224 |
| IGHV3_43 | X3_Aminopropanesulphonic.Acid | -0.071703297 | 0.573403425 | 0.592247669 |
| IGHV3_52 | X3_Aminopropanesulphonic.Acid | -0.056913919 | 0.655091742 | 0.671888966 |
| IGHV4_59 | X3_Aminopropanesulphonic.Acid | -0.081456044 | 0.522257433 | 0.543246502 |
| IGJ | X3_Aminopropanesulphonic.Acid | 0.108836996 | 0.39195224 | 0.414564869 |
| IGKC | X3_Aminopropanesulphonic.Acid | -0.027472527 | 0.829385961 | 0.844744961 |
| IGKV1D_27 | X3_Aminopropanesulphonic.Acid | -0.003479853 | 0.978228259 | 0.982695054 |
| IGKV1D_33 | X3_Aminopropanesulphonic.Acid | -0.037637363 | 0.76778831 | 0.785643852 |
| IGKV1OR2_3 | X3_Aminopropanesulphonic.Acid | -0.025412088 | 0.842010844 | 0.855623029 |
| IGKV2D_26 | X3_Aminopropanesulphonic.Acid | -0.078754579 | 0.536192388 | 0.556426063 |
| IGKV3D_11 | X3_Aminopropanesulphonic.Acid | -0.006730769 | 0.957902842 | 0.966690941 |
| IGKV3D_20 | X3_Aminopropanesulphonic.Acid | -0.011263736 | 0.929609098 | 0.94029426 |
| MMP7 | X3_Aminopropanesulphonic.Acid | 0.095879121 | 0.45105694 | 0.471413429 |
| MMP9 | X3_Aminopropanesulphonic.Acid | 0.005631868 | 0.964771063 | 0.971394205 |
| TPH1 | X3_Aminopropanesulphonic.Acid | 0.024862637 | 0.845384321 | 0.857071662 |
| CASQ2 | X6_Hydroxynicotinic.acid | -0.11753663 | 0.354980402 | 0.377273857 |
| CD36 | X6_Hydroxynicotinic.acid | 0.274542125 | 0.028133312 | 0.039675184 |
| CNTN1 | X6_Hydroxynicotinic.acid | -0.112225275 | 0.377289842 | 0.400018146 |
| CNTN4 | X6_Hydroxynicotinic.acid | -0.190890464 | 0.130799345 | 0.143520478 |
| FABP4 | X6_Hydroxynicotinic.acid | 0.131913919 | 0.298776302 | 0.320637983 |
| IBSP | X6_Hydroxynicotinic.acid | 0.145512821 | 0.251266794 | 0.271639777 |
| IGHV3_43 | X6_Hydroxynicotinic.acid | 0.076739927 | 0.546702042 | 0.565997408 |
| IGHV3_52 | X6_Hydroxynicotinic.acid | 0.101236264 | 0.426046079 | 0.448469556 |
| IGHV4_59 | X6_Hydroxynicotinic.acid | 0.083836996 | 0.51012819 | 0.531887212 |
| IGJ | X6_Hydroxynicotinic.acid | 0.210302198 | 0.095321864 | 0.10672168 |
| IGKC | X6_Hydroxynicotinic.acid | 0.118223443 | 0.352156115 | 0.375178428 |
| IGKV1D_27 | X6_Hydroxynicotinic.acid | 0.097847985 | 0.441772025 | 0.462808788 |
| IGKV1D_33 | X6_Hydroxynicotinic.acid | 0.103800366 | 0.414360247 | 0.437214649 |
| IGKV1OR2_3 | X6_Hydroxynicotinic.acid | 0.143360806 | 0.258420786 | 0.278689083 |
| IGKV2D_26 | X6_Hydroxynicotinic.acid | 0.057600733 | 0.651197355 | 0.669455225 |
| IGKV3D_11 | X6_Hydroxynicotinic.acid | 0.123351648 | 0.331508974 | 0.35403871 |
| IGKV3D_20 | X6_Hydroxynicotinic.acid | 0.125320513 | 0.323789134 | 0.346635569 |
| MMP7 | X6_Hydroxynicotinic.acid | 0.251007326 | 0.045431656 | 0.05777436 |
| MMP9 | X6_Hydroxynicotinic.acid | 0.221062271 | 0.079185533 | 0.090733423 |
| TPH1 | X6_Hydroxynicotinic.acid | -0.154304029 | 0.223448465 | 0.242160898 |
| CASQ2 | Formylanthranilic.acid | -0.356089744 | 0.003878459 | 0.008921906 |
| CD36 | Formylanthranilic.acid | 0.318772894 | 0.010250108 | 0.017686461 |
| CNTN1 | Formylanthranilic.acid | -0.415842491 | 0.000632343 | 0.002318592 |
| CNTN4 | Formylanthranilic.acid | -0.418777688 | 0.000573293 | 0.002155975 |
| FABP4 | Formylanthranilic.acid | 0.405723443 | 0.000880673 | 0.003003846 |
| IBSP | Formylanthranilic.acid | 0.447069597 | 0.000212641 | 0.00093876 |
| IGHV3_43 | Formylanthranilic.acid | 0.346703297 | 0.005007033 | 0.010694634 |
| IGHV3_52 | Formylanthranilic.acid | 0.35 | 0.004581383 | 0.0101543 |
| IGHV4_59 | Formylanthranilic.acid | 0.313965201 | 0.011522013 | 0.019203356 |
| IGJ | Formylanthranilic.acid | 0.349908425 | 0.004592761 | 0.0101543 |
| IGKC | Formylanthranilic.acid | 0.338827839 | 0.006167916 | 0.012335832 |
| IGKV1D_27 | Formylanthranilic.acid | 0.29757326 | 0.016938582 | 0.0266344 |
| IGKV1D_33 | Formylanthranilic.acid | 0.357005495 | 0.003781472 | 0.008803427 |
| IGKV1OR2_3 | Formylanthranilic.acid | 0.38489011 | 0.001687359 | 0.004759216 |
| IGKV2D_26 | Formylanthranilic.acid | 0.308333333 | 0.013183719 | 0.0214053 |
| IGKV3D_11 | Formylanthranilic.acid | 0.344826007 | 0.005264689 | 0.011030776 |
| IGKV3D_20 | Formylanthranilic.acid | 0.344413919 | 0.005322786 | 0.01109965 |
| MMP7 | Formylanthranilic.acid | 0.25 | 0.046333627 | 0.058571381 |
| MMP9 | Formylanthranilic.acid | 0.272069597 | 0.029640211 | 0.041051091 |
| TPH1 | Formylanthranilic.acid | -0.316575092 | 0.010815569 | 0.018516927 |
| CASQ2 | Xanthopterin | 0.498580586 | 2.75E-05 | 0.00035796 |
| CD36 | Xanthopterin | -0.288827839 | 0.020632035 | 0.031303777 |
| CNTN1 | Xanthopterin | 0.44217033 | 0.000254066 | 0.001085329 |
| CNTN4 | Xanthopterin | 0.425623004 | 0.000454542 | 0.001785703 |
| FABP4 | Xanthopterin | -0.319230769 | 0.01013559 | 0.017557715 |
| IBSP | Xanthopterin | -0.416941392 | 0.000609618 | 0.002273153 |
| IGHV3_43 | Xanthopterin | -0.386538462 | 0.001605174 | 0.004575668 |
| IGHV3_52 | Xanthopterin | -0.388782051 | 0.001499089 | 0.004397327 |
| IGHV4_59 | Xanthopterin | -0.400228938 | 0.001049707 | 0.003421266 |
| IGJ | Xanthopterin | -0.320695971 | 0.009776567 | 0.017205649 |
| IGKC | Xanthopterin | -0.363736264 | 0.003132167 | 0.007614107 |
| IGKV1D_27 | Xanthopterin | -0.353296703 | 0.004188022 | 0.009401681 |
| IGKV1D_33 | Xanthopterin | -0.394230769 | 0.001267201 | 0.003954387 |
| IGKV1OR2_3 | Xanthopterin | -0.379349817 | 0.001991981 | 0.00541032 |
| IGKV2D_26 | Xanthopterin | -0.367673993 | 0.002800097 | 0.007000243 |
| IGKV3D_11 | Xanthopterin | -0.356776557 | 0.003805515 | 0.008812771 |
| IGKV3D_20 | Xanthopterin | -0.360851648 | 0.003397196 | 0.008079819 |
| MMP7 | Xanthopterin | -0.272527473 | 0.029356157 | 0.040875662 |
| MMP9 | Xanthopterin | -0.320421245 | 0.009843029 | 0.017205649 |
| TPH1 | Xanthopterin | 0.365567766 | 0.002973593 | 0.00730939 |
| CASQ2 | N1_Methyl_4_pyridone_3_carboxamide | 0.573305861 | 7.37E-07 | 4.50591E-05 |
| CD36 | N1_Methyl_4_pyridone_3_carboxamide | -0.499725275 | 2.62E-05 | 0.00035796 |
| CNTN1 | N1_Methyl_4_pyridone_3_carboxamide | 0.583836996 | 4.11E-07 | 3.61491E-05 |
| CNTN4 | N1_Methyl_4_pyridone_3_carboxamide | 0.578120171 | 5.66E-07 | 4.14805E-05 |
| FABP4 | N1_Methyl_4_pyridone_3_carboxamide | -0.589331502 | 3.00E-07 | 3.61491E-05 |
| IBSP | N1_Methyl_4_pyridone_3_carboxamide | -0.569276557 | 9.17E-07 | 4.50591E-05 |
| IGHV3_43 | N1_Methyl_4_pyridone_3_carboxamide | -0.496703297 | 2.98E-05 | 0.00035796 |
| IGHV3_52 | N1_Methyl_4_pyridone_3_carboxamide | -0.506272894 | 1.97E-05 | 0.000329157 |
| IGHV4_59 | N1_Methyl_4_pyridone_3_carboxamide | -0.486446886 | 4.59E-05 | 0.000415749 |
| IGJ | N1_Methyl_4_pyridone_3_carboxamide | -0.472619048 | 8.04E-05 | 0.00052378 |
| IGKC | N1_Methyl_4_pyridone_3_carboxamide | -0.489285714 | 4.08E-05 | 0.000399093 |
| IGKV1D_27 | N1_Methyl_4_pyridone_3_carboxamide | -0.471565934 | 8.38E-05 | 0.000526617 |
| IGKV1D_33 | N1_Methyl_4_pyridone_3_carboxamide | -0.51959707 | 1.08E-05 | 0.000238416 |
| IGKV1OR2_3 | N1_Methyl_4_pyridone_3_carboxamide | -0.503891941 | 2.19E-05 | 0.000332041 |
| IGKV2D_26 | N1_Methyl_4_pyridone_3_carboxamide | -0.44793956 | 0.000205967 | 0.00092475 |
| IGKV3D_11 | N1_Methyl_4_pyridone_3_carboxamide | -0.462042125 | 0.000121353 | 0.000635656 |
| IGKV3D_20 | N1_Methyl_4_pyridone_3_carboxamide | -0.472435897 | 8.09E-05 | 0.00052378 |
| MMP7 | N1_Methyl_4_pyridone_3_carboxamide | -0.512728938 | 1.48E-05 | 0.000271327 |
| MMP9 | N1_Methyl_4_pyridone_3_carboxamide | -0.493269231 | 3.45E-05 | 0.000379856 |
| TPH1 | N1_Methyl_4_pyridone_3_carboxamide | 0.55297619 | 2.15E-06 | 7.90044E-05 |
| CASQ2 | X3_Hydroxydodecanoic.acid | 0.402472527 | 0.000977434 | 0.003233616 |
| CD36 | X3_Hydroxydodecanoic.acid | -0.324404762 | 0.008916782 | 0.016145614 |
| CNTN1 | X3_Hydroxydodecanoic.acid | 0.353021978 | 0.004219621 | 0.009424533 |
| CNTN4 | X3_Hydroxydodecanoic.acid | 0.379628888 | 0.001975533 | 0.005398972 |
| FABP4 | X3_Hydroxydodecanoic.acid | -0.448260073 | 0.000203557 | 0.000923352 |
| IBSP | X3_Hydroxydodecanoic.acid | -0.381181319 | 0.001886232 | 0.005187138 |
| IGHV3_43 | X3_Hydroxydodecanoic.acid | -0.251465201 | 0.045026468 | 0.05742506 |
| IGHV3_52 | X3_Hydroxydodecanoic.acid | -0.264835165 | 0.034443148 | 0.046774646 |
| IGHV4_59 | X3_Hydroxydodecanoic.acid | -0.260897436 | 0.037319219 | 0.049447862 |
| IGJ | X3_Hydroxydodecanoic.acid | -0.263965201 | 0.035062124 | 0.047323112 |
| IGKC | X3_Hydroxydodecanoic.acid | -0.255631868 | 0.041474119 | 0.053830715 |
| IGKV1D_27 | X3_Hydroxydodecanoic.acid | -0.249862637 | 0.046457755 | 0.058571381 |
| IGKV1D_33 | X3_Hydroxydodecanoic.acid | -0.271382784 | 0.030070614 | 0.041476709 |
| IGKV1OR2_3 | X3_Hydroxydodecanoic.acid | -0.263324176 | 0.035524115 | 0.047800033 |
| IGKV2D_26 | X3_Hydroxydodecanoic.acid | -0.242261905 | 0.053763755 | 0.066078358 |
| IGKV3D_11 | X3_Hydroxydodecanoic.acid | -0.219139194 | 0.08189832 | 0.093355598 |
| IGKV3D_20 | X3_Hydroxydodecanoic.acid | -0.232005495 | 0.065078096 | 0.077181569 |
| MMP7 | X3_Hydroxydodecanoic.acid | -0.343727106 | 0.005420872 | 0.011211669 |
| MMP9 | X3_Hydroxydodecanoic.acid | -0.333928571 | 0.007003913 | 0.013226273 |
| TPH1 | X3_Hydroxydodecanoic.acid | 0.373397436 | 0.002373297 | 0.006232391 |
| CASQ2 | Salicyluric.acid | -0.217307692 | 0.084549794 | 0.096128964 |
| CD36 | Salicyluric.acid | 0.273351648 | 0.028850616 | 0.040299273 |
| CNTN1 | Salicyluric.acid | -0.225274725 | 0.073492354 | 0.08509641 |
| CNTN4 | Salicyluric.acid | -0.194141416 | 0.124247635 | 0.137705187 |
| FABP4 | Salicyluric.acid | 0.161309524 | 0.202876074 | 0.220408574 |
| IBSP | Salicyluric.acid | 0.248534799 | 0.047671792 | 0.05958974 |
| IGHV3_43 | Salicyluric.acid | 0.201648352 | 0.110075535 | 0.122615786 |
| IGHV3_52 | Salicyluric.acid | 0.216300366 | 0.08603672 | 0.097470645 |
| IGHV4_59 | Salicyluric.acid | 0.243269231 | 0.052744824 | 0.065007626 |
| IGJ | Salicyluric.acid | 0.252655678 | 0.043986872 | 0.056426308 |
| IGKC | Salicyluric.acid | 0.234661172 | 0.061979317 | 0.074105705 |
| IGKV1D_27 | Salicyluric.acid | 0.249542125 | 0.046748448 | 0.058602043 |
| IGKV1D_33 | Salicyluric.acid | 0.245558608 | 0.050487558 | 0.062752897 |
| IGKV1OR2_3 | Salicyluric.acid | 0.289606227 | 0.02027765 | 0.030872546 |
| IGKV2D_26 | Salicyluric.acid | 0.180082418 | 0.154465041 | 0.168229252 |
| IGKV3D_11 | Salicyluric.acid | 0.216208791 | 0.086172911 | 0.097470645 |
| IGKV3D_20 | Salicyluric.acid | 0.225778388 | 0.072834086 | 0.08462597 |
| MMP7 | Salicyluric.acid | 0.346840659 | 0.004988625 | 0.010694634 |
| MMP9 | Salicyluric.acid | 0.286309524 | 0.021814883 | 0.032318345 |
| TPH1 | Salicyluric.acid | -0.238369963 | 0.057852179 | 0.069549068 |
| CASQ2 | Phenylacetylglycine | -0.362225275 | 0.003268605 | 0.007858942 |
| CD36 | Phenylacetylglycine | 0.288598901 | 0.020737263 | 0.031355312 |
| CNTN1 | Phenylacetylglycine | -0.429761905 | 0.000394083 | 0.001582334 |
| CNTN4 | Phenylacetylglycine | -0.423264918 | 0.000492651 | 0.001871897 |
| FABP4 | Phenylacetylglycine | 0.283241758 | 0.023333123 | 0.03410822 |
| IBSP | Phenylacetylglycine | 0.38209707 | 0.001835267 | 0.005078727 |
| IGHV3_43 | Phenylacetylglycine | 0.399587912 | 0.001071224 | 0.003465723 |
| IGHV3_52 | Phenylacetylglycine | 0.397847985 | 0.001131647 | 0.003634487 |
| IGHV4_59 | Phenylacetylglycine | 0.367994505 | 0.002774502 | 0.00697589 |
| IGJ | Phenylacetylglycine | 0.357967033 | 0.003681955 | 0.008617342 |
| IGKC | Phenylacetylglycine | 0.39235348 | 0.001343166 | 0.004104119 |
| IGKV1D_27 | Phenylacetylglycine | 0.403250916 | 0.00095343 | 0.003220693 |
| IGKV1D_33 | Phenylacetylglycine | 0.383012821 | 0.00178554 | 0.00497239 |
| IGKV1OR2_3 | Phenylacetylglycine | 0.401877289 | 0.000996157 | 0.003270963 |
| IGKV2D_26 | Phenylacetylglycine | 0.368177656 | 0.002759969 | 0.00697589 |
| IGKV3D_11 | Phenylacetylglycine | 0.383882784 | 0.001739422 | 0.004874813 |
| IGKV3D_20 | Phenylacetylglycine | 0.396108059 | 0.001195127 | 0.003810318 |
| MMP7 | Phenylacetylglycine | 0.240201465 | 0.055897874 | 0.067754999 |
| MMP9 | Phenylacetylglycine | 0.304212454 | 0.014526959 | 0.023327964 |
| TPH1 | Phenylacetylglycine | -0.376144689 | 0.002189876 | 0.005875277 |
| CASQ2 | D_Biotin | 0.517765568 | 1.18E-05 | 0.000242158 |
| CD36 | D_Biotin | -0.393086081 | 0.001313047 | 0.004040145 |
| CNTN1 | D_Biotin | 0.496108059 | 3.06E-05 | 0.00035796 |
| CNTN4 | D_Biotin | 0.488009249 | 4.30E-05 | 0.000411723 |
| FABP4 | D_Biotin | -0.441895604 | 0.000256594 | 0.001085589 |
| IBSP | D_Biotin | -0.467490842 | 9.83E-05 | 0.000573888 |
| IGHV3_43 | D_Biotin | -0.504075092 | 2.17E-05 | 0.000332041 |
| IGHV3_52 | D_Biotin | -0.51717033 | 1.21E-05 | 0.000242158 |
| IGHV4_59 | D_Biotin | -0.495879121 | 3.09E-05 | 0.00035796 |
| IGJ | D_Biotin | -0.465018315 | 0.000108211 | 0.000602619 |
| IGKC | D_Biotin | -0.505723443 | 2.02E-05 | 0.000329157 |
| IGKV1D_27 | D_Biotin | -0.491712454 | 3.69E-05 | 0.000388525 |
| IGKV1D_33 | D_Biotin | -0.524954212 | 8.46E-06 | 0.000195881 |
| IGKV1OR2_3 | D_Biotin | -0.500869963 | 2.50E-05 | 0.000354203 |
| IGKV2D_26 | D_Biotin | -0.461034799 | 0.000126123 | 0.000645718 |
| IGKV3D_11 | D_Biotin | -0.506318681 | 1.97E-05 | 0.000329157 |
| IGKV3D_20 | D_Biotin | -0.515796703 | 1.29E-05 | 0.00024654 |
| MMP7 | D_Biotin | -0.367078755 | 0.00284819 | 0.007080247 |
| MMP9 | D_Biotin | -0.389148352 | 0.00148238 | 0.004377498 |
| TPH1 | D_Biotin | 0.487179487 | 4.46E-05 | 0.000415749 |
| CASQ2 | X5_10_methylene_THF | 0.455128205 | 0.000157737 | 0.000771159 |
| CD36 | X5_10_methylene_THF | -0.3375 | 0.006385355 | 0.012644037 |
| CNTN1 | X5_10_methylene_THF | 0.395879121 | 0.001203714 | 0.003810318 |
| CNTN4 | X5_10_methylene_THF | 0.40307238 | 0.000958888 | 0.003220693 |
| FABP4 | X5_10_methylene_THF | -0.335210623 | 0.006776079 | 0.013070716 |
| IBSP | X5_10_methylene_THF | -0.476968864 | 6.76E-05 | 0.000485901 |
| IGHV3_43 | X5_10_methylene_THF | -0.327838828 | 0.008180198 | 0.0149348 |
| IGHV3_52 | X5_10_methylene_THF | -0.345833333 | 0.005125018 | 0.010893759 |
| IGHV4_59 | X5_10_methylene_THF | -0.320421245 | 0.009843029 | 0.017205649 |
| IGJ | X5_10_methylene_THF | -0.314606227 | 0.0113449 | 0.019022148 |
| IGKC | X5_10_methylene_THF | -0.323992674 | 0.009008942 | 0.016197806 |
| IGKV1D_27 | X5_10_methylene_THF | -0.298260073 | 0.016674165 | 0.026390764 |
| IGKV1D_33 | X5_10_methylene_THF | -0.336721612 | 0.006515922 | 0.012742248 |
| IGKV1OR2_3 | X5_10_methylene_THF | -0.29739011 | 0.017009697 | 0.0266344 |
| IGKV2D_26 | X5_10_methylene_THF | -0.320054945 | 0.009932256 | 0.017273489 |
| IGKV3D_11 | X5_10_methylene_THF | -0.336172161 | 0.006609493 | 0.012868039 |
| IGKV3D_20 | X5_10_methylene_THF | -0.332509158 | 0.00726396 | 0.013542976 |
| MMP7 | X5_10_methylene_THF | -0.261538462 | 0.036837874 | 0.049014251 |
| MMP9 | X5_10_methylene_THF | -0.355952381 | 0.003893196 | 0.008921906 |
| TPH1 | X5_10_methylene_THF | 0.388507326 | 0.001511731 | 0.004405044 |
| CASQ2 | g__Gemella | 0.082235392 | 0.518271358 | 0.651541136 |
| CD36 | g__Gemella | -0.046659508 | 0.714276576 | 0.818441911 |
| CNTN1 | g__Gemella | 0.067743872 | 0.594808269 | 0.717403186 |
| CNTN4 | g__Gemella | 0.073573172 | 0.563420255 | 0.692471821 |
| FABP4 | g__Gemella | -0.000764388 | 0.995217048 | 0.995217048 |
| IBSP | g__Gemella | -0.021625806 | 0.865311809 | 0.910854536 |
| IGHV3_43 | g__Gemella | -0.172178361 | 0.173683275 | 0.400363592 |
| IGHV3_52 | g__Gemella | -0.18354863 | 0.146553138 | 0.396088009 |
| IGHV4_59 | g__Gemella | -0.139978523 | 0.269940583 | 0.435087235 |
| IGJ | g__Gemella | -0.129882234 | 0.306345794 | 0.455378882 |
| IGKC | g__Gemella | -0.143896011 | 0.256628859 | 0.434294992 |
| IGKV1D_27 | g__Gemella | -0.231099923 | 0.066162827 | 0.353841981 |
| IGKV1D_33 | g__Gemella | -0.174981116 | 0.166678667 | 0.400363592 |
| IGKV1OR2_3 | g__Gemella | -0.154788537 | 0.221980481 | 0.420276528 |
| IGKV2D_26 | g__Gemella | -0.164789278 | 0.193173637 | 0.417003605 |
| IGKV3D_11 | g__Gemella | -0.137940155 | 0.27704657 | 0.435087235 |
| IGKV3D_20 | g__Gemella | -0.160011854 | 0.206581475 | 0.420276528 |
| MMP7 | g__Gemella | -0.041213244 | 0.746433874 | 0.837833941 |
| MMP9 | g__Gemella | -0.054494483 | 0.668882972 | 0.778593936 |
| TPH1 | g__Gemella | 0.043219762 | 0.734532892 | 0.832975445 |
| CASQ2 | g__Lactobacillus | -0.207195727 | 0.100429057 | 0.353841981 |
| CD36 | g__Lactobacillus | 0.253657019 | 0.043127822 | 0.353841981 |
| CNTN1 | g__Lactobacillus | -0.208303175 | 0.0985845 | 0.353841981 |
| CNTN4 | g__Lactobacillus | -0.220075544 | 0.080568414 | 0.353841981 |
| FABP4 | g__Lactobacillus | 0.319253975 | 0.010129816 | 0.353841981 |
| IBSP | g__Lactobacillus | 0.219660952 | 0.081155137 | 0.353841981 |
| IGHV3_43 | g__Lactobacillus | 0.181080566 | 0.152154927 | 0.397388979 |
| IGHV3_52 | g__Lactobacillus | 0.171525611 | 0.175344978 | 0.400363592 |
| IGHV4_59 | g__Lactobacillus | 0.206191298 | 0.102125134 | 0.353841981 |
| IGJ | g__Lactobacillus | 0.210337788 | 0.095264551 | 0.353841981 |
| IGKC | g__Lactobacillus | 0.163206877 | 0.197543716 | 0.420276528 |
| IGKV1D_27 | g__Lactobacillus | 0.116075965 | 0.361033106 | 0.510926762 |
| IGKV1D_33 | g__Lactobacillus | 0.158339258 | 0.211427814 | 0.420276528 |
| IGKV1OR2_3 | g__Lactobacillus | 0.223446877 | 0.075921244 | 0.353841981 |
| IGKV2D_26 | g__Lactobacillus | 0.161095 | 0.203485351 | 0.420276528 |
| IGKV3D_11 | g__Lactobacillus | 0.154244277 | 0.223629974 | 0.420276528 |
| IGKV3D_20 | g__Lactobacillus | 0.154424559 | 0.223082647 | 0.420276528 |
| MMP7 | g__Lactobacillus | 0.256541533 | 0.040730197 | 0.353841981 |
| MMP9 | g__Lactobacillus | 0.279797934 | 0.02514315 | 0.353841981 |
| TPH1 | g__Lactobacillus | -0.183012161 | 0.147757477 | 0.396088009 |
| CASQ2 | g__Christensenella | -0.011596965 | 0.927532154 | 0.940355179 |
| CD36 | g__Christensenella | 0.308335804 | 0.013182947 | 0.353841981 |
| CNTN1 | g__Christensenella | -0.059778171 | 0.638912449 | 0.755702897 |
| CNTN4 | g__Christensenella | -0.019806727 | 0.876548542 | 0.913936868 |
| FABP4 | g__Christensenella | 0.068944157 | 0.588281941 | 0.715038823 |
| IBSP | g__Christensenella | 0.166820948 | 0.187664984 | 0.412862966 |
| IGHV3_43 | g__Christensenella | 0.170567047 | 0.177806121 | 0.400363592 |
| IGHV3_52 | g__Christensenella | 0.176544864 | 0.162861767 | 0.400363592 |
| IGHV4_59 | g__Christensenella | 0.150800399 | 0.234265671 | 0.420276528 |
| IGJ | g__Christensenella | 0.193123343 | 0.126271937 | 0.360776963 |
| IGKC | g__Christensenella | 0.151796701 | 0.231153499 | 0.420276528 |
| IGKV1D_27 | g__Christensenella | 0.242978338 | 0.053037438 | 0.353841981 |
| IGKV1D_33 | g__Christensenella | 0.158013631 | 0.212380563 | 0.420276528 |
| IGKV1OR2_3 | g__Christensenella | 0.171842315 | 0.174537307 | 0.400363592 |
| IGKV2D_26 | g__Christensenella | 0.154068272 | 0.224165218 | 0.420276528 |
| IGKV3D_11 | g__Christensenella | 0.149286018 | 0.23905143 | 0.420730516 |
| IGKV3D_20 | g__Christensenella | 0.15761511 | 0.213550707 | 0.420276528 |
| MMP7 | g__Christensenella | 0.245369465 | 0.050671005 | 0.353841981 |
| MMP9 | g__Christensenella | 0.202608147 | 0.108357304 | 0.353841981 |
| TPH1 | g__Christensenella | -0.112064144 | 0.377979554 | 0.522141965 |
| CASQ2 | g__Parvimonas | 0.01221691 | 0.923669461 | 0.940355179 |
| CD36 | g__Parvimonas | -0.035967797 | 0.777818909 | 0.8556008 |
| CNTN1 | g__Parvimonas | -0.037106018 | 0.770976551 | 0.852335885 |
| CNTN4 | g__Parvimonas | -0.020564102 | 0.871867036 | 0.913384514 |
| FABP4 | g__Parvimonas | -0.076564361 | 0.547622591 | 0.680155317 |
| IBSP | g__Parvimonas | -0.108055154 | 0.395382834 | 0.527177112 |
| IGHV3_43 | g__Parvimonas | 0.024889109 | 0.845221732 | 0.902663985 |
| IGHV3_52 | g__Parvimonas | 0.02284031 | 0.857824249 | 0.907314109 |
| IGHV4_59 | g__Parvimonas | 0.039610106 | 0.755985087 | 0.844247306 |
| IGJ | g__Parvimonas | -0.046667078 | 0.714232203 | 0.818441911 |
| IGKC | g__Parvimonas | 0.023599124 | 0.853152352 | 0.906731968 |
| IGKV1D_27 | g__Parvimonas | -0.014948641 | 0.906670952 | 0.932091632 |
| IGKV1D_33 | g__Parvimonas | 0.01646627 | 0.897244833 | 0.926914718 |
| IGKV1OR2_3 | g__Parvimonas | -0.006525803 | 0.959183682 | 0.967983532 |
| IGKV2D_26 | g__Parvimonas | 0.025040871 | 0.844289707 | 0.902663985 |
| IGKV3D_11 | g__Parvimonas | 0.026482618 | 0.83544625 | 0.900971447 |
| IGKV3D_20 | g__Parvimonas | 0.031490793 | 0.804890954 | 0.880975174 |
| MMP7 | g__Parvimonas | -0.049322928 | 0.698724949 | 0.809049941 |
| MMP9 | g__Parvimonas | 0.038699528 | 0.76142649 | 0.846029434 |
| TPH1 | g__Parvimonas | -0.1115457 | 0.380203865 | 0.522141965 |
| CASQ2 | g__Anaerostipes | 0.173809578 | 0.169580926 | 0.400363592 |
| CD36 | g__Anaerostipes | -0.140882064 | 0.266830097 | 0.435087235 |
| CNTN1 | g__Anaerostipes | 0.142097355 | 0.262684466 | 0.435087235 |
| CNTN4 | g__Anaerostipes | 0.151316957 | 0.232648492 | 0.420276528 |
| FABP4 | g__Anaerostipes | -0.06895058 | 0.588247101 | 0.715038823 |
| IBSP | g__Anaerostipes | -0.145307558 | 0.251943275 | 0.433027505 |
| IGHV3_43 | g__Anaerostipes | -0.148678272 | 0.240990823 | 0.420777627 |
| IGHV3_52 | g__Anaerostipes | -0.149412033 | 0.238650651 | 0.420730516 |
| IGHV4_59 | g__Anaerostipes | -0.131664195 | 0.299700083 | 0.448854614 |
| IGJ | g__Anaerostipes | -0.090160852 | 0.478625005 | 0.608656076 |
| IGKC | g__Anaerostipes | -0.13686931 | 0.280829034 | 0.435087235 |
| IGKV1D_27 | g__Anaerostipes | -0.087317529 | 0.492659691 | 0.622903057 |
| IGKV1D_33 | g__Anaerostipes | -0.137557211 | 0.278395306 | 0.435087235 |
| IGKV1OR2_3 | g__Anaerostipes | -0.115773688 | 0.362293522 | 0.510926762 |
| IGKV2D_26 | g__Anaerostipes | -0.110339273 | 0.385410155 | 0.522141965 |
| IGKV3D_11 | g__Anaerostipes | -0.15624518 | 0.217607709 | 0.420276528 |
| IGKV3D_20 | g__Anaerostipes | -0.164729288 | 0.193338035 | 0.417003605 |
| MMP7 | g__Anaerostipes | -0.180482214 | 0.153536651 | 0.397388979 |
| MMP9 | g__Anaerostipes | -0.230056925 | 0.067430131 | 0.353841981 |
| TPH1 | g__Anaerostipes | 0.232120627 | 0.064941216 | 0.353841981 |
| CASQ2 | g__Clostridium.XlVa | 0.137271062 | 0.279405965 | 0.435087235 |
| CD36 | g__Clostridium.XlVa | -0.151098901 | 0.23333021 | 0.420276528 |
| CNTN1 | g__Clostridium.XlVa | 0.076053114 | 0.550307484 | 0.680155317 |
| CNTN4 | g__Clostridium.XlVa | 0.138211289 | 0.276094263 | 0.435087235 |
| FABP4 | g__Clostridium.XlVa | -0.14029304 | 0.2688551 | 0.435087235 |
| IBSP | g__Clostridium.XlVa | -0.242994505 | 0.05302114 | 0.353841981 |
| IGHV3_43 | g__Clostridium.XlVa | -0.029304029 | 0.81819971 | 0.886718897 |
| IGHV3_52 | g__Clostridium.XlVa | -0.044368132 | 0.727749404 | 0.829558906 |
| IGHV4_59 | g__Clostridium.XlVa | -0.003663004 | 0.977082677 | 0.981544242 |
| IGJ | g__Clostridium.XlVa | -0.012087912 | 0.924473067 | 0.940355179 |
| IGKC | g__Clostridium.XlVa | -0.061401099 | 0.629818268 | 0.748973075 |
| IGKV1D_27 | g__Clostridium.XlVa | 0.016437729 | 0.897421977 | 0.926914718 |
| IGKV1D_33 | g__Clostridium.XlVa | -0.065064103 | 0.609494112 | 0.72874296 |
| IGKV1OR2_3 | g__Clostridium.XlVa | -0.078754579 | 0.536192388 | 0.670240485 |
| IGKV2D_26 | g__Clostridium.XlVa | -0.029990842 | 0.814014031 | 0.886549934 |
| IGKV3D_11 | g__Clostridium.XlVa | -0.041941392 | 0.742108122 | 0.837250189 |
| IGKV3D_20 | g__Clostridium.XlVa | -0.057371795 | 0.652494459 | 0.76764054 |
| MMP7 | g__Clostridium.XlVa | -0.184615385 | 0.144180128 | 0.396088009 |
| MMP9 | g__Clostridium.XlVa | -0.250869963 | 0.045553795 | 0.353841981 |
| TPH1 | g__Clostridium.XlVa | 0.099084249 | 0.435996961 | 0.564868166 |
| CASQ2 | g__Clostridium.XlVb | 0.248142427 | 0.048035474 | 0.353841981 |
| CD36 | g__Clostridium.XlVb | -0.19783617 | 0.117108149 | 0.353841981 |
| CNTN1 | g__Clostridium.XlVb | 0.150575307 | 0.234972786 | 0.420276528 |
| CNTN4 | g__Clostridium.XlVb | 0.173497896 | 0.170359246 | 0.400363592 |
| FABP4 | g__Clostridium.XlVb | -0.174274432 | 0.168424949 | 0.400363592 |
| IBSP | g__Clostridium.XlVb | -0.25650009 | 0.040763848 | 0.353841981 |
| IGHV3_43 | g__Clostridium.XlVb | -0.193485606 | 0.125548775 | 0.360776963 |
| IGHV3_52 | g__Clostridium.XlVb | -0.215398709 | 0.087385077 | 0.353841981 |
| IGHV4_59 | g__Clostridium.XlVb | -0.198019351 | 0.116762536 | 0.353841981 |
| IGJ | g__Clostridium.XlVb | -0.141187248 | 0.265784941 | 0.435087235 |
| IGKC | g__Clostridium.XlVb | -0.207453206 | 0.099997826 | 0.353841981 |
| IGKV1D_27 | g__Clostridium.XlVb | -0.209697181 | 0.096300291 | 0.353841981 |
| IGKV1D_33 | g__Clostridium.XlVb | -0.197675886 | 0.117411203 | 0.353841981 |
| IGKV1OR2_3 | g__Clostridium.XlVb | -0.170358924 | 0.178343782 | 0.400363592 |
| IGKV2D_26 | g__Clostridium.XlVb | -0.247684473 | 0.048462816 | 0.353841981 |
| IGKV3D_11 | g__Clostridium.XlVb | -0.217505298 | 0.084260499 | 0.353841981 |
| IGKV3D_20 | g__Clostridium.XlVb | -0.236258519 | 0.060173561 | 0.353841981 |
| MMP7 | g__Clostridium.XlVb | -0.175900169 | 0.164427515 | 0.400363592 |
| MMP9 | g__Clostridium.XlVb | -0.246081634 | 0.049983109 | 0.353841981 |
| TPH1 | g__Clostridium.XlVb | 0.201660087 | 0.110054401 | 0.353841981 |
| CASQ2 | g__Romboutsia | 0.178271216 | 0.158723098 | 0.400363592 |
| CD36 | g__Romboutsia | -0.098618119 | 0.43816943 | 0.564868166 |
| CNTN1 | g__Romboutsia | 0.202040711 | 0.109370611 | 0.353841981 |
| CNTN4 | g__Romboutsia | 0.212364712 | 0.092044656 | 0.353841981 |
| FABP4 | g__Romboutsia | -0.216385165 | 0.085910759 | 0.353841981 |
| IBSP | g__Romboutsia | -0.231143401 | 0.066110418 | 0.353841981 |
| IGHV3_43 | g__Romboutsia | -0.156432704 | 0.21704919 | 0.420276528 |
| IGHV3_52 | g__Romboutsia | -0.150731704 | 0.234481317 | 0.420276528 |
| IGHV4_59 | g__Romboutsia | -0.157168317 | 0.214867974 | 0.420276528 |
| IGJ | g__Romboutsia | -0.199512041 | 0.113975304 | 0.353841981 |
| IGKC | g__Romboutsia | -0.137559633 | 0.27838676 | 0.435087235 |
| IGKV1D_27 | g__Romboutsia | -0.211304837 | 0.093717525 | 0.353841981 |
| IGKV1D_33 | g__Romboutsia | -0.131605765 | 0.299916492 | 0.448854614 |
| IGKV1OR2_3 | g__Romboutsia | -0.112801658 | 0.374828866 | 0.522141965 |
| IGKV2D_26 | g__Romboutsia | -0.178156276 | 0.158996211 | 0.400363592 |
| IGKV3D_11 | g__Romboutsia | -0.111192504 | 0.381723683 | 0.522141965 |
| IGKV3D_20 | g__Romboutsia | -0.107123645 | 0.399493167 | 0.529448775 |
| MMP7 | g__Romboutsia | -0.204845236 | 0.104432861 | 0.353841981 |
| MMP9 | g__Romboutsia | -0.124226647 | 0.32806392 | 0.478221772 |
| TPH1 | g__Romboutsia | 0.182271111 | 0.149433203 | 0.396088009 |
| CASQ2 | g__Clostridium.XVIII | 0.258174805 | 0.039422204 | 0.353841981 |
| CD36 | g__Clostridium.XVIII | -0.105836208 | 0.405215139 | 0.533816351 |
| CNTN1 | g__Clostridium.XVIII | 0.067388078 | 0.596749014 | 0.717403186 |
| CNTN4 | g__Clostridium.XVIII | 0.13377379 | 0.291954668 | 0.446041853 |
| FABP4 | g__Clostridium.XVIII | -0.132514502 | 0.296562232 | 0.448854614 |
| IBSP | g__Clostridium.XVIII | -0.233481228 | 0.063341101 | 0.353841981 |
| IGHV3_43 | g__Clostridium.XVIII | -0.109597939 | 0.388630324 | 0.522141965 |
| IGHV3_52 | g__Clostridium.XVIII | -0.124183304 | 0.328234035 | 0.478221772 |
| IGHV4_59 | g__Clostridium.XVIII | -0.127645021 | 0.314823183 | 0.464839598 |
| IGJ | g__Clostridium.XVIII | -0.055872102 | 0.661016567 | 0.773530026 |
| IGKC | g__Clostridium.XVIII | -0.098428135 | 0.43905662 | 0.564868166 |
| IGKV1D_27 | g__Clostridium.XVIII | -0.10251296 | 0.420204285 | 0.550267516 |
| IGKV1D_33 | g__Clostridium.XVIII | -0.116752153 | 0.358223309 | 0.510926762 |
| IGKV1OR2_3 | g__Clostridium.XVIII | -0.143961244 | 0.256411028 | 0.434294992 |
| IGKV2D_26 | g__Clostridium.XVIII | -0.123121711 | 0.332418056 | 0.481131397 |
| IGKV3D_11 | g__Clostridium.XVIII | -0.117306028 | 0.355931795 | 0.510926762 |
| IGKV3D_20 | g__Clostridium.XVIII | -0.109551783 | 0.388831343 | 0.522141965 |
| MMP7 | g__Clostridium.XVIII | -0.147469116 | 0.244881465 | 0.424204113 |
| MMP9 | g__Clostridium.XVIII | -0.200225673 | 0.112660961 | 0.353841981 |
| TPH1 | g__Clostridium.XVIII | 0.204448967 | 0.105119875 | 0.353841981 |
| CASQ2 | g__Acidaminococcus | -0.303563037 | 0.014749032 | 0.353841981 |
| CD36 | g__Acidaminococcus | 0.360951566 | 0.003387693 | 0.248430854 |
| CNTN1 | g__Acidaminococcus | -0.294530215 | 0.018153749 | 0.353841981 |
| CNTN4 | g__Acidaminococcus | -0.298779061 | 0.016476711 | 0.353841981 |
| FABP4 | g__Acidaminococcus | 0.391572832 | 0.001375944 | 0.248430854 |
| IBSP | g__Acidaminococcus | 0.368268152 | 0.002752814 | 0.248430854 |
| IGHV3_43 | g__Acidaminococcus | 0.202756744 | 0.108093146 | 0.353841981 |
| IGHV3_52 | g__Acidaminococcus | 0.220762169 | 0.079604114 | 0.353841981 |
| IGHV4_59 | g__Acidaminococcus | 0.198089786 | 0.116629854 | 0.353841981 |
| IGJ | g__Acidaminococcus | 0.193723922 | 0.125074759 | 0.360776963 |
| IGKC | g__Acidaminococcus | 0.25924199 | 0.038586495 | 0.353841981 |
| IGKV1D_27 | g__Acidaminococcus | 0.153076223 | 0.227198825 | 0.420276528 |
| IGKV1D_33 | g__Acidaminococcus | 0.259723741 | 0.038214078 | 0.353841981 |
| IGKV1OR2_3 | g__Acidaminococcus | 0.291067633 | 0.019626281 | 0.353841981 |
| IGKV2D_26 | g__Acidaminococcus | 0.25674291 | 0.040567012 | 0.353841981 |
| IGKV3D_11 | g__Acidaminococcus | 0.225549564 | 0.073132564 | 0.353841981 |
| IGKV3D_20 | g__Acidaminococcus | 0.238767594 | 0.057423246 | 0.353841981 |
| MMP7 | g__Acidaminococcus | 0.316329425 | 0.010880425 | 0.353841981 |
| MMP9 | g__Acidaminococcus | 0.314161548 | 0.011467511 | 0.353841981 |
| TPH1 | g__Acidaminococcus | -0.304105006 | 0.014563502 | 0.353841981 |
| CASQ2 | g__Fusobacterium | 0.13561202 | 0.285313521 | 0.438943879 |
| CD36 | g__Fusobacterium | -0.221213685 | 0.078975005 | 0.353841981 |
| CNTN1 | g__Fusobacterium | 0.168778428 | 0.182465361 | 0.40547858 |
| CNTN4 | g__Fusobacterium | 0.153057425 | 0.22725658 | 0.420276528 |
| FABP4 | g__Fusobacterium | -0.186900011 | 0.139194788 | 0.390138934 |
| IBSP | g__Fusobacterium | -0.139783895 | 0.270613761 | 0.435087235 |
| IGHV3_43 | g__Fusobacterium | -0.219179896 | 0.081840152 | 0.353841981 |
| IGHV3_52 | g__Fusobacterium | -0.233338198 | 0.0635078 | 0.353841981 |
| IGHV4_59 | g__Fusobacterium | -0.216911439 | 0.085132269 | 0.353841981 |
| IGJ | g__Fusobacterium | -0.26220236 | 0.036344804 | 0.353841981 |
| IGKC | g__Fusobacterium | -0.25318068 | 0.043534732 | 0.353841981 |
| IGKV1D_27 | g__Fusobacterium | -0.207941907 | 0.099183301 | 0.353841981 |
| IGKV1D_33 | g__Fusobacterium | -0.279228826 | 0.025453394 | 0.353841981 |
| IGKV1OR2_3 | g__Fusobacterium | -0.224916225 | 0.073963786 | 0.353841981 |
| IGKV2D_26 | g__Fusobacterium | -0.211748743 | 0.093014007 | 0.353841981 |
| IGKV3D_11 | g__Fusobacterium | -0.194904797 | 0.122746029 | 0.360776963 |
| IGKV3D_20 | g__Fusobacterium | -0.204161145 | 0.105621064 | 0.353841981 |
| MMP7 | g__Fusobacterium | -0.186482824 | 0.140095344 | 0.390138934 |
| MMP9 | g__Fusobacterium | -0.091390142 | 0.472623869 | 0.604518902 |
| TPH1 | g__Fusobacterium | 0.109459577 | 0.389233102 | 0.522141965 |
