# Supplementary material for: Exploration of Crucial Mediators for Carotid Atherosclerosis Pathogenesis Through Integration of Microbiome, Metabolome, and Transcriptome
Source: Front Physiol. 2021 May 24;12:645212. doi: 10.3389/fphys.2021.645212 (PMC8181762; doi:10.3389/fphys.2021.645212)
Supplement: Supplementary Table 11 — AUCs for differentially enriched gut microbiota, metabolites, and DEGs. [file Table_11.DOCX]

**Table S11. AUCs for differentially enriched gut microbiota, metabolites and DEGs.**

| **Variables** | **AUC** | **SE** | ***p*-value** | **95% Confidence Interval** | |
| --- | --- | --- | --- | --- | --- |
|  |  |  |  | **Lower Bound** | **Upper Bound** |
| ***Gut microbiota*** |  |  |  |  |  |
| g__Gemella | 0.628 | 0.07 | 0.079 | 0.490 | 0.766 |
| g__Lactobacillus | 0.707 | 0.066 | 0.004 | 0.579 | 0.835 |
| g__Christensenella | 0.593 | 0.071 | 0.202 | 0.453 | 0.733 |
| g__Parvimonas | 0.610 | 0.071 | 0.129 | 0.471 | 0.749 |
| g__Anaerostipes | 0.655 | 0.070 | 0.033 | 0.519 | 0.792 |
| g__Clostridium_XlVa | 0.683 | 0.067 | 0.012 | 0.551 | 0.814 |
| g__Clostridium_XlVb | 0.755 | 0.061 | 0.000 | 0.636 | 0.874 |
| g__Romboutsia | 0.754 | 0.060 | 0.000 | 0.636 | 0.872 |
| g__Clostridium_XVIII | 0.689 | 0.067 | 0.009 | 0.558 | 0.821 |
| g__Acidaminococcus | 0.656 | 0.069 | 0.032 | 0.521 | 0.791 |
| g__Fusobacterium | 0.629 | 0.070 | 0.075 | 0.492 | 0.766 |
| ***Metabolites*** |  |  |  |  |  |
| Ethanolamine | 0.999 | 0.002 | 0.000 | 0.996 | 1.000 |
| Gly_Pro | 1.000 | 0.000 | 0.000 | 1.000 | 1.000 |
| Propoxur | 1.000 | 0.000 | 0.000 | 1.000 | 1.000 |
| Homocitrate | 0.113 | 0.039 | 0.000 | 0.036 | 0.190 |
| Alpha_N_Phenylacetyl_L_glutamine | 0.178 | 0.053 | 0.000 | 0.074 | 0.282 |
| Diethylcarbamazine | 0.191 | 0.055 | 0.000 | 0.083 | 0.299 |
| Dimethylbenzimidazole | 0.104 | 0.037 | 0.000 | 0.031 | 0.176 |
| Eicosapentaenoic acid | 0.845 | 0.052 | 0.000 | 0.744 | 0.946 |
| Decanoyl_L_carnitine | 0.887 | 0.041 | 0.000 | 0.807 | 0.967 |
| 3_Methoxy_4_Hydroxyphenylglycol Sulfate | 0.190 | 0.055 | 0.000 | 0.082 | 0.299 |
| O_Desmethylnaproxen | 0.923 | 0.039 | 0.000 | 0.846 | 1.000 |
| Salicylic acid | 0.213 | 0.057 | 0.000 | 0.102 | 0.324 |
| 3_Aminopropanesulphonic Acid | 0.419 | 0.072 | 0.265 | 0.278 | 0.560 |
| 6_Hydroxynicotinic acid | 0.332 | 0.067 | 0.021 | 0.200 | 0.464 |
| Formylanthranilic acid | 0.130 | 0.044 | 0.000 | 0.043 | 0.217 |
| Xanthopterin | 0.912 | 0.041 | 0.000 | 0.833 | 0.992 |
| N1_Methyl_4_pyridone_3_carboxamide | 0.955 | 0.023 | 0.000 | 0.911 | 1.000 |
| 3_Hydroxydodecanoic acid | 0.827 | 0.052 | 0.000 | 0.726 | 0.929 |
| Salicyluric acid | 0.246 | 0.062 | 0.000 | 0.125 | 0.367 |
| Phenylacetylglycine | 0.128 | 0.045 | 0.000 | 0.040 | 0.216 |
| D_Biotin | 0.955 | 0.026 | 0.000 | 0.904 | 1.000 |
| 5_10_methylene_THF | 0.844 | 0.052 | 0.000 | 0.742 | 0.945 |
| ***DEGs*** |  |  |  |  |  |
| CASQ2 | 0.868 | 0.045 | 0.000 | 0.780 | 0.956 |
| CD36 | 0.799 | 0.055 | 0.000 | 0.691 | 0.907 |
| CNTN1 | 0.853 | 0.048 | 0.000 | 0.759 | 0.946 |
| CNTN4 | 0.868 | 0.046 | 0.000 | 0.777 | 0.959 |
| FABP4 | 0.854 | 0.05 | 0.000 | 0.755 | 0.952 |
| IBSP | 0.860 | 0.047 | 0.000 | 0.768 | 0.952 |
| IGHV3_43 | 0.827 | 0.052 | 0.000 | 0.725 | 0.929 |
| IGHV3_52 | 0.832 | 0.051 | 0.000 | 0.732 | 0.932 |
| IGHV4_59 | 0.814 | 0.054 | 0.000 | 0.708 | 0.921 |
| IGJ | 0.808 | 0.055 | 0.000 | 0.701 | 0.914 |
| IGKC | 0.821 | 0.053 | 0.000 | 0.718 | 0.924 |
| IGKV1D_27 | 0.792 | 0.056 | 0.000 | 0.682 | 0.902 |
| IGKV1D_33 | 0.829 | 0.051 | 0.000 | 0.729 | 0.929 |
| IGKV1OR2_3 | 0.825 | 0.053 | 0.000 | 0.722 | 0.928 |
| IGKV2D_26 | 0.808 | 0.053 | 0.000 | 0.703 | 0.912 |
| IGKV3D_11 | 0.815 | 0.054 | 0.000 | 0.709 | 0.922 |
| IGKV3D_20 | 0.819 | 0.053 | 0.000 | 0.715 | 0.923 |
| MMP7 | 0.779 | 0.057 | 0.000 | 0.667 | 0.892 |
| MMP9 | 0.806 | 0.055 | 0.000 | 0.698 | 0.913 |
| TPH1 | 0.830 | 0.050 | 0.000 | 0.732 | 0.928 |
